# New Monoterpenoid Indole Alkaloids as Potential Neuroprotective Agents from *Uncaria hirsuta* Haviland

**DOI:** 10.3390/molecules31122053

**Published:** 2026-06-11

**Authors:** Xin-Yue Huang, Jia Cui, Wen-Ling Wang, Hui-Zhen Zhou, Yu-Chen Jiang, Xiao He, Hu-Lan Chen, Li-Mei Li

**Affiliations:** 1 College of Pharmacy and Food, Southwest Minzu University, Chengdu 610041, China; 17882478586@qq.com (X.-Y.H.); 19822912701@163.com (J.C.); 1215120113@qq.com (W.-L.W.); 2858351309@qq.com (Y.-C.J.); 965535198@qq.com (X.H.); 2 College of Pharmacy, Chengdu University of Traditional Chinese Medicine, Chengdu 611137, China; 1312161673@qq.com (H.-Z.Z.); hlan999@126.com (H.-L.C.)

**Keywords:** *Uncaria hirsuta* Havil., Rubiaceae, monoterpene indole alkaloid, absolute configuration, neuroprotective activity

## Abstract

Nineteen monoterpene indole alkaloids, including twelve new ones, were successfully isolated and identified from the stems and leaves of *Uncaria hirsuta* (Havil.). The planar structures were elucidated by nuclear magnetic resonance (NMR), high-resolution mass (HRMS), and ultraviolet (UV) analyses. The absolute configurations of new compounds were determined using electron circular dichroism calculations in conjunction with NMR calculations. The acetylcholinesterase inhibitory activity of the isolated compounds was evaluated in vitro. In further biological evaluation, the isolated compounds were evaluated for their neuroprotective effects on HT22 neuronal cells. Six compounds demonstrated significant protective activity. Their intracellular reactive oxygen species (ROS) levels were measured using the DCFH-DA fluorescent probe, which markedly attenuated glutamate-induced ROS accumulation. The results not only enrich the knowledge on the structural diversity of monoterpene indole alkaloids but also offer substantial evidence for further pharmacological exploration.

## 1. Introduction

Gouteng, a traditional Chinese medicine, is typically used to treat internal stirring of liver wind, epileptic convulsions, hypertension, headache, dizziness, and similar symptoms [1]. Numerous Chinese herbal formulations based on Gouteng, such as Tianma–Gouteng decoction [2,3], Yigan powder [4], and Gouteng–Baitouweng [5], have demonstrated clinical efficacy and are widely used in medical applications [6]. The source plants of Gouteng include several *Uncaria* plants, such as *Uncaria rhynchophylla*, *Uncaria macrophylla*, and *Uncaria hirsuta*. Their medicinal parts are hooked, branched stems. The predominant bioactive components in *Uncaria* are monoterpenoid indole alkaloids, which mediate most of the known pharmacological activities of Gouteng [7,8]. Notably, hirsutine (HTE), isolated from *U. rhynchophylla*, demonstrates antihypertensive, antiarrhythmic, and platelet-production-promoting effects [9,10]. Rhynchophylline (RN) and isorhynchophylline (IRN) regulate the nervous and cardiovascular systems and are used to treat diabetes and cancer [11,12,13,14].

The neuroprotective mechanisms of alkaloids from *Uncaria* plants have also been enhanced in recent years. HTE exhibits anti-Parkinsonian effects through high-affinity binding to UCHL1 and subsequent mitophagy activation [8]. RN and IRN protect PC12 cells against Aβ-induced neurotoxicity by mitigating oxidative stress, Ca^2+^ overload, and tau hyperphosphorylation [15]. IRN additionally reduces Aβ plaques and tau pathology in TgCRND8 mice by suppressing neuroinflammation via inhibition of the JNK pathway [16]. These findings demonstrate the significant neuroprotective potential of *Uncaria* species as promising therapeutic candidates for treating neurodegenerative disorders such as Alzheimer’s disease and Parkinson’s disease [17].

*U. hirsuta* is one of the source plants of Gouteng. Currently, more than 90 chemical constituents, including a variety of flavonoids and alkaloids, such as RN, IRN, and HTE [18,19], have been found in the stems and leaves of *U. hirsuta*. The extracts of *U. hirsuta* can reverse memory deficits, and two functionally important active constituents (5β-carboxystrictosidine and chlorogenic acid) have been isolated from this plant material, both of which exhibit neuroprotective potential through multiple modes of action [20]. However, other neuroprotective constituents in this plant are still unclear. Given the therapeutic potential of *U. hirsuta*, the discovery of monoterpene indole alkaloids and the exploration of their neuroprotective activity remain scientifically valuable.

## 2. Results and Discussion

### 2.1. Structure Identification of Compounds **1**–**19**

**Figure 1:**
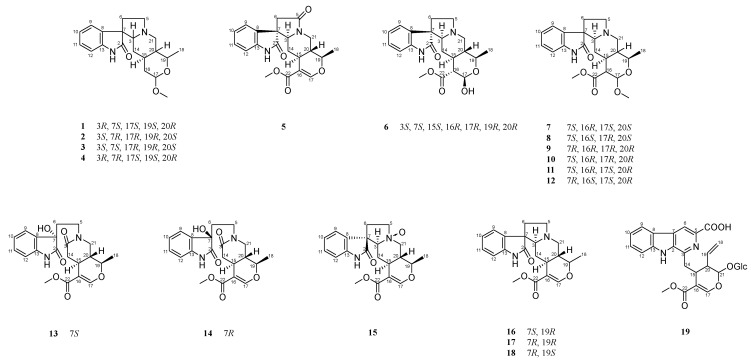
Structures of compounds **1**–**19**.

**Figure 2:**
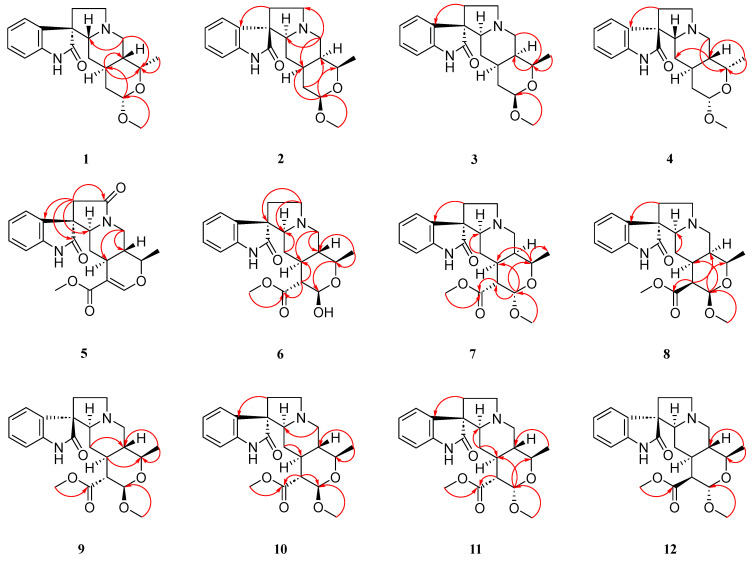
Key HMBC correlations of compounds **1**–**12**.

Compound **1** was obtained as a whitish, noncrystalline solid. It exhibited a positive reaction with Dragendoff’s reagent. The molecular formula was determined to be C_20_H_26_N_2_O_3_ (*m*/*z* 343.2023, [M + H]^+^) on the basis of HRESIMS analysis. UV spectroscopic analysis revealed two distinct absorption maxima at 252 nm and 208 nm, indicating the presence of an oxindole chromophore [21]. The ^1^H NMR spectrum (Table 1) displayed characteristic signals for an indole moiety. These included an indole-NH proton at *δ*_H_ 7.53 (1H, s) and aromatic protons corresponding to an unsubstituted indole ring at *δ*_H_ 7.20 (2H, overlap), 7.05 (1H, t, *J* = 7.7 Hz), and 6.85 (1H, d, *J* = 7.9 Hz); a methoxy group at *δ*_H_ 3.45 (3H, s); and a methyl group at *δ*_H_ 1.23 (3H, *J* = 6.2 Hz). The ^13^C NMR spectrum (Table 1) revealed twenty distinct carbon signals, comprising one carbonyl carbon (*δ*_C_ 180.9), three quaternary carbons (*δ*_C_ 55.8, 133.5, 140.9), one methyl group (*δ*_C_ 19.0), five methylene groups (*δ*_C_ 54.9, 34.5, 31.5, 38.4, 54.2), nine methine carbons (*δ*_C_ 74.8, 122.8, 123.3, 128.2, 109.4, 37.8, 45.5, 102.6, 73.8), and one methoxy group (*δ*_C_ 56.2). Comparative analysis with the known compound uncarine B (**17**) (Figure 1) [19] revealed structural similarity, particularly in the pentacyclic alkaloid framework. However, obvious differences were observed in the chemical environments of C-16 and C-17. Firstly, the absence of characteristic double-bond carbon signals (*δ*_C_ 155.6 and 108.3) in the ^13^C NMR spectrum of compound **1** suggested a lack of unsaturation at these positions. Furthermore, compound **1** contained a methoxy group at C-17 instead of a -COOCH_3_ at C-16 in uncarine B. The key HMBC correlation signal from OMe (*δ*_H_ 3.45) to C-17 (*δ*_C_ 102.6) confirmed the above deduction (Figure 2).

**Figure 3:**
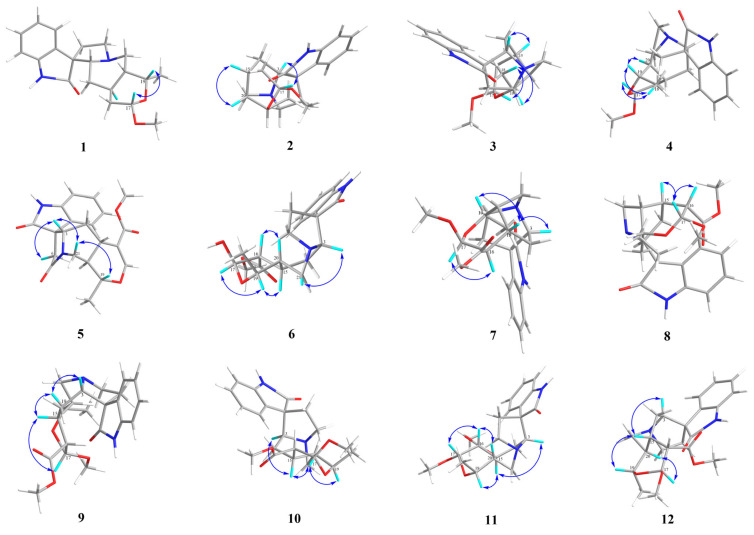
Key NOESY correlations of compounds **1**–**12**.

**Figure 4:**
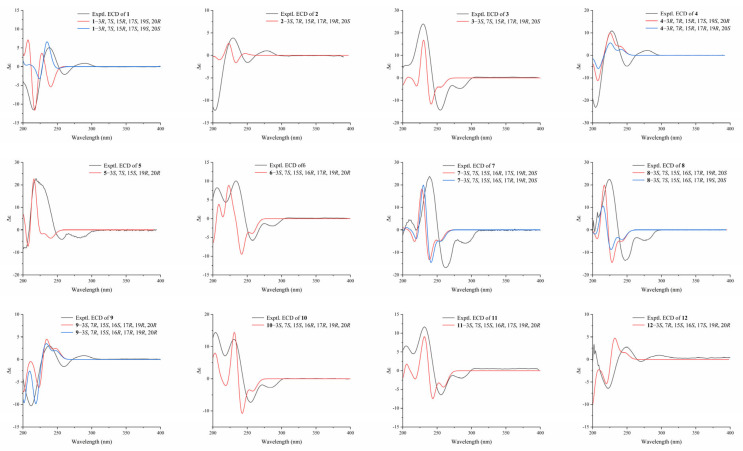
Experimental and calculated ECD spectra of compounds **1**–**12**.

**Figure 5:**
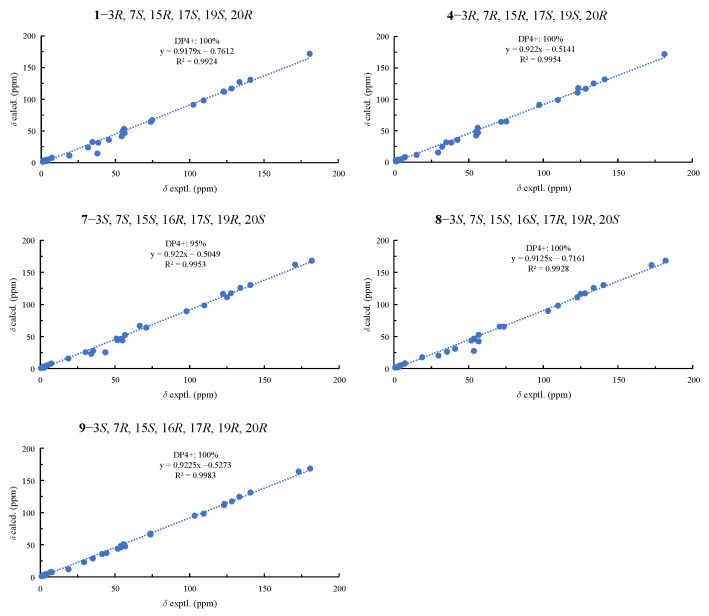
Diastereomeric Probability 4 (DP4+) analysis of compounds **1**, **4**, and **7**–**9**.

The ^1^H NMR and ^13^C NMR spectroscopic data of compounds **2**–**4** exhibit substantial similarity to those of compound **1** (Table 1 and Table 2). Meanwhile, these four compounds share identical molecular weights and molecular formulas, suggesting that they may be stereoisomers (or diastereomers) characterized by the same planar structure and multiple chiral carbon centres. Firstly, analysis of the NOESY spectra of compounds **1**–**4** revealed the following spatial proximity: H-17/H-18 in compound **1**, H-17/H-19 in **2**, H-3/H-21 and H-21/H-19 in **3**, and H-19/H-20 in **4** (Figure 3). Biogenetically, the absolute configuration of the asymmetric centre at the C-15 position is fixed in the *S* form [22,23]. Therefore, the absolute configuration of the remaining chiral carbon atoms in compounds **1**–**4** was established through comparison of the experimental and calculated ECD spectra (Figure 4). Specifically, compound **2** was assigned the absolute configurations 3*S*, 7*R*, 15*R*, 17*R*, 19*R*, and 20*S*, while compound **3** was assigned 3*S*, 7*S*, 15*R*, 17*R*, 19*R*, and 20*S* (Figure 1). For compounds **1** and **4**, ECD calculations yielded two candidate configurations, which were further refined using the computational DP4+ probability method and NMR GIAO calculations for representative conformations [24,25]. The relative probabilities of stereoisomers 1a/1b (C20) and 7a/7b (C17/C19/C20) were assessed by DP4+ calculations. The results indicated that the absolute configurations of compound **1** were 3*R*, 7*S*, 15*R*, 17*S*, 19*S*, and 20*R* (Figure 1), with a 100% DP4+ probability (Figure 5), whereas compound **4** was determined to have absolute configurations 3*R*, 7*R*, 15*R*, 17*S*, 19*S*, and 20*R* (Figure 1), with a 100% DP4+ probability (Figure 5). Consequently, compounds **1**–**4** were identified and named uncahirsutines A–D, respectively.

Compound **5** was first characterized using HRESIMS, which indicated a molecular formula of C_21_H_22_N_2_O_5_ (*m*/*z* 383.1605, [M + H]^+^). The UV spectrum exhibited maximum absorption bands at 234 and 206 nm. In the ^1^H-NMR spectrum (Table 1), the low-field region displayed four aromatic proton signals at *δ*_H_ 7.23 (t, *J* = 7.7 Hz), 7.15 (d, *J* = 7.4 Hz), 7.01 (t, *J* = 7.5 Hz), and 6.92 (d, *J* = 7.8 Hz), corresponding to carbon signals (Table 2) at *δ*_C_ 130.1, 125.1, 123.8, and 111.6, respectively. These data suggested the presence of an unsubstituted aromatic ring within an oxidized indole moiety. Additionally, analysis of the spectral data revealed three pairs of methylene proton signals, including *δ*_H_ 0.70 (q, *J* = 12.1 Hz) and *δ*_H_ 2.16 (dt, *J* = 12.7, 3.3 Hz), *δ*_H_ 2.96 (d, *J* = 17.3 Hz) and *δ*_H_ 2.46 (d, *J* = 16.8 Hz), and *δ*_H_ 4.19 (dd, *J* = 12.8, 4.2 Hz) and *δ*_H_ 2.63 (dd, *J* = 14.0, 10.8 Hz), which correlated with the carbon signals at *δ*_C_ 31.5, *δ*_C_ 42.5, and *δ*_C_ 42.7, respectively. A methyl at *δ*_H_ 1.34 (d, *J* = 6.2 Hz), a methoxy at *δ*_H_ 3.53, and an alkene hydrogen at *δ*_H_ 7.48 were also present in the ^1^H NMR spectra. Further comparative analyses revealed that the ^13^C NMR and ^1^H NMR signals of compound **5** closely resembled those of uncarine A [26] and uncarine B [26], with the most notable difference being the presence of a carbonyl signal at *δ*_C_ 173.9 in compound **5**. The molecular weight of compound **5** is 14 units greater than that of uncarine B (**17**), suggesting the replacement of a methylene group with a carbonyl group. In the HMBC spectrum, H-6*α* (*δ*_H_ 2.96) correlated with *δ*_C_ 173.9 as well as C-7 (*δ*_C_ 53.4) and C-8 (*δ*_C_ 131.7) (Figure 2). In addition, H-6*β* (*δ*_H_ 2.46) correlated with *δ*_C_ 173.9, C-3 (*δ*_C_ 64.3), and C-7 (*δ*_C_ 53.4). These findings collectively revealed the carbonyl substitution position at C-5. These data confirmed the planar structure of compound **5**, which contains five chiral centres at C-3, C-7, C-15, C-19, and C-20. The correlation between H-15 and H-19 was illustrated in the NOESY correlation. The *S*-configuration of H-15 was also biogenetically determined. The stereochemical assignment was achieved using experimental and calculated ECD spectral correlation analysis (Figure 4). The remaining chiral centres were assigned as follows: 3*S*, 7*S*, 19*R*, and 20*R* (Figure 1). Thus, compound **5** was elucidated and named uncahirsutine E.

Compound **6** was obtained as a whitish, noncrystalline solid and exhibited a positive reaction with Dragendoff’s reagent. HRESIMS analysis revealed its molecular formula as C_21_H_26_N_2_O_5_ (*m*/*z* 387.1917, [M + H]^+^). Its UV spectrum exhibited characteristic absorption maxima at 251 and 212 nm. Comparative analysis of the ^1^H NMR and ^13^C NMR data (Table 1 and Table 2) of compound **6** and uncarine B (**17**) [26] revealed structural similarity. The primary distinction is the absence of double-bonded signals in compound **6**, which are characteristic of uncarine B (**17**). HMBC spectral analysis revealed that H-16 (*δ*_H_ 2.06) correlated to C-15 (*δ*_C_ 40.6), C-17 (*δ*_C_ 96.4), and C-22 (*δ*_C_ 172.2), confirming that the -COOCH_3_ group was attached to C-16. Moreover, the HMBC correlation from H-19 (*δ*_H_ 3.44) to C-17 (*δ*_C_ 96.4) supported that the hydroxyl group is located at C-17 (Figure 2). The relative configuration of compound **6** was established by NOESY correlations (H-3/H-15/H-19), whereas its absolute stereochemistry was confirmed by the strong match between the experimental and calculated ECD spectra. (Figure 1 and Figure 4). Compound **6** was identified and named uncahirsutine F.

Compound **7** was also obtained as a whitish, noncrystalline solid. HRESIMS analysis established its molecular formula as C_22_H_28_N_2_O_5_ (*m*/*z* 401.2089, [M + H]^+^), whereas its UV spectrum displayed maximum absorption bands at 251 and 209 nm. The ^1^H and ^13^C NMR data of compound **7** are very similar to those of compound **6**. The only difference is one additional -OCH_3_ present in compound **7** (Table 1 and Table 2). In the HMBC spectrum (Figure 2), correlations from the methoxy signals at *δ*_H_ 3.27 (s, 3H) to C-17 (*δ*_C_ 97.9), H-17 (*δ*_H_ 4.80, d, *J* = 3.8 Hz) to C-15 (*δ*_C_ 34.1), C-16 (*δ*_C_ 51.1), and C-19 (*δ*_C_ 66.6), and H-16 (*δ*_H_ 2.34, dd, *J* = 11.6, 3.3 Hz) to C-15 (*δ*_C_ 34.1) and C-17 (*δ*_C_ 97.9) confirmed the attachment of the methoxy group to C-17.

The ^1^H NMR and ^13^C NMR data (Table 1 and Table 2) of compounds **8**–**12** were very similar to those of compound **7**, and these compounds had the same molecular weight and molecular formula. This observation suggested that these six compounds are likely stereoisomers, possessing the same planar structure but differing in the configuration of multiple chiral carbon centres. On the basis of biogenetic principles, the configuration of H-15 was determined to be *S*, leaving six additional chiral centres at C-3, C-7, C-16, C-17, C-19, and C-20, theoretically giving rise to 64 possible diastereomeric forms. The stereochemical configurations of compounds **10**–**12** were elucidated using NOESY spectral data combined with ECD calculations (Figure 4). The absolute stereochemistry of compounds **7**–**9** was assigned using TDDFT-ECD computational analysis and further supported by ML-J-DP4+ probabilistic analysis (Figure 5). For compound 7, the DP4+ probability of 95% indicates high confidence in the assigned structure. The remaining 5% probability corresponds to the C-16 (configuration at C-16 inverted from *R* to *S*) and C-17 epimers (configuration at C-17 inverted from *S* to *R*). This alternative structure was one of the candidates retained after ECD pre-screening (as shown in Figure 4) because its calculated ECD spectrum was similar to the experimental data. The DP4+ analysis revealed subtle but significant differences in the calculated chemical shifts for the C-16 and C-17 methyl groups, leading to a lower overall probability for this epimer (Figure 1 and Figure 4). This comprehensive approach allowed for the precise assignment of stereochemistry across this series of compounds (Figure 1). Compounds **7**–**12** were finally identified and named uncahirsutines G–L, respectively.

In addition, seven known compounds were obtained and determined to be hirsutanine D (**13**) [19], hirsutanine E (**14**) [19], uncarine B *N*-oxide (**15**) [19], uncarine A (**16**) [26], uncarine B (**17**) [26], mitraphylline (**18**) [26], and deoxycordifoline (**19**) [27].

### 2.2. AChE Inhibitory Activity

Given that acetylcholine deficiency and impairment-related neurotransmission are core pathological mechanisms of neurodegenerative diseases, an acetylcholinesterase (AChE) inhibition assay was initially performed on all compounds in this study. Compounds **1**–**19** were evaluated for their ability to inhibit AChE activity using an adapted Ellman’s assay protocol, with huperzine A (IC_50_ = 114 ± 0.414 μM) serving as the reference standard. Only compound **5** exhibited potential inhibitory activity (IC_50_ = 680 ± 0.02 μM).

### 2.3. Cytotoxicity

**Figure 6:**
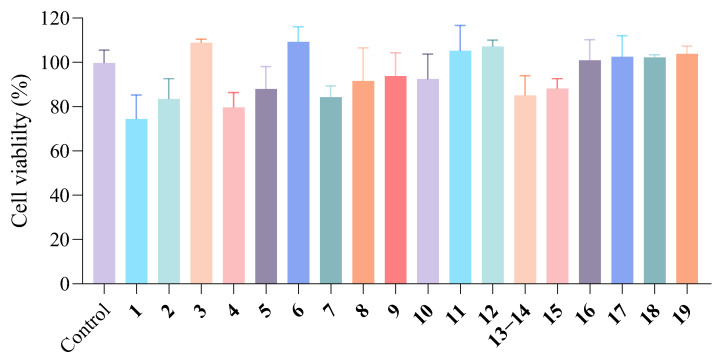
Cytotoxicity screening of compounds **1**–**19** at 40 μM in HT22 cells.

The cytotoxicity of all isolated compounds (**1**–**19**) was evaluated in vitro against mouse hippocampal neuronal HT22 cells. As illustrated in Figure 6, compounds **1** and **4** demonstrated cytotoxicity at 40 μM, reducing cell viability to below 80%. In contrast, the remaining compounds did not exhibit obvious cytotoxicity at this concentration.

### 2.4. Neuroprotective Activity

**Figure 7:**
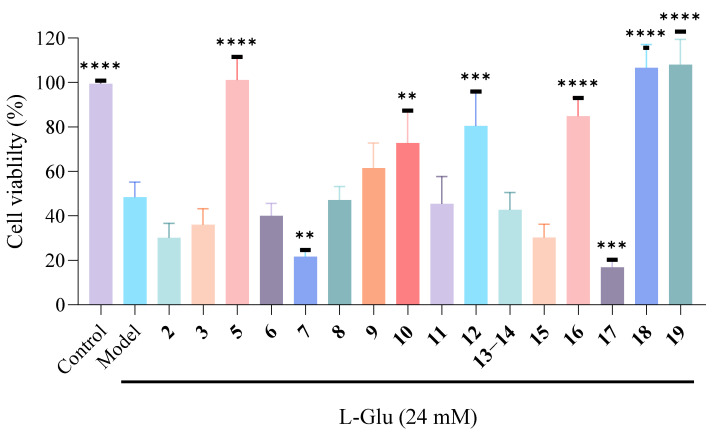
Neuroprotective effects of compounds **2**, **3**, and **5**–**19** at 40 μM on L-glutamate-induced cytotoxicity in HT22 cells. *n* = 4, ** *p* < 0.01, *** *p* < 0.001, **** *p* < 0.0001 compared to L-glutamate group.

Compounds (**2**, **3**, and **5**–**19**) were investigated for their neuroprotective effects against glutamate-induced cell death in mouse hippocampal HT22 cells for the first time (Figure 7). Initial validation established that exposure to 24 mM L-glutamate for 24 h induced severe injury in HT22 cells, reducing cell viability to 51.1 ± 0.1% compared to the control group, which confirmed a robust model for assessing neuroprotection.

In the subsequent activity assay, HT22 cells were co-treated with 24 mM L-glutamate and the tested compounds at 40 μM for 24 h. Cell viability was then analyzed using the CCK-8 assay. Notably, pre-treatment with compounds **5**, **10**, **12**, **16**, **18**, and **19** conferred significant protection against glutamate-induced cytotoxicity. These active compounds attenuated the detrimental effects of glutamate to varying degrees, resulting in a marked and statistically significant increase in cell survival rates compared to the model group. The results clearly indicated that these compounds are promising candidates for further investigation into their neuroprotective mechanisms.

### 2.5. Intracellular ROS Level Measurement

**Figure 8:**
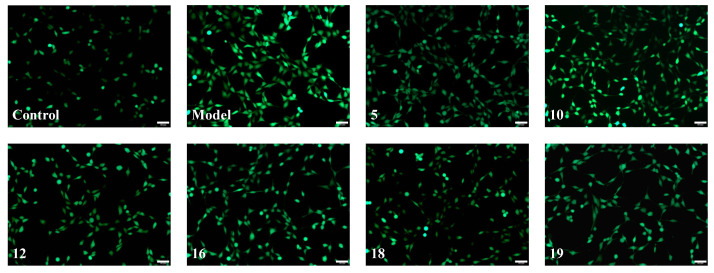
Effects of compounds **5**, **10**, **12**, **16**, **18**, and **19** (40 μM, 24 h) on intracellular ROS generation in L-glutamate-treated HT22 cells. Intracellular ROS levels were assessed using the DCFH-DA fluorescent probe.

To further investigate whether the neuroprotective effects were associated with the attenuation of oxidative stress, intracellular reactive oxygen species (ROS) levels were measured using the fluorescent probe 2′,7′-dichlorodihydrofluorescein diacetate (DCFH-DA) (Figure 8). In this assay, cells in the control group without glutamate injury showed low basal levels of ROS, corresponding to weak fluorescence intensity. Exposure to 24 mM glutamate significantly increased ROS production, as indicated by strong green fluorescence in the model group, confirming that glutamate-induced cytotoxicity is closely related to oxidative stress. However, pretreatment with the active compounds (**5**, **10**, **12**, **16**, **18**, and **19**) markedly reduced the glutamate-elevated ROS levels, as evidenced by decreased fluorescence signals. These results suggested that the neuroprotective activity of these compounds may be attributed, at least in part, to their ability to mitigate intracellular ROS accumulation and oxidative damage.

## 3. Materials and Methods

### 3.1. General Experimental Procedures

All evaporation procedures were performed using a Büchi R-100 rotary evaporator (Büchi Labortechnik AG, Flawil, Switzerland). Optical rotation measurements were conducted on a PerkinElmer 341 instrument (PerkinElmer, Waltham, MA, USA). The UV spectra were recorded in methanol using a Persee TU-1901 dual-beam UV-visible spectrophotometer (Persee, Beijing, China). ECD spectra were acquired on an Applied Photophysics Chirascan spectropolarimeter in methanol at 25 °C under a constant nitrogen flow (Applied Photophysics Ltd., Leatherhead, UK). NMR spectra were recorded with a Bruker Ascend 400 and a Bruker Ascend 600. HRESIMS data were collected on a Bruker MicrOTOF-QII. Column chromatography was performed using ODS reversed-phase C18 (YMC Co., Kyoto, Japan) and Sephadex LH-20 molecular sieves (GE Healthcare, Uppsala, Sweden). Semi-preparative high-performance liquid chromatography (HPLC) was performed on a Waters 2695 separation module equipped with two columns: an analytical Cosmosil 5C_18_-MS-II column (5 μm, 4.6 × 250 mm; Nacalai Tesque, Kyoto, Japan) and a prepared SunFire™ C18 OBD™ Prep column (10 μm, 10 × 250 mm; Waters Corp., Milford, MA, USA). An Agilent 1260 Infinity system was used for preparative HPLC equipped with an Agilent Pursuit XRs 5 C18 column (10 µm, 250 × 21.2 mm). All solvents and reagents were obtained from commercial sources based on their required purity grades: industrial-grade organic solvents from Chengdu LiXinHe Chemical Co., Ltd. (Chengdu, China); analytical-grade organic solvents from Chengdu Kelong Chemical Reagent Company (Chengdu, China); and HPLC-grade organic solvents from Anhui Tiandi High-Purity Solvent Co., Ltd. (Anqing, China).

### 3.2. Processing and Storage of Plant Material

The fresh hooked branches and leaves of *Uncaria hirsuta* Havil. were collected in October 2018 from Pu Lu Township, Lipu County, located in Guangxi Province, China. The voucher sample (No. LMUH1810) was authenticated by associate professor Luyang Lv and preserved at the College of Pharmacy and Food, Southwest Minzu University. To minimize degradation caused by enzymes, heat, or sunlight, the plant materials were returned to the laboratory immediately. They were thoroughly rinsed with water to remove soil and debris; cut into small pieces; and air-dried in a cool, shaded, and well-ventilated area away from direct sunlight. The dried stems and leaves of *U. hirsuta* (20.0 kg) were ground and subjected to reflux extraction 3 times (each 3 h) using 90% MeOH in H_2_O (100 L) at 50 °C. The total extract was obtained by combining the extracts and recovering the solvent under reduced pressure. First, the total extract was dissolved and dispersed in a 4% aqueous solution of HCl. Then, the phenolic components were removed through three extractions with EtOAc (10 L). Afterwards, the aqueous solution was adjusted to a pH of 10 using ammonia and partitioned with CHCl_3_ (10 L) three times. The extracts were combined, and the solvent was recovered to obtain a chloroform extract (200.0 g). Immediately after concentration, this extract was stored in a sealed, amber glass vial under an inert atmosphere of nitrogen gas and kept at −80 °C to prevent oxidation, photodegradation, and microbial contamination.

### 3.3. Extraction and Isolation

The chloroform extract was subjected to macroporous resin D101 fractionation, eluted using a solvent system composed of MeOH/H_2_O (25–100%), and subsequently combined into four fractions (A–D) according to the results of the TLC analysis. Fr. A (2.0 g) was separated using normal-phase silica gel column chromatography with a CHCl_3_/MeOH gradient (100:1–1:1) to obtain Fr. A1 (16.6 mg). Subsequent semipreparative HPLC purification of Fr. A1 (MeOH/H_2_O, 54%, 248 nm, 1 mL∙min^−1^) afforded compounds **1** (*t*_R_ = 26.0 min, 1.8 mg) and **2** (*t*_R_ = 42.0 min, 4.4 mg). Fr. B (7.0 g) was subjected to ODS reversed-phase chromatography (MeOH/H_2_O, 10–100%) and combined into three fractions (B1–B3) based on TLC detection. Fr. B1 (845.0 mg) was repeatedly prepared using HPLC (MeOH/H_2_O, 45%, 248 nm, 3 mL∙min^−1^) to yield compounds **3** (*t*_R_ = 36.1 min, 1.0 mg), **4** (*t*_R_ = 36.2 min, 1.0 mg), and **6** (*t*_R_ = 54.0 min, 1.0 mg). Fr. B2 produced compound **5** (5.0 mg) after Sephadex LH-20 (MeOH) separation and crystallization. Fr. B3 (30.0 mg) was further purified by HPLC (CH_3_CN/H_2_O, 35%, 248 nm, 3 mL∙min^−1^) to obtain compounds **13** and **14** (*t*_R_ = 25.6 min, 5.0 mg). Fr. C (12.0 g) was initially separated into four subfractions, namely, C1–C4, using silica gel chromatography (CHCl_3_/MeOH, 100:1–1:1). ODS (MeOH/H_2_O, 5–50%) and HPLC (MeOH/H_2_O, 67%, 248 nm, 8 mL∙min^−1^) chromatography of Fr. C1 (0.4 g) yielded compounds **7** (*t*_R_ = 21.7 min, 30.0 mg) and **8** (*t*_R_ = 28.9 min, 40.0 mg). Sequential silica gel (CHCl_3_/MeOH, 50:1–5:1) and HPLC (MeOH/H_2_O, 38%, 248 nm, 4 mL∙min^−1^) chromatography of Fr. C2 (224.4 mg) afforded compounds **9** (*t*_R_ = 82.0 min, 4.2 mg), **10** (*t*_R_ = 67.0 min, 7.2 mg), and Fr. C2-1 (134.3 mg), which was purified by semi-preparative HPLC (MeOH/H_2_O, 52%, 248 nm, 3 mL∙min^−1^) to afford compounds **16** (*t*_R_ = 20.0 min, 6.0 mg), **11** (*t*_R_ = 54.1 min, 4.7 mg), **12** (*t*_R_ = 54.2 min, 1.0 mg), and **17** (*t*_R_ = 84.0 min, 50.1 mg). Separation of Fr. C3 (3.0 g) by Sephadex LH-20 (MeOH) afforded compound **15** (36.2 mg). ODS chromatography (MeOH/H_2_O, 5–50%) of Fr. C4 yielded compound **18** (10.0 mg). Finally, Fr. D (3.2 g) was processed through Sephadex LH-20 (MeOH) and then repeatedly recrystallized from dichloroethane to obtain compound **19** (30.0 mg).

Uncahirsutine A (**1**): Whitish amorphous solid form; [*α*] −14 (*c* 1.0 in MeOH); ECD (MeOH) *λ* (Δ*ε*) 217 (−11.64), 227 (+3.54), 240 (−5.40) nm; HRESIMS *m*/*z* 343.2023 [M + H]^+^ (C_20_H_27_N_2_O_3_, 343.2022); UV (MeOH) *λ*_max_ (log *ε*) 252 (3.51), 208 (4.11) nm; NMR assignments are provided in Table 1 and Table 2.

Uncahirsutine B (**2**): Whitish amorphous solid form; [*α*] +112 (*c* 1.0 in MeOH); ECD (MeOH) *λ* (Δ*ε*) 210 (−0.98), 223 (+2.68), 234 (−1.62), 247 (+0.40) nm; HRESIMS *m*/*z* 343.2023 [M + H]^+^ (C_20_H_27_N_2_O_3_, 343.2022); UV (MeOH) *λ*_max_ (log *ε*) 252 (3.96), 213 (4.45) nm; NMR assignments are provided in Table 1 and Table 2.

Uncahirsutine C (**3**): Whitish amorphous solid form; [*α*] +158 (*c* 1.0 in MeOH); ECD (MeOH) *λ* (Δ*ε*) 208 (−0.31), 220 (−3.65), 230 (+16.80), 241 (−11.63), 254 (−4.13) nm; HRESIMS *m*/*z* 343.2025 [M + H]^+^ (C_20_H_27_N_2_O_3_, 343.2022); UV (MeOH) *λ*_max_ (log *ε*) 252 (3.79), 212 (4.29) nm; NMR assignments are provided in Table 1 and Table 2.

Uncahirsutine D (**4**): Whitish amorphous solid form; [*α*] +12 (*c* 1.0 in MeOH); ECD (MeOH) *λ* (Δ*ε*) 207 (−11.21), 225 (+10.01), 236 (+4.15), 240 (+4.07) nm; HRESIMS *m*/*z* 343.2027 [M + H]^+^ (C_20_H_27_N_2_O_3_, 343.2022); UV (MeOH) *λ*_max_ (log *ε*) 252 (3.63), 208 (4.23) nm; NMR assignments are provided in Table 1 and Table 2.

Uncahirsutine E (**5**): Whitish amorphous solid form; [*α*] +58 (*c* 1.0 in MeOH); ECD (MeOH) *λ* (Δ*ε*) 207 (−7.22), 216 (+22.67), 227 (−1.77), 240 (−3.67) nm; HRESIMS *m*/*z* 383.1605 [M + H]^+^ (C_21_H_23_N_2_O_5_, 383.1607); UV (MeOH) *λ*_max_ (log *ε*) 234 (4.09), 206 (4.43) nm; NMR assignments are provided in Table 1 and Table 2.

Uncahirsutine F (**6**): Whitish amorphous solid form; [*α*] +116 (*c* 1.0 in MeOH); ECD (MeOH) *λ* (Δ*ε*) 209 (+3.83), 214 (−0.53), 223 (+8.89), 242 (−9.51), 252 (−3.59), 257 (−3.96) nm; HRESIMS *m*/*z* 387.1917 [M + H]^+^ (C_21_H_27_N_2_O_5_, 387.1920); UV (MeOH) *λ*_max_ (log *ε*) 251 (3.92), 212 (4.42) nm; NMR assignments are provided in Table 1 and Table 2.

Uncahirsutine G (**7**): Whitish amorphous solid form; [*α*] +112 (*c* 1.0 in MeOH); ECD (MeOH) *λ* (Δ*ε*) 217 (−5.15), 228 (+17.35), 239 (−13.36), 249 (−4.55), 253 (−5.02) nm; HRESIMS *m*/*z* 401.2089 [M + H]^+^ (C_22_H_29_N_2_O_5_, 401.2076); UV (MeOH) *λ*_max_ (log *ε*) 251 (3.79), 209 (4.36) nm; NMR assignments are provided in Table 1 and Table 2.

Uncahirsutine H (**8**): Whitish amorphous solid form; [*α*] +44 (*c* 1.0 in MeOH); ECD (MeOH) *λ* (Δ*ε*) 207 (−3.89), 217 (+19.96), 228 (−14.51), 238 (−4.94), 242 (−5.04); HRESIMS *m*/*z* 401.2088 [M + H]^+^ (C_22_H_29_N_2_O_5_, 401.2076); UV (MeOH) *λ*_max_ (log *ε*) 251 (3.77), 209 (4.34) nm; NMR assignments are provided in Table 1 and Table 2.

Uncahirsutine I (**9**): Whitish amorphous solid form; [*α*] −144 (*c* 1.0 in MeOH); ECD (MeOH) *λ* (Δ*ε*) 219 (−9.82), 234 (+3.53), 244 (−1.90), 247 (+1.97) nm; HRESIMS *m*/*z* 401.2070 [M + H]^+^ (C_22_H_29_N_2_O_5_, 401.2076); UV (MeOH) *λ*_max_ (log *ε*) 252 (3.75), 210 (4.30) nm; NMR assignments are provided in Table 1 and Table 2.

Uncahirsutine J (**10**): Whitish amorphous solid form; [*α*] +440 (*c* 1.0 in MeOH); ECD (MeOH) *λ* (Δ*ε*) 203 (+7.99), 219 (−2.14), 231 (+14.43), 243 (−10.79), 253 (−3.44), 258 (−3.84) nm; HRESIMS *m*/*z* 401.2078 [M + H]^+^ (C_22_H_29_N_2_O_5_, 401.2076); UV (MeOH) *λ*_max_ (log *ε*) 251 (3.85), 211 (4.36) nm; NMR assignments are provided in Table 1 and Table 2.

Uncahirsutine K (**11**): Whitish amorphous solid form; [*α*] −124 (*c* 1.0 in MeOH); ECD (MeOH) *λ* (Δ*ε*) 206 (+1.78), 218 (−2.21), 232 (+9.02), 244 (−7.44), 252 (−3.25), 259 (−4.37) nm; HRESIMS *m*/*z* 401.2082 [M + H]^+^ (C_22_H_29_N_2_O_5_, 401.2076); UV (MeOH) *λ*_max_ (log *ε*) 251 (3.95), 212 (4.43) nm; NMR assignments are provided in Table 1 and Table 2.

Uncahirsutine L (**12**): Whitish amorphous solid form; [*α*] +62 (*c* 1.0 in MeOH); ECD (MeOH) *λ* (Δ*ε*) 209 (−2.26), 220 (−5.39), 232 (+4.73), 245 (+1.58) nm; HRESIMS *m*/*z* 401.2078 [M + H]^+^ (C_22_H_29_N_2_O_5_, 401.2076); UV (MeOH) *λ*_max_ (log *ε*) 251 (3.88), 211 (4.37) nm; NMR assignments are provided in Table 1 and Table 2.

### 3.4. Quantum Chemical Calculation

Energy minimization was initially performed using Chem3D 22.0, followed by conformational analysis with the MM2 force field implemented in SYBYL-X 2.0 software. Subsequent quantum mechanical calculations were conducted using the Gaussian 09 package. On the basis of the ECD results, conformations exhibiting high similarity to the experimental CD spectra were selected for density functional theory (DFT) calculations at the B3LYP/6-31G (d, p) level.

### 3.5. AChE Inhibitory Activity Assay

Sample wells: Briefly, 20 μL of sample solution, 140 μL of PBS buffer (0.1 M, pH = 8.0), and 20 μL of enzyme solution were added to the wells of the plate, mixed well, and stored at 37 °C for 15 min. Then, the plate was removed, and 10 μL of DTNB (2 mM) and 10 μL of ATCI (15 mM) were added, after which the mixture was allowed to react at 37 °C again. After 15 min, the absorbance value was read at 412 nm.

In the sample background control group, 20 μL of PBS was used instead of 20 μL of enzyme solution, and the other conditions remained unchanged.

In the blank control group, 20 μL of PBS was used instead of 20 μL of the sample solution to be tested, and the other conditions remained unchanged.

In the blank background control group, 20 μL of sample solution and 20 μL of enzyme solution were replaced with 40 μL of PBS, and the other conditions remained unchanged.

For samples with significant acetylcholinesterase inhibitory activity at an initial concentration of 1 mg/mL, the half-inhibitory concentration (IC_50_) was further determined by selecting an appropriate gradient, and the IC_50_ of huperzine A as a positive control drug against acetylcholinesterase was also determined. The IC_50_ values of the samples were determined by measuring the inhibition rate of acetylcholinesterase at seven different concentrations, after which the corresponding inhibition rates at different concentrations were entered into the IC_50_ calculator to obtain the IC_50_ values of the samples.

### 3.6. Cell Evaluation

#### 3.6.1. Chemicals and Reagents

All isolated compounds (**1**–**19**) were dissolved in dimethyl sulfoxide (DMSO) to prepare stock solutions, which were stored at −20 °C. The final concentration of DMSO in all assay media was maintained below 0.1% (*v*/*v*) to ensure no detectable cytotoxicity. L-Glutamate was supplied by Yuanye Biotech Co., Ltd. (Shanghai, China). The Cell Counting Kit-8 (CCK-8) and 96-well cell culture plates were supplied by Labgic Technology Co., Ltd. (Beijing, China). The Reactive Oxygen Species Assay Kit was supplied by Solarbio Technology Co., Ltd. (Beijing, China).

#### 3.6.2. Cell Culture

The mouse hippocampal neuronal cell line HT22 (CL-0697) (Wuhan Pricella Biotechnology Co., Ltd., Wuhan, China) was used throughout this study. Cells were cultured in Dulbecco’s Modified Eagle Medium (DMEM) supplemented with 10% FBS and 1% penicillin–streptomycin solution. They were maintained in a humidified incubator at 37 °C with 5% CO_2_. Experiments were conducted with cells in the logarithmic growth phase.

#### 3.6.3. Cytotoxicity Assay

HT22 cells were seeded into 96-well plates at a density of 1 × 10^4^ cells per well and allowed to adhere for 24 h. Subsequently, the culture medium was replaced with fresh medium containing the test compounds at a final concentration of 40 μM. After 24 h of incubation, cell viability was assessed using the CCK-8 assay according to the manufacturer’s instructions. The optical density (OD) at 450 nm was measured using a Cytation 5 multifunctional full-wavelength microplate reader (BioTek, Winooski, VT, USA).

#### 3.6.4. Neuroprotective Activity Assay

The neuroprotective effect against glutamate-induced excitotoxicity was investigated. HT22 cells were seeded as described in Section 3.6.3. After cell attachment, they were divided into the following treatment groups for 24 h: (1) Control group (normal medium); (2) Model group (treated with 24 mM L-glutamate alone); (3) Treatment groups (co-treated with 24 mM L-glutamate and test compounds at 40 μM). Following the treatment period, cell viability was quantified using the CCK-8 assay. The neuroprotective effect of each compound was calculated based on the percentage increase in cell viability compared to the model group.

#### 3.6.5. Intracellular ROS Measurement Assay

ROS levels were detected using the fluorescent probe DCFH-DA. Briefly, HT22 cells were seeded into 24-well plates at a density of 3000 cells per well and cultured for 24 h to allow attachment. The cells were then divided into three groups and treated as follows for 24 h: (1) Control group (normal medium); (2) Model group (treated with 24 mM L-glutamate alone); (3) Treatment groups (co-treated with 24 mM L-glutamate and test compounds at 40 μM). After treatment, the culture medium was removed, and each well was washed once with PBS. Subsequently, 500 μL of a 2 μM DCFH-DA solution (dissolved in serum-free DMEM) was added to each well, followed by incubation at 37 °C for 30 min in the dark. The DCFH-DA solution was then carefully aspirated, and cells were washed twice with 500 μL PBS to remove residual probe. Finally, 500 μL of PBS was added to each well, and fluorescence images were captured immediately under a fluorescence microscope (Olympus, Tokyo, Japan). The experiment was performed under light-protected conditions throughout the procedure.

### 3.7. Statistical Analysis

All experiments were performed in triplicate, with four replicates per experimental group (*n* = 4), and the results are shown as the mean values ± standard deviations (SD). Statistical analyses were performed using GraphPad Prism 8 (GraphPad, San Diego, CA, USA). *p* < 0.05 was considered to indicate statistical significance.

## 4. Conclusions

Previous phytochemical investigations of the genus *Uncaria* have led to the isolation and structural elucidation of multiple alkaloid subclasses, including tetracyclic, pentacyclic, and cadambine monomeric indole alkaloids, and their dimeric derivatives, which exhibit complex stereochemistry and intriguing biological properties [28,29,30,31]. In this study, twelve previously undescribed monoterpene indole alkaloids (**1**–**12**) were isolated from *U. hirsuta*. Notably, the structural profile of *U. hirsuta* alkaloids displays distinct characteristics compared to other *Uncaria* species such as *U. rhynchophylla* and *U. macrophylla*. Although the latter are predominantly characterized by tetracyclic oxindole alkaloids (e.g., RN and IRN) and their dimers, the compounds isolated from *U. hirsuta* are mainly pentacyclic monomers with higher degrees of oxygenation, particularly at C-17. Classical tetracyclic oxindoles such as RN and IRN typically bear an ester group at C-16. In contrast, several of the new compounds described herein feature advanced oxidation states at these positions, including carbonyl groups at C-5 (e.g., compound **5**) and hydroxyl or hydroxymethyl groups at C-17 (e.g., compounds **1** and **6**). Moreover, the pentacyclic core observed in these compounds represents a significant expansion of the typical tetracyclic scaffold found in most *Uncaria* alkaloids.

The systematic evaluation of the isolated compounds (**1**–**19**) revealed their cytotoxicity and neuroprotective effects in the glutamate-induced HT22 cell injury model. While most of the compounds were found to be non-cytotoxic at 40 μM, compounds **1** and **4** were identified as exceptions, demonstrating significant toxicity. More importantly, the neuroprotective screening highlighted six active compounds (**5**, **10**, **12**, **16**, **18**, and **19**) that significantly attenuated glutamate-induced neuronal death. Further investigation using the DCFH-DA fluorescent probe indicated that the neuroprotective effects of these active compounds were associated with a significant reduction in intracellular ROS levels, suggesting that their mechanism of action may involve the mitigation of glutamate-induced oxidative stress. These findings collectively position compounds **5**, **10**, **12**, **16**, **18**, and **19** as promising lead candidates for further investigation. The weak effect of some alkaloids on both neuroprotective activity in the glutamate-induced HT22 cell and AChE inhibition may be partly due to their lower purity (e.g., compounds **4**, **6**–**8**). To sum up, this study not only expands the phytochemical diversity of *Uncaria* species but also provides valuable insights for developing multi-target neuroprotective agents.

## Figures and Tables

**Table 1:** ^1^H NMR data of compounds **1**–**12**.

No.	1 ^a^	2 ^b^	3 ^b^	4 ^c^	5 ^d^	6 ^b^	7 ^b^	8 ^b^	9 ^b^	10 ^b^	11 ^b^	12 ^b^
3	2.28, dd (9.1, 4.1)	2.29, dd (9.5, 4.1)	2.45, m	2.24, dd (11.1, 2.7)	3.93, dd, (11.9, 3.7)	2.52, overlap	2.56, m	2.49, m	2.48, m	2.48, m	2.57, dd (11.1, 2.5)	2.38, dd (11.2, 2.2)
5	2.48, m	2.46, overlap	2.48, m	2.04, overlap		2.52, overlap	2.50, m	2.48, m	2.48, m	2.50, m	2.51, t (8.7)	2.49, m
	3.40, m	3.39, m	3.29, m	2.47, overlap		3.28, m	3.27, m	1.80, m	3.28, m	3.29, td (8.5, 2.0)	3.27, dd (8.6, 2.0)	3.59, m
6	2.04, m	2.01, m	2.02, m	1.05, m	2.96, d (17.3)	1.97, m	2.36, m	2.01, m	2.02, m	2.00, dd (21.1, 8.4)	2.00, m	2.00, m
	2.47, m	2.46, overlap	2.37, m	2.47, overlap	2.46, d (16.8)	2.38, m	2.00, m	2.34, m	2.27, m	2.38, td (10.0, 2.1)	2.36, m	2.46, m
9	7.20, overlap	7.18, overlap	7.38, d (7.4)	7.20, overlap	7.15, d (7.4)	7.36, d (7.5)	7.33, d (7.3)	7.35, d (7.3)	7.18, m	7.36, d (7.4)	7.35, d (7.4)	7.18, m
10	7.05, t (7.7)	7.03, t (7.5)	7.04, t (7.6)	7.05, t (7.6)	7.01, t (7.5)	7.04, m	6.99, t (7.5)	7.01, t (7.4)	7.04, m	7.04, t (7.5)	7.01, t (7.2)	7.04, d (7.2)
11	7.20, overlap	7.18, overlap	7.19, t (7.7)	7.20. overlap	7.23, t (7.7)	7.20, m	7.15, t (7.5)	7.18, t (7.7)	7.18, m	7.20, t (7.7)	7.18, td (7.7, 1.1)	7.18, m
12	6.85, d (7.9)	6.87, d (7.7)	6.87, d (7.8)	6.87, d (7.5)	6.92, d (7.8)	6.85, d (7.7)	6.88, d (7.7)	6.89, d (7.7)	6.84, d (7.4)	6.85, d (7.7)	6.83, d (7.7)	6.82, d (7.8)
14	1.21, m	1.21, m	0.72	1.33, overlap	2.16, dt (12.7, 3.3)	1.06, m	1.50, m	1.02, m	1.13, m	1.03, m	1.50, m	1.25, m
			1.22, m	1.43, m	0.70, q (12.1)	0.77, m	0.52, q (11.6)	0.80, m	0.86, m	0.76, dd (23.6, 11.7)	0.49, dd (23.2, 11.5)	
15	1.64, m	1.54, m	1.53, m	1.33, m	2.34, t (11.4)	1.59, m	1.89, m	1.57, m	1.61, d (11.8)	1.57, ddd (22.6, 11.3, 3.1)	1.90, dd (11.4, 3.2)	1.84, m
16	1.17, m	1.46, m	1.01, m	1.13, m		2.06, m	2.34, m	2.10, m	2.27, m	2.10, dd (11.4, 8.5)	2.33, m	3.37, brs
	1.20, m	1.57, m	1.61, m	1.67, m								
17	4.28, d (8.6)	4.67, d (2.6)	4.53, dd (9.6, 2,1)	4.52, dd (9.7, 2.4)	7.48, s	4.83, d (3.7)	4.80, d (3.8)	4.42, d (8.4)	4.42, d (8.4)	4.43, d (8.4)	4.81, d (3.8)	4.84, d (3.7)
18	1.23, d (6.2)	1.15, d (6.2)	1.17, d (7.0)	1.17, d (7.1)	1.34, d (6.2)	1.26, d (6.2)	1.17, d (6.3)	1.25, d (6.1)	1.25, d (6.0)	1.26, d (6.3)	1.18, d (6.3)	1.16, d (6.2)
19	3.23, m	3.56, m	4.19, m	4.19, m	3.84, m	3.44, m	3.62, m	3.37, m	3.37, m	3.37, dd (9.7, 6.1)	3.64, m	3.61, m
20	1.35, m	1.43, m	1.75, overlap	1.96, m	1.29, m	1.34, m	1.26, m	1.26, m	1.24, m	1.24, m	1.25, m	1.49, td (10.6, 3.5)
21	1.66, m	1.69, t (10.5)	1.77, overlap	1.67, overlap	4.19, dd (12.8, 4.2)	1.83, m	1.85, m	3.28, m	1.70, m	1.79, t (10.8)	1.85, t (10.8)	1.77, m
	3.31, m	3.26, dd (10.4, 3.5)	3.02, d (7.0)	3.12, dd (10.5, 3.5)	2.63, dd (14.0, 10.8)	3.18, m	3.16, m	3.18, m	3.17, m	3.18, dd (10.6, 3.6)	3.16, dd (10.5, 3.6)	3.25, dd (10.5, 3.8)
22	3.45, s	3.30, s	3.39, s	3.40, s								
22-OMe					3.53, s	3.61, s	3.56, s	3.41, s	3.58, s	3.59, s	3.58, s	3.59, s
17-OMe							3.27, s	3.56, s	3.44, s	3.43, s	3.29, s	3.29, s
NH	7.53			7.78, s		7.77, s	8.78, s	8.96, s	7.67, s	7.80, s	7.58, s	7.52, s

^a^ 600 MHz in CDCl_3_; ^b^ 400 MHz in CDCl_3_; ^c^ 700 MHz in CDCl_3_; ^d^ 400 MHz in CD_3_OD.

**Table 2:** ^13^C NMR data of compounds **1**–**12**.

No.	1 ^a^	2 ^b^	3 ^a^	4 ^a^	5 ^c^	6 ^b^	7 ^b^	8 ^b^	9 ^a^	10 ^b^	11 ^a^	12 ^a^
2	180.9	181.4	181.6	181.0	180.7	180.8	181.8	181.9	180.7	181.0	181.0	181.0
3	74.8	74.6	71.8	74.9	64.3	70.8	70.9	70.7	73.7	70.9	71.1	73.8
5	54.9	55.0	54.0	54.9	173.9	53.6	53.8	53.5	53.8	53.6	53.9	51.1
6	34.5	34.4	35.3	34.6	42.5	35.5	35.3	35.4	35.1	35.5	35.3	35.1
7	55.8	56.0	56.6	55.8	53.4	56.5	56.7	56.5	55.7	56.5	56.7	55.9
8	133.5	133.6	133.8	133.6	131.7	133.6	133.9	133.7	133.3	133.5	133.9	133.5
9	122.8	123.2	125.3	123.2	125.1	125.2	125.0	125.0	123.4	125.2	125.2	123.3
10	123.3	122.7	122.8	122.8	123.8	122.8	122.3	122.7	123.0	122.9	122.5	122.9
11	128.2	128.1	127.8	128.2	130.1	127.9	127.6	127.9	128.2	127.9	127.7	128.2
12	109.4	109.5	109.5	109.5	111.6	109.6	109.8	109.9	109.4	109.6	109.4	109.5
13	140.9	140.9	140.1	140.9	142.6	140.0	140.6	140.3	140.7	140.0	140.3	140.6
14	31.5	31.5	32.7	31.9	31.5	29.8	30.3	29.8	29.3	29.9	30.4	29.4
15	37.8	33.3	31.5	29.2	36.0	40.6	34.1	40.9	41.4	40.9	34.2	34.6
16	38.4	36.6	38.2	38.1	108.7	55.6	51.1	53.7	54.7	53.9	51.2	54.8
17	102.6	98.4	97.1	97.0	157.4	96.4	97.9	103.2	103.3	103.2	98.0	97.9
18	19.0	19.0	14.8	14.7	18.3	19.0	18.8	18.9	18.9	19.0	18.9	18.8
19	73.8	67.4	71.6	71.5	76.1	74.0	66.6	73.6	73.8	73.7	66.7	66.9
20	45.5	45.7	42.6	42.2	43.7	44.6	44.8	44.6	44.4	44.9	44.9	44.4
21	54.2	54.4	54.7	54.8	42.7	53.7	53.8	53.6	53.8	53.8	53.8	53.9
22	56.2	54.6	55.9	55.9	168.6	172.2	170.6	172.5	173.0	172.5	170.7	170.8
22-OMe					51.5	52.0	51.7	56.7	51.9	51.9	51.7	51.7
17-OMe							55.0	51.9	56.7	56.8	55.06	55.0

^a^ 150 MHz in CDCl_3_; ^b^ 100 MHz in CDCl_3_; ^c^ 100 MHz in CD_3_OD.

## Data Availability

All data generated or analyzed during this study are included in this published article and its Supplementary Information Files.

## Supplementary Materials

The following supporting information can be downloaded at: https://www.mdpi.com/article/10.3390/molecules31122053/s1.
