# Supplementary material for: New Monoterpenoid Indole Alkaloids as Potential Neuroprotective Agents from Uncaria hirsuta Haviland
Source: Molecules. 2026 Jun 11;31(12):2053. doi: 10.3390/molecules31122053 (PMC13304808; doi:10.3390/molecules31122053)
Supplement: Supplementary file 1 [file molecules-31-02053-s001.zip › molecules-4285684-supplementary.pdf]

List of isolated compounds with assigned numbers and full names

No.	Name
**1**	Uncahirsutine A
**2**	Uncahirsutine B
**3**	Uncahirsutine C
**4**	Uncahirsutine D
**5**	Uncahirsutine E
**6**	Uncahirsutine F
**7**	Uncahirsutine G
**8**	Uncahirsutine H
**9**	Uncahirsutine I
**10**	Uncahirsutine J
**11**	Uncahirsutine K
**12**	Uncahirsutine L

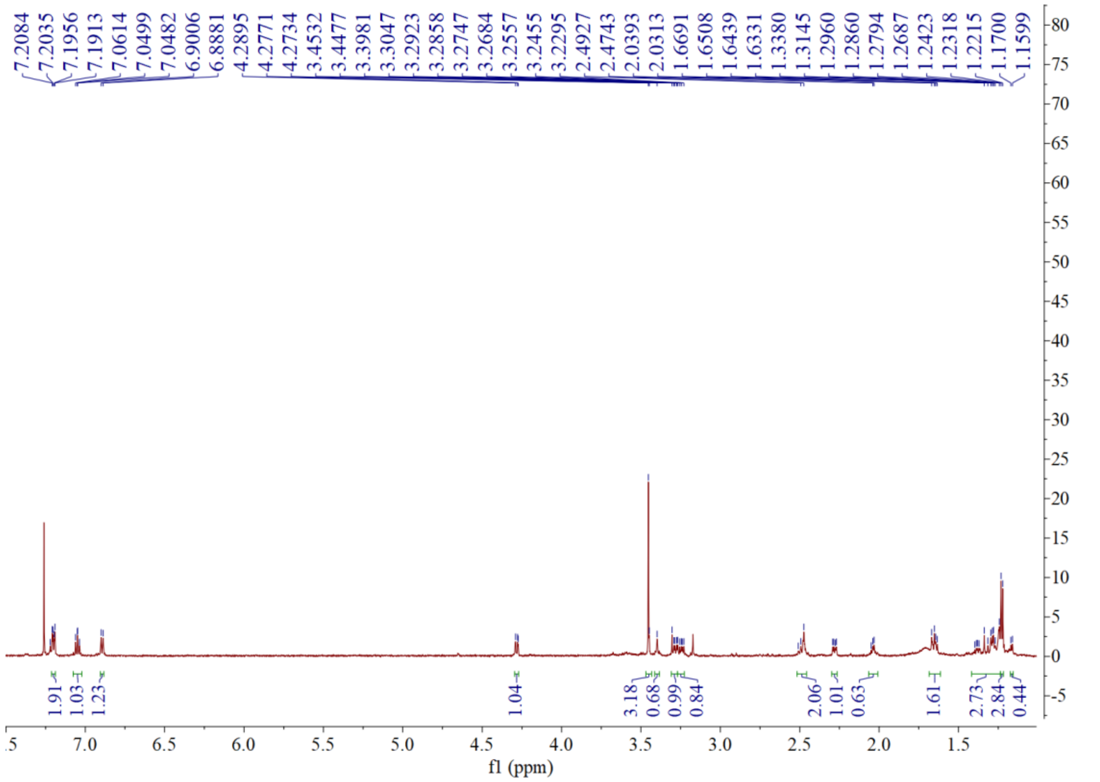

Figure S1. ^{1}H NMR spectra ($CDCl_3$, 600 MHz) of compound **1**

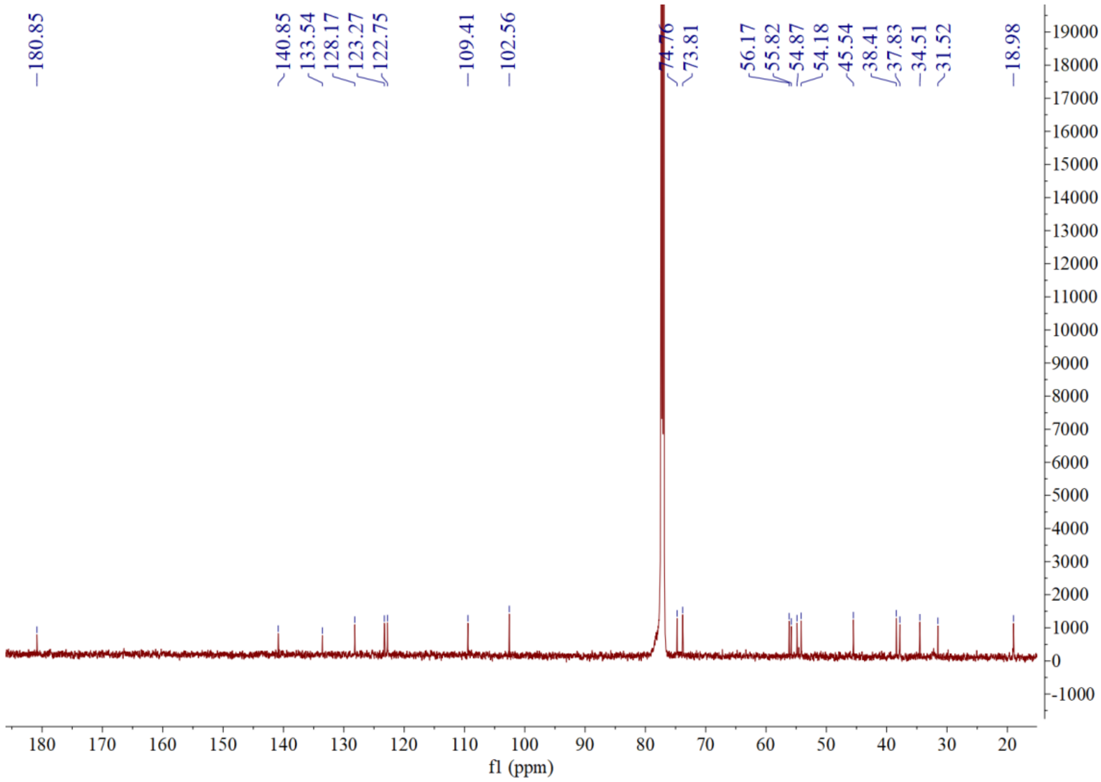

Figure S2. ^{13}C NMR spectra ($CDCl_3$, 150 MHz) of compound **1**

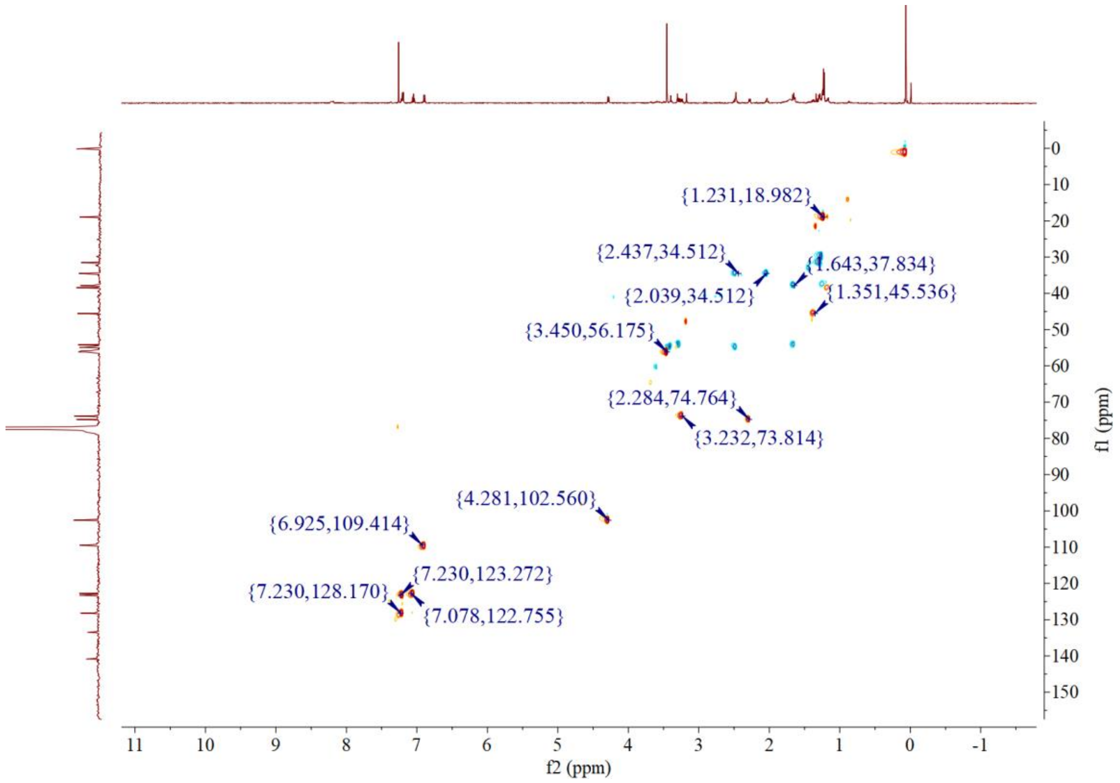

Figure S3. HSQC spectra ($CDCl_3$, 600 MHz) of compound **1**

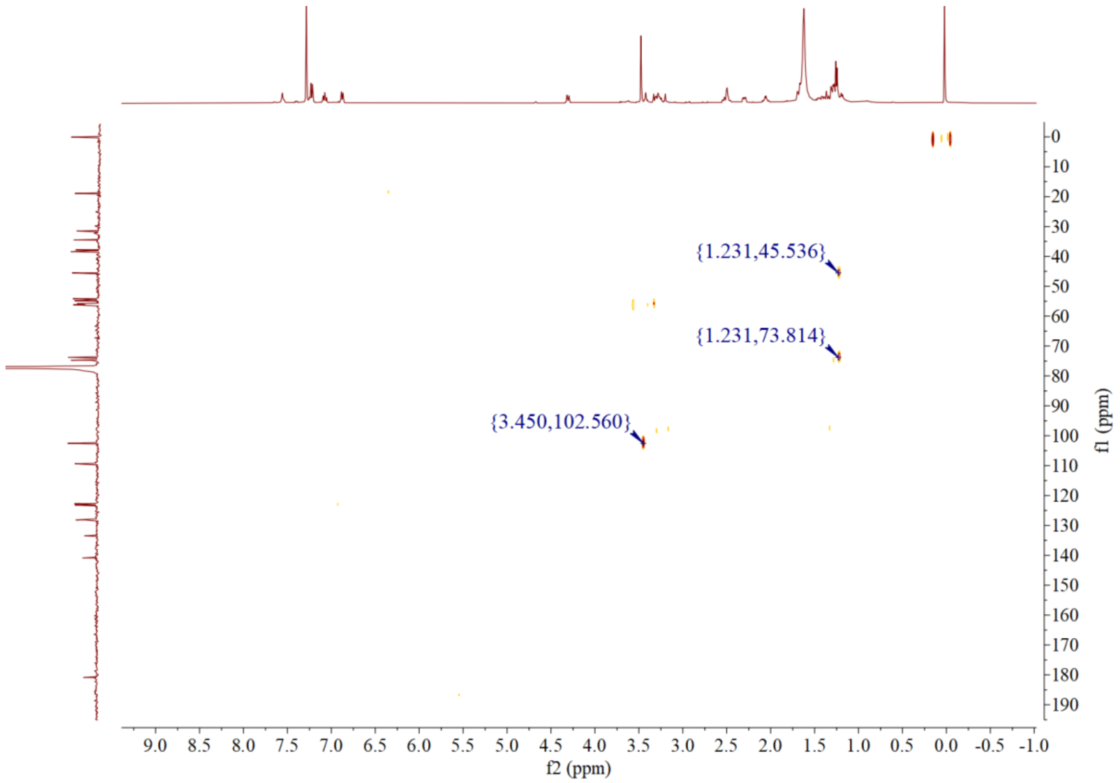

Figure S4. HMBC spectra ($CDCl_3$, 600MHz) of compound **1**

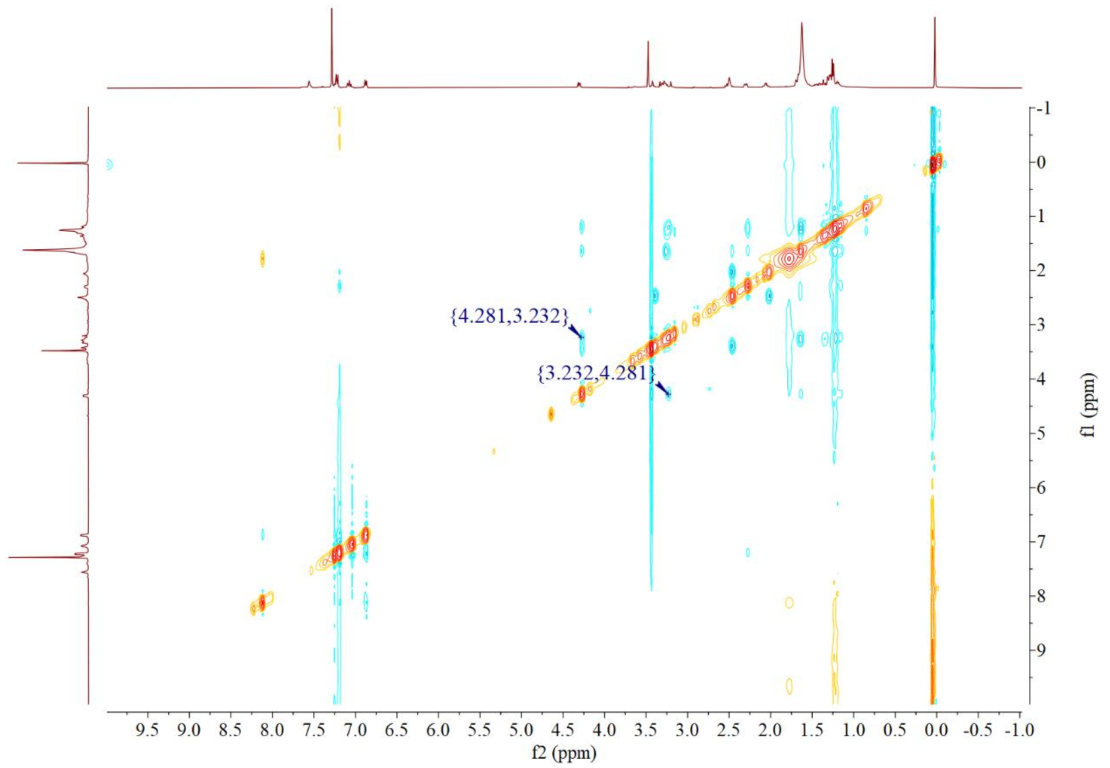

Figure S5. NOESY spectra ($CDCl_3$, 600MHz) of compound **1**

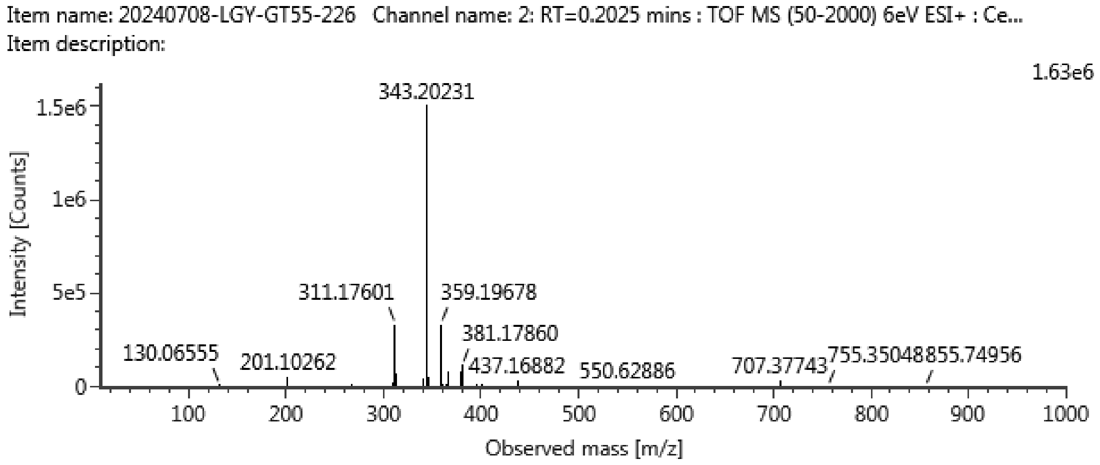

Figure S6. HRESIMS spectra of compound **1**

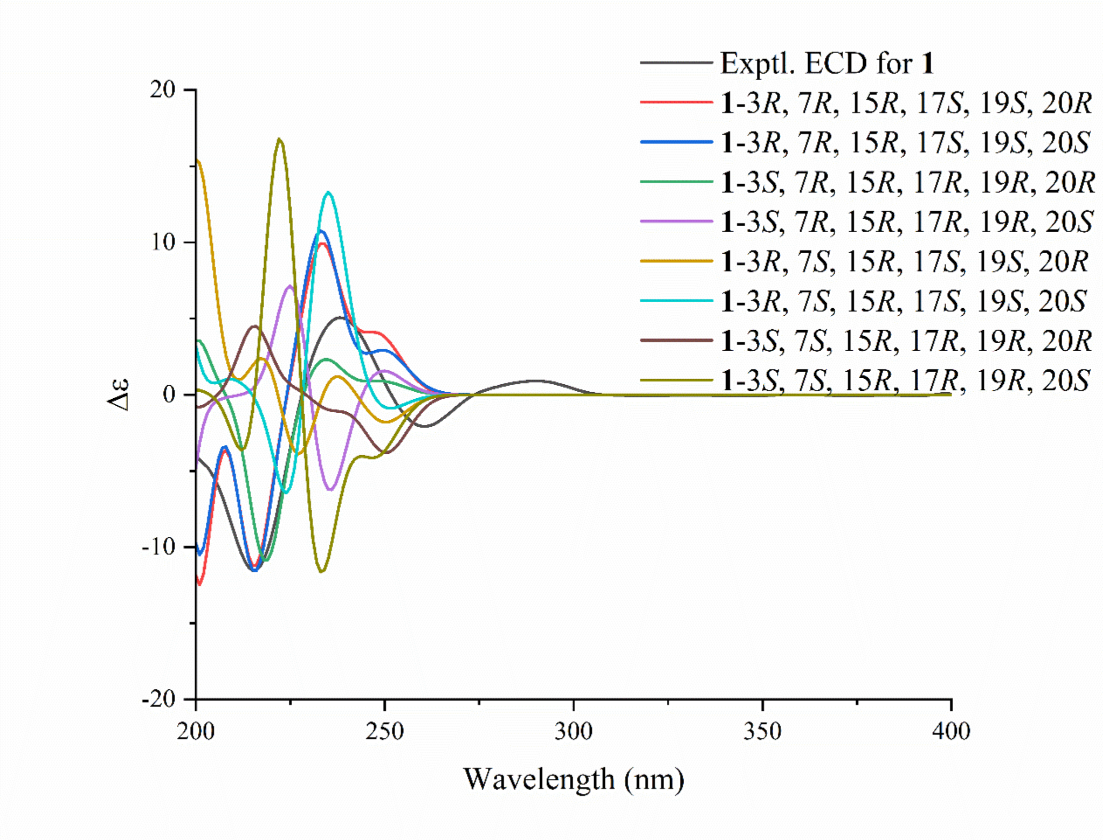

Figure S7. Experimental and calculated ECD spectra of compounds **1**

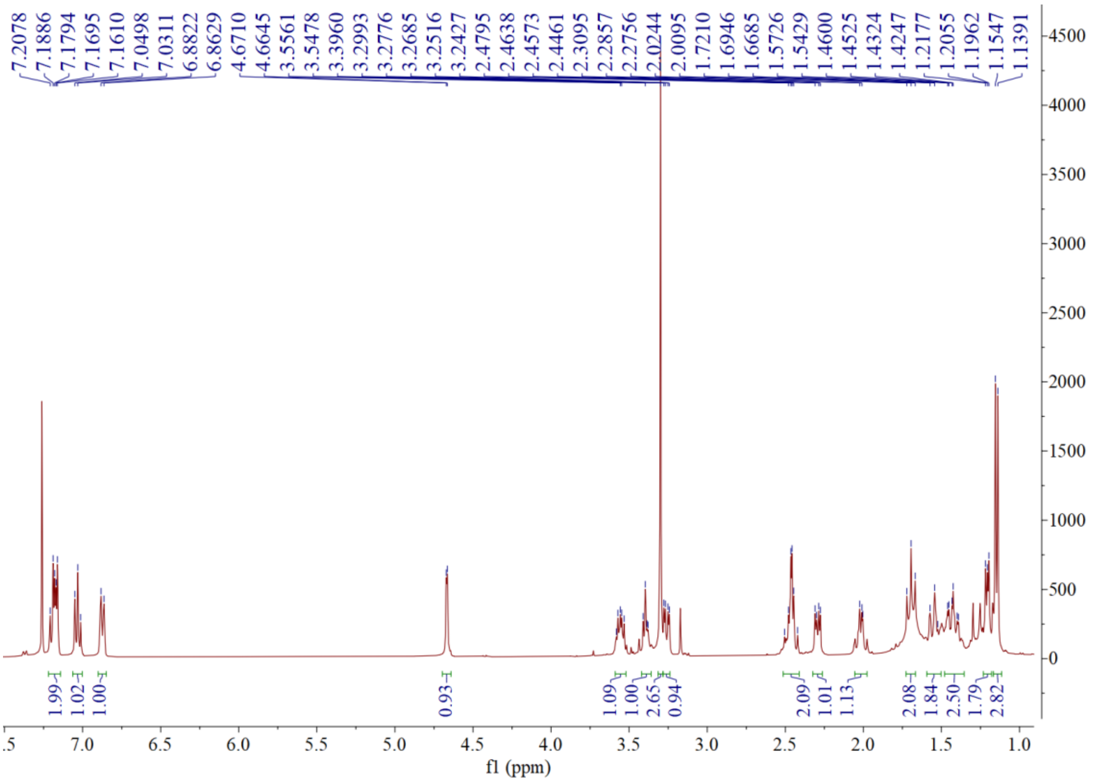

Figure S8. ^{1}H NMR spectra ($CDCl_3$, 400 MHz) of compound **2**

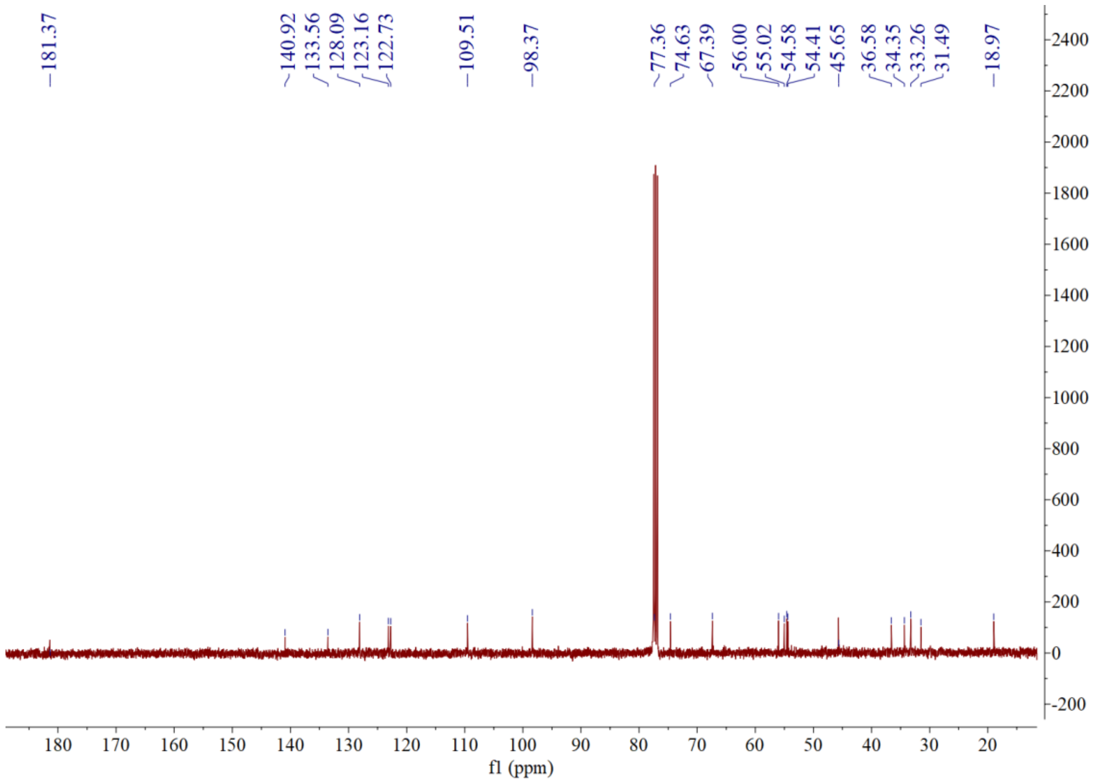

Figure S9. ^{13}C NMR spectra ($CDCl_3$, 100 MHz) of compound **2**

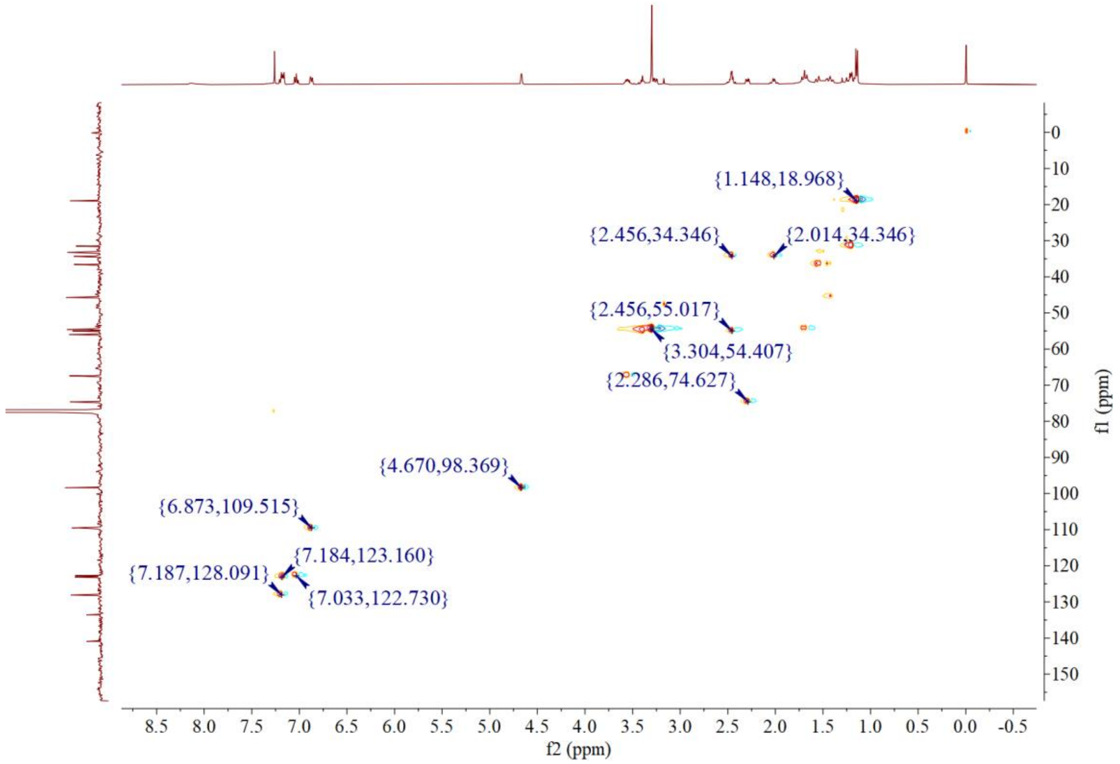

Figure S10. HSQC spectra ($CDCl_3$, 400 MHz) of compound **2**

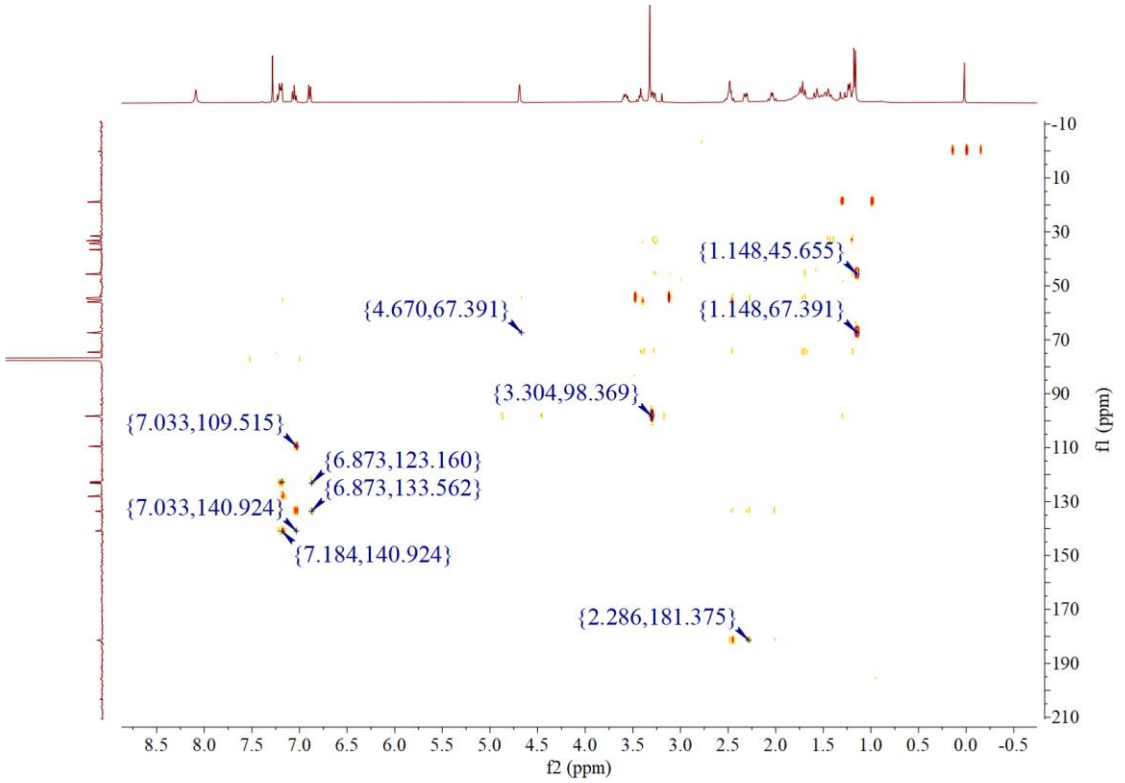

Figure S11. HMBC spectra ($CDCl_3$, 400 MHz) of compound **2**

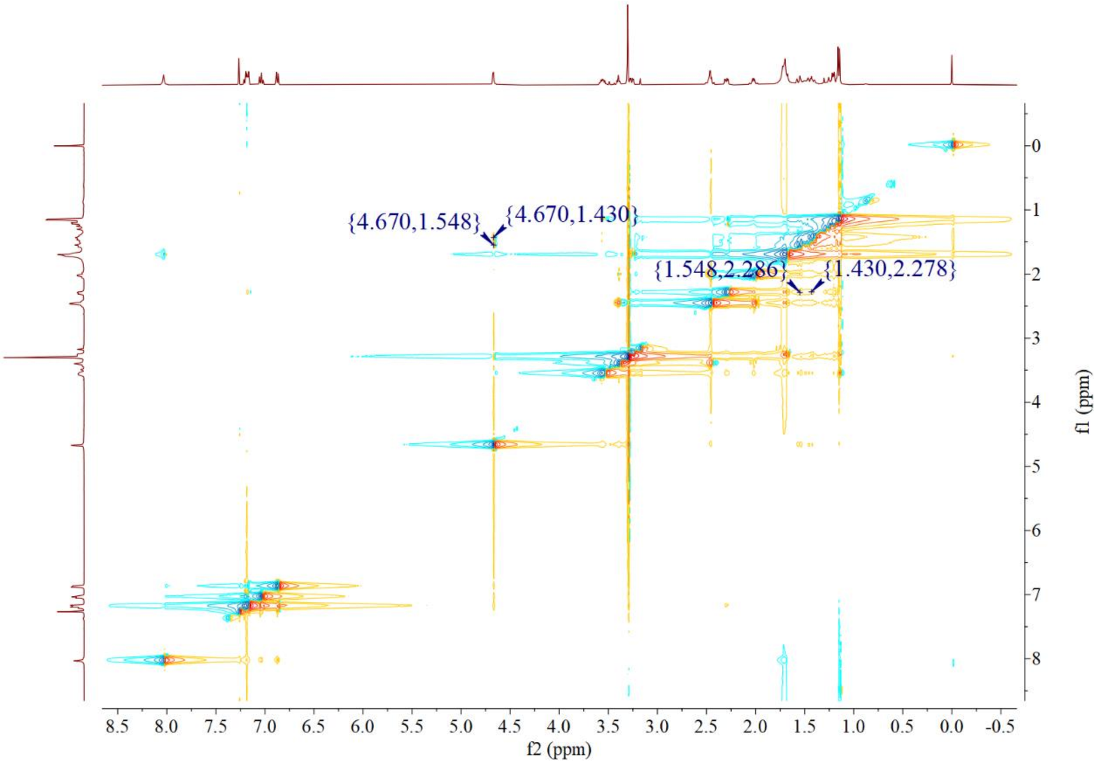

Figure S12. NOESY spectra ($CDCl_3$, 400 MHz) of compound **2**

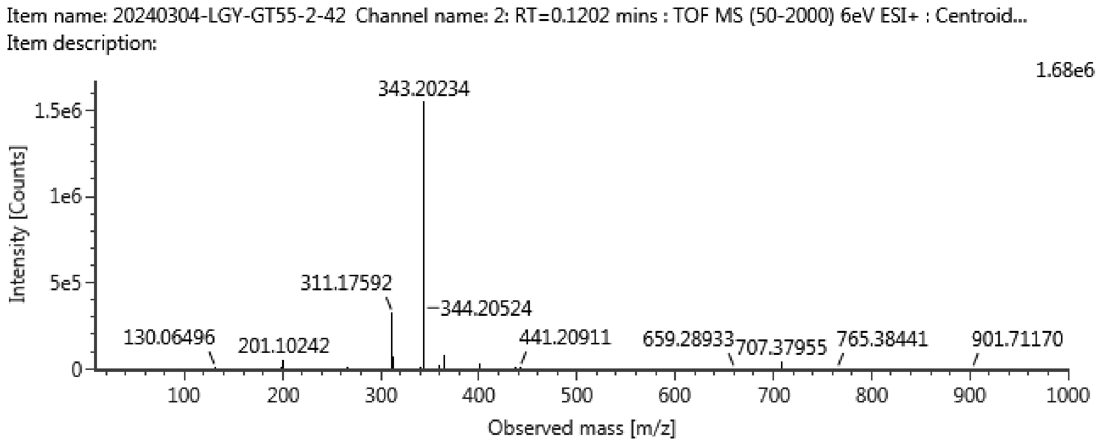

Figure S13. HRESIMS spectra of compound **2**

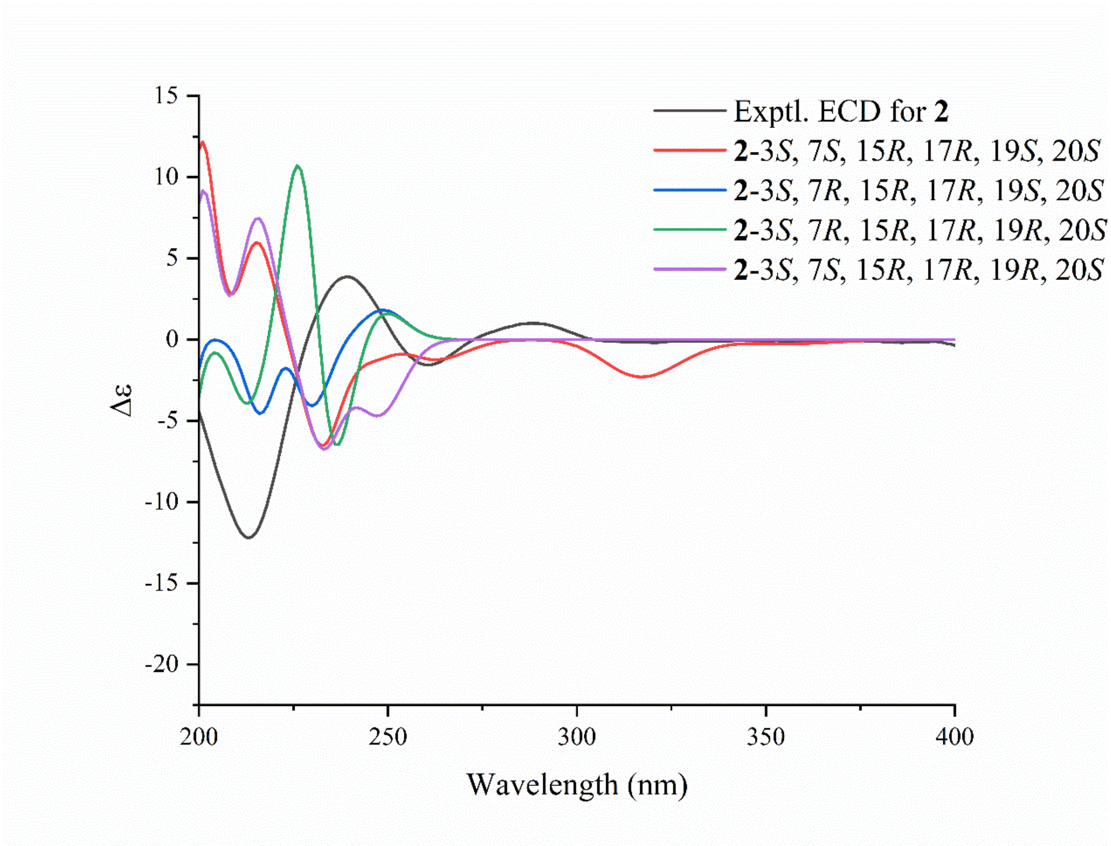

Figure S14. Experimental and calculated ECD spectra of compounds **2**

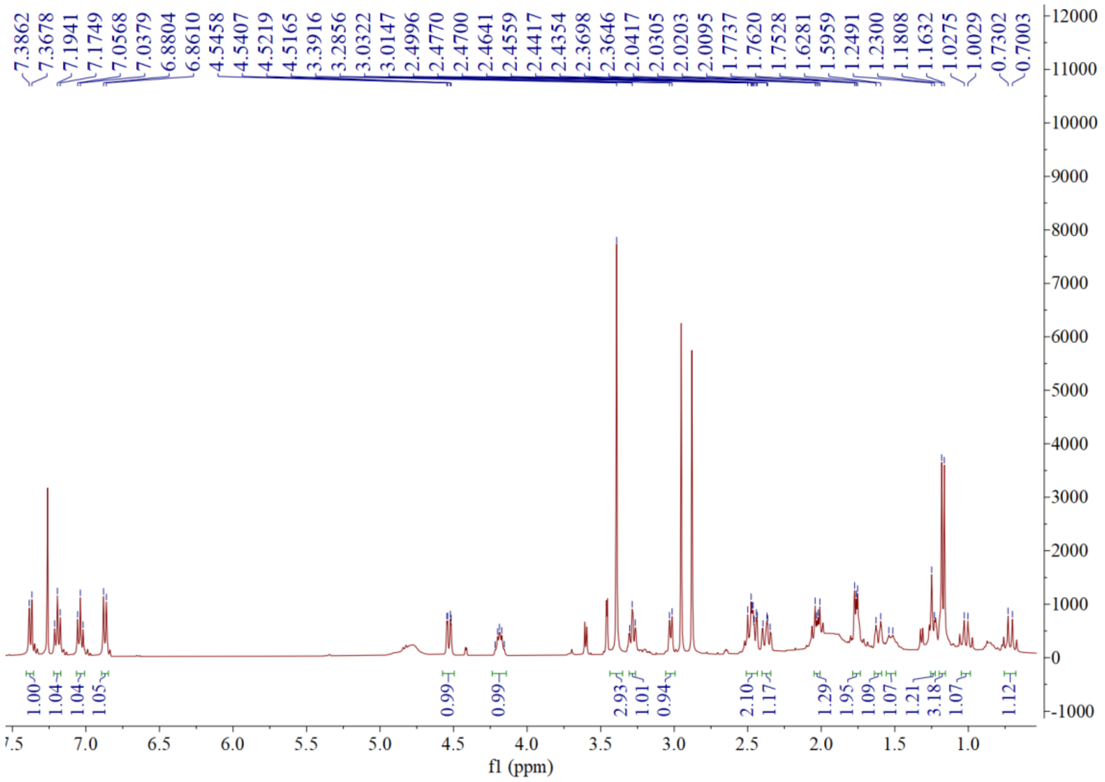

Figure S15. ^{1}H NMR spectra ($CDCl_3$, 400 MHz) of compound **3**

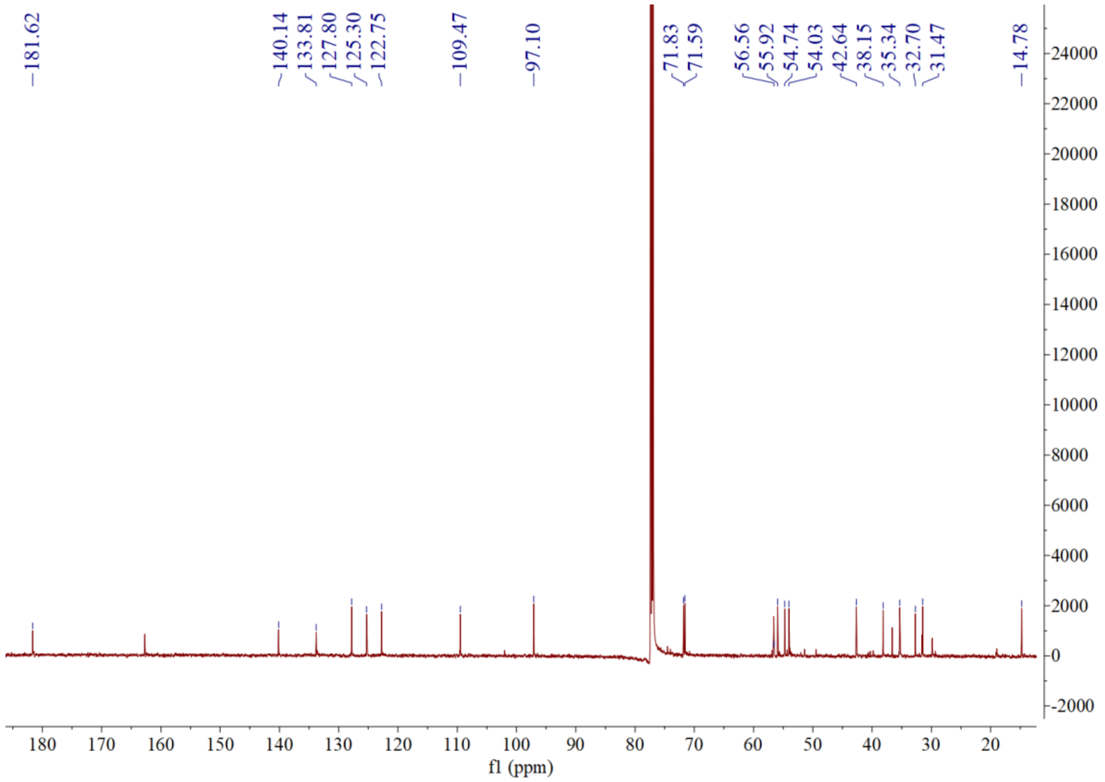

Figure S16. ^{13}C NMR spectra ($CDCl_3$, 150 MHz) of compound **3**

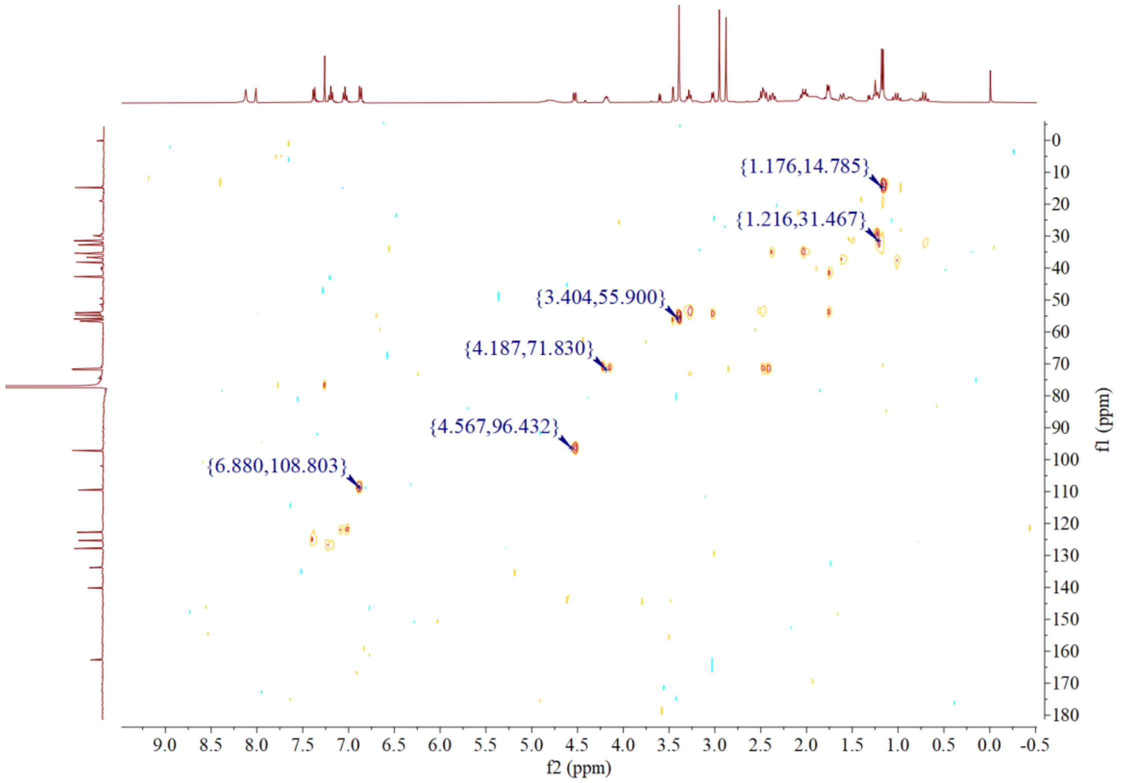

Figure S17. HSQC spectra ($CDCl_3$, 600 MHz) of compound **3**

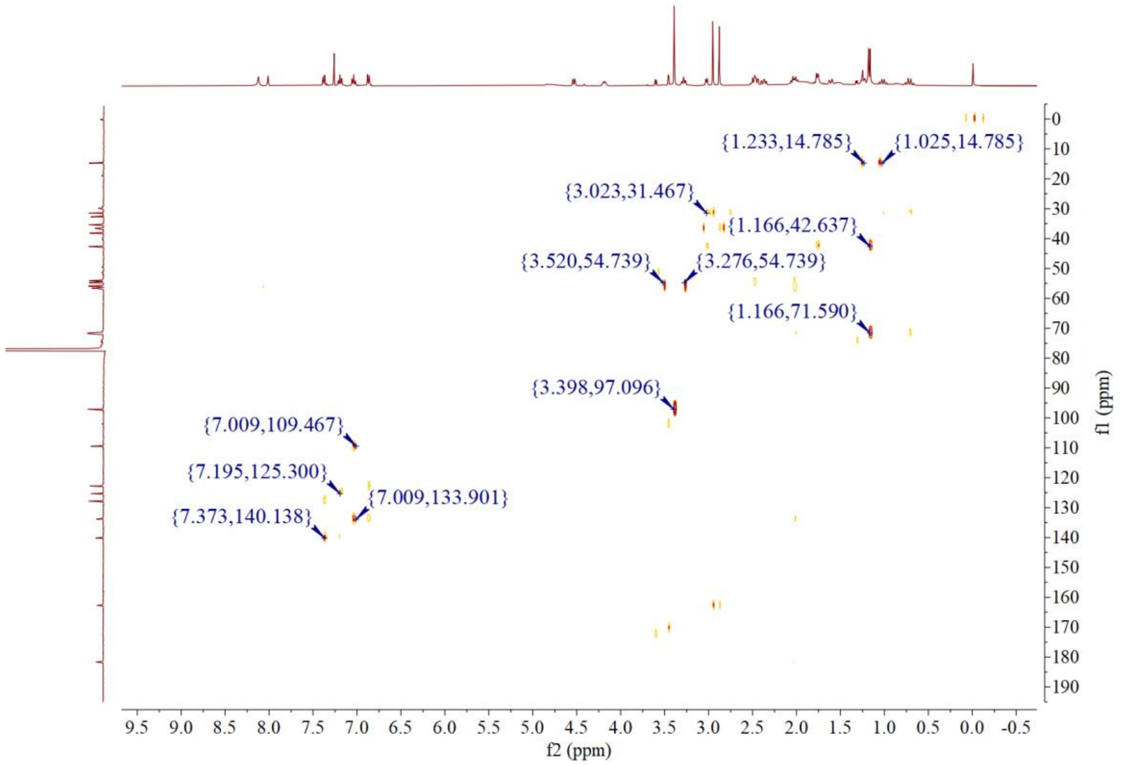

Figure S18. HMBC spectra ($CDCl_3$, 600 MHz) of compound **3**

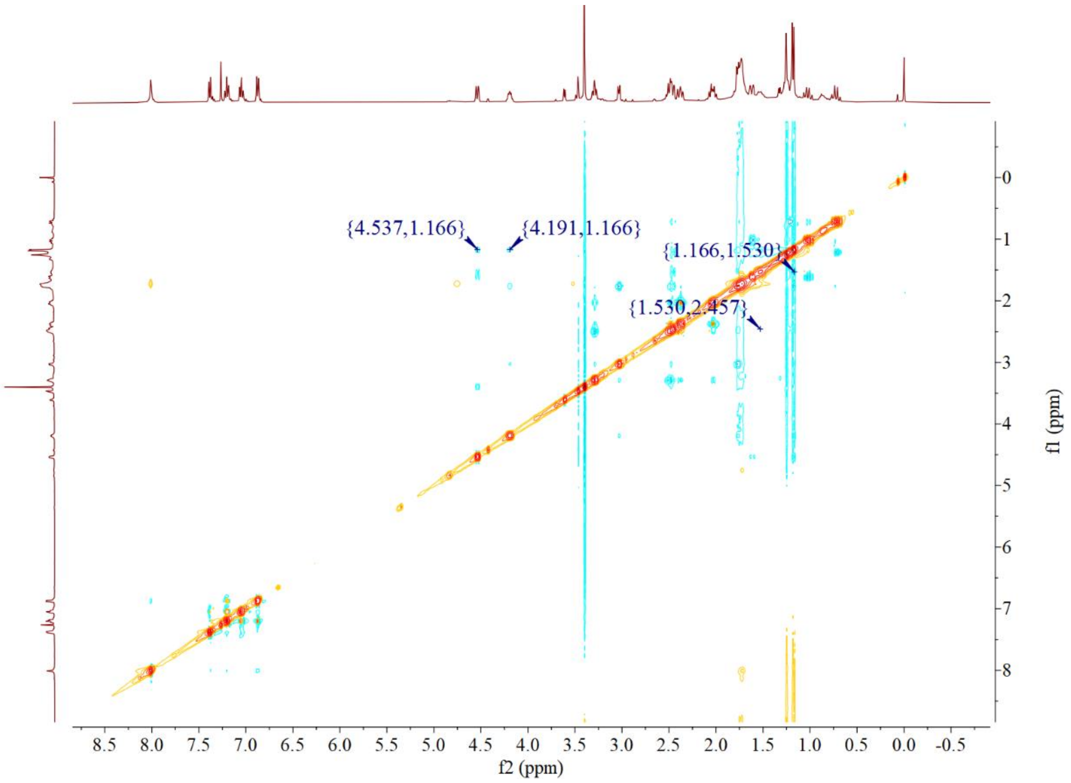

Figure S19. NOESY spectra ($CDCl_3$, 400 MHz) of compound **3**

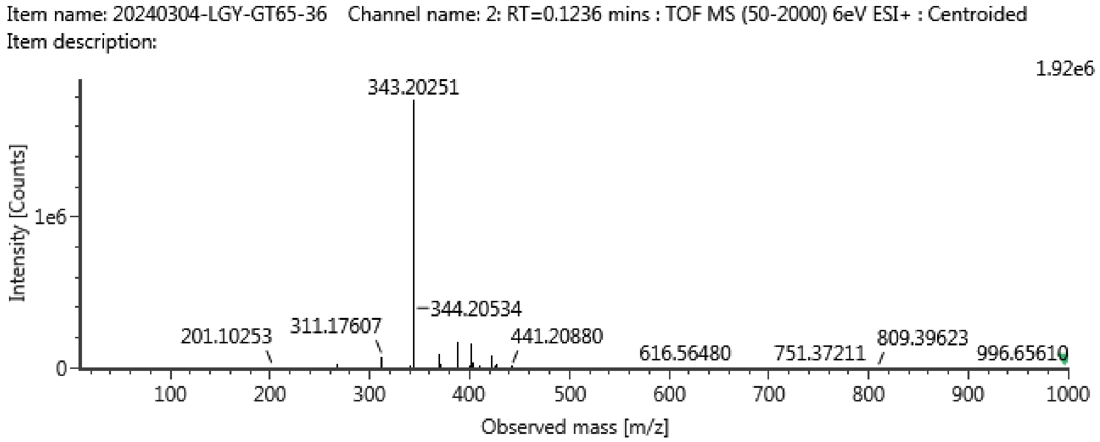

Figure S20. HRESIMS spectra of compound **3**

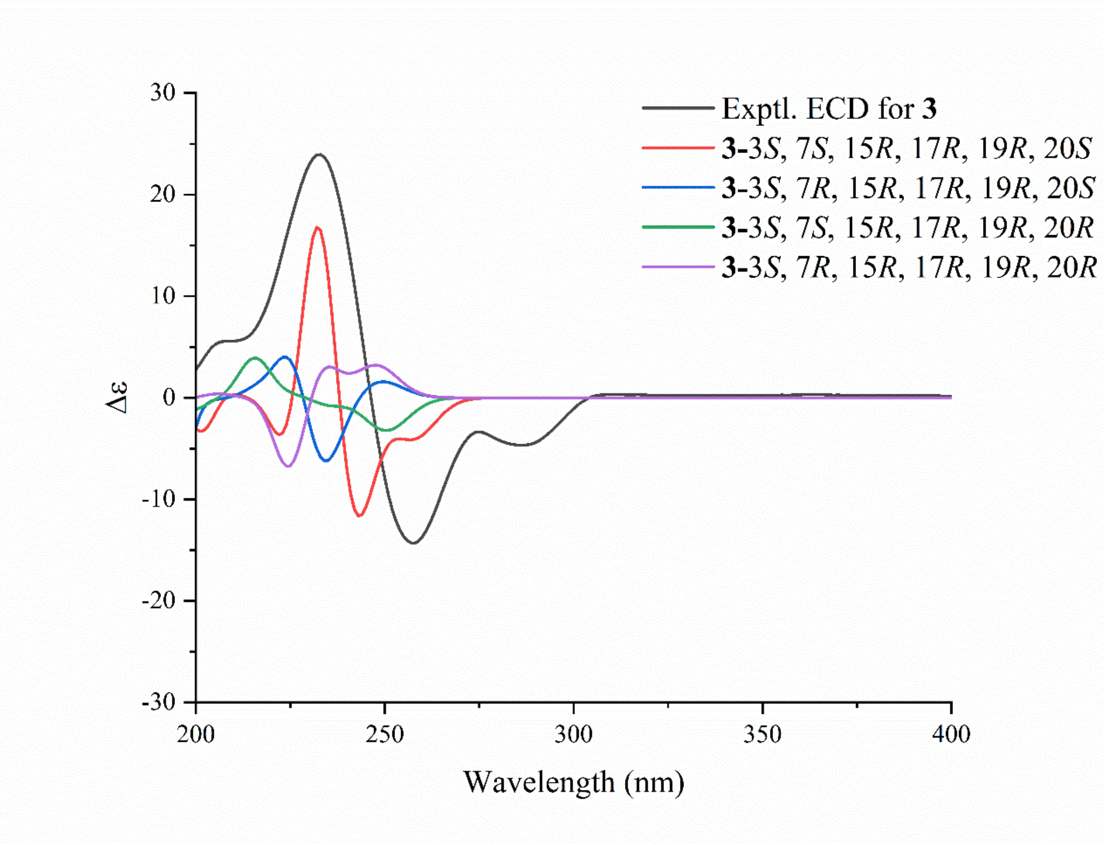

Figure S21. Experimental and calculated ECD spectra of compounds **3**

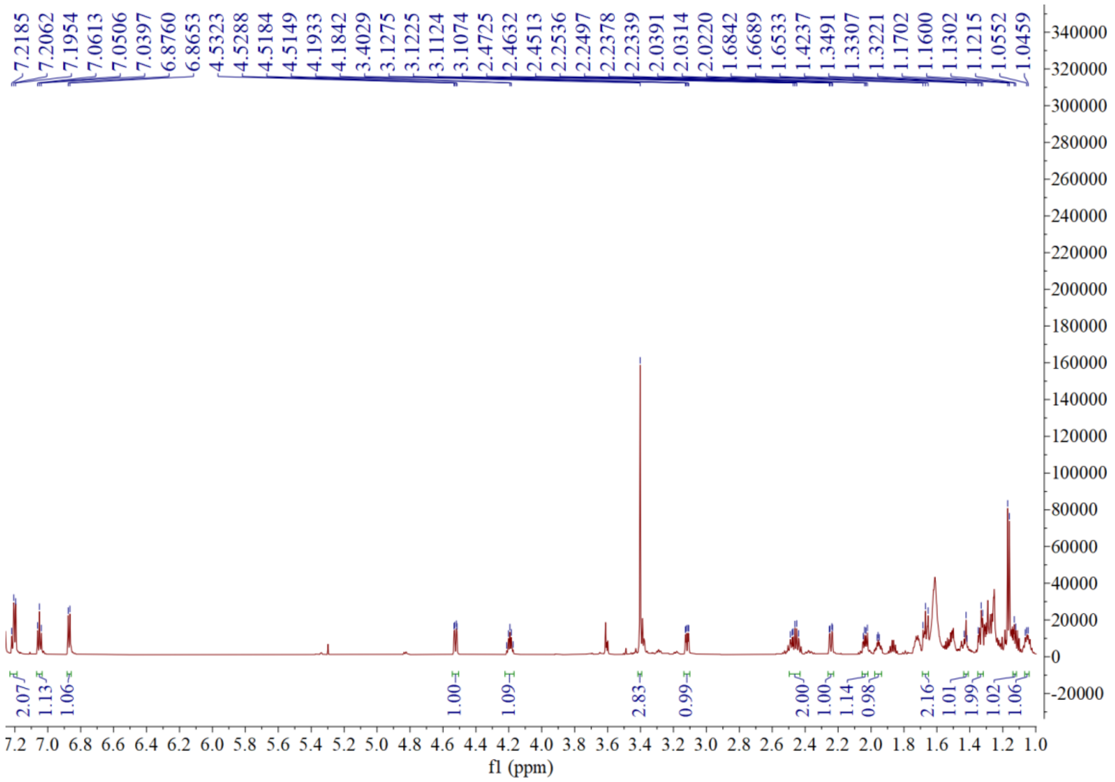

Figure S22. ^{1}H NMR spectra ($CDCl_3$, 700 MHz) of compound **4**

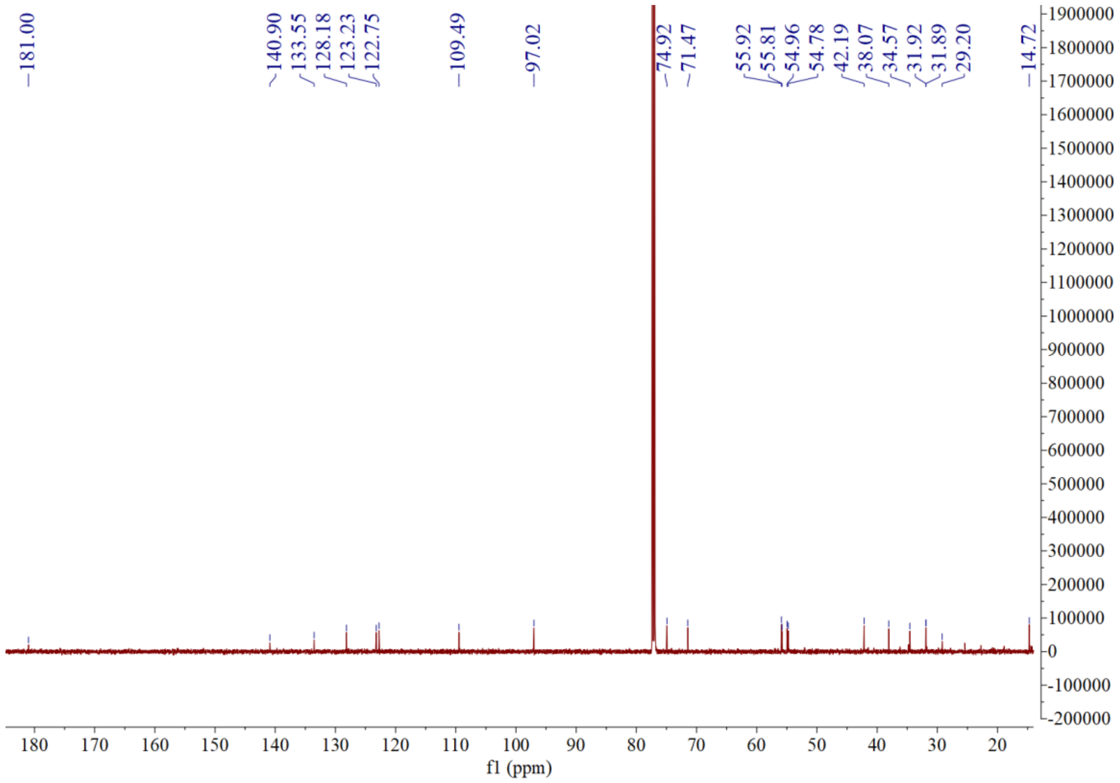

Figure S23. ^{13}C NMR spectra ($CDCl_3$, 150 MHz) of compound **4**

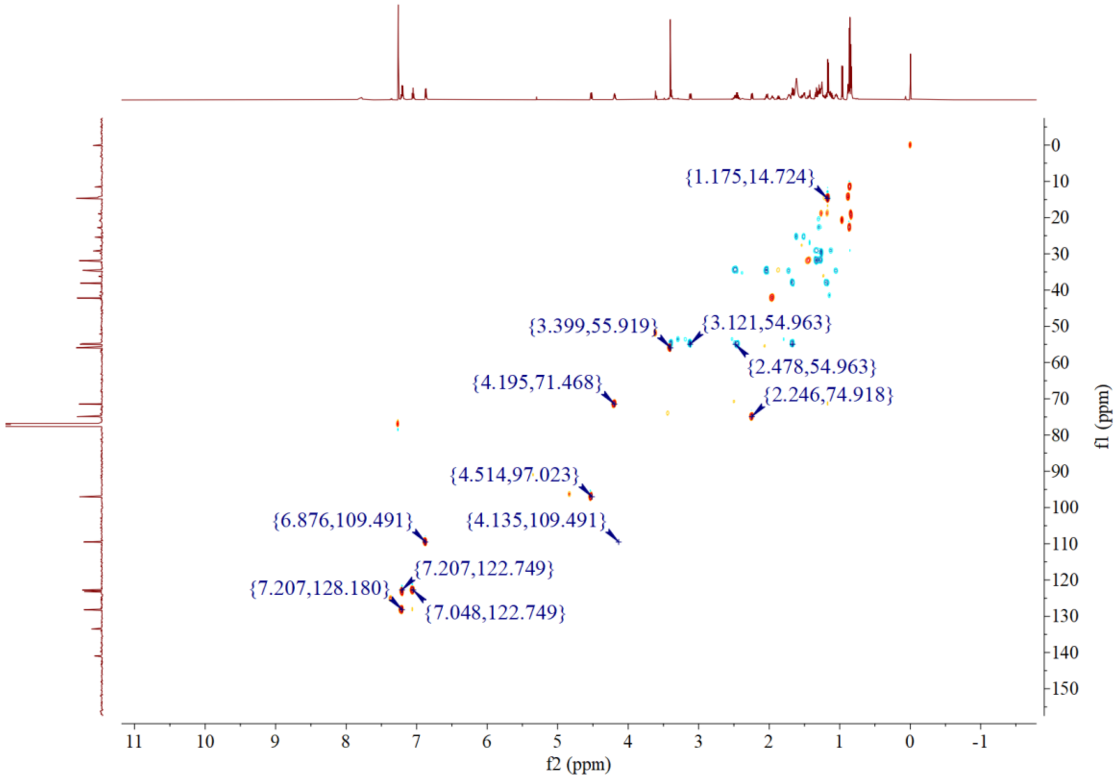

Figure S24. HSQC spectra ($CDCl_3$, 700 MHz) of compound **4**

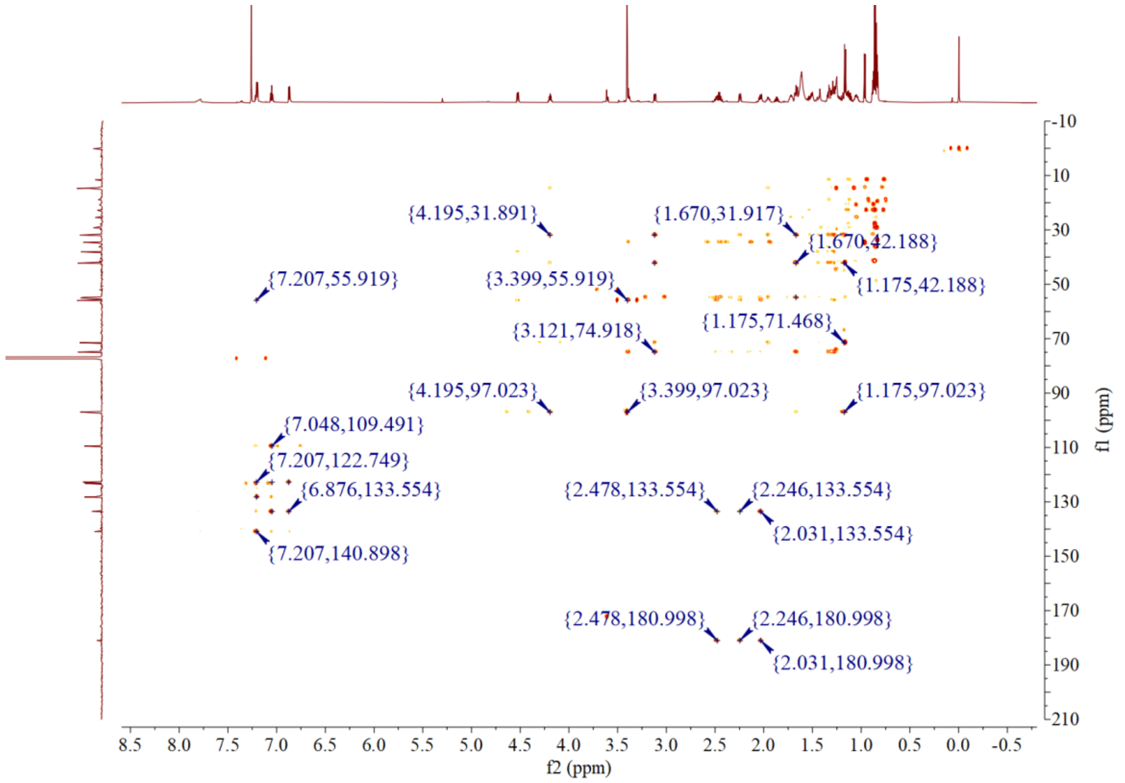

Figure S25. HMBC spectra ($CDCl_3$, 700 MHz) of compound **4**

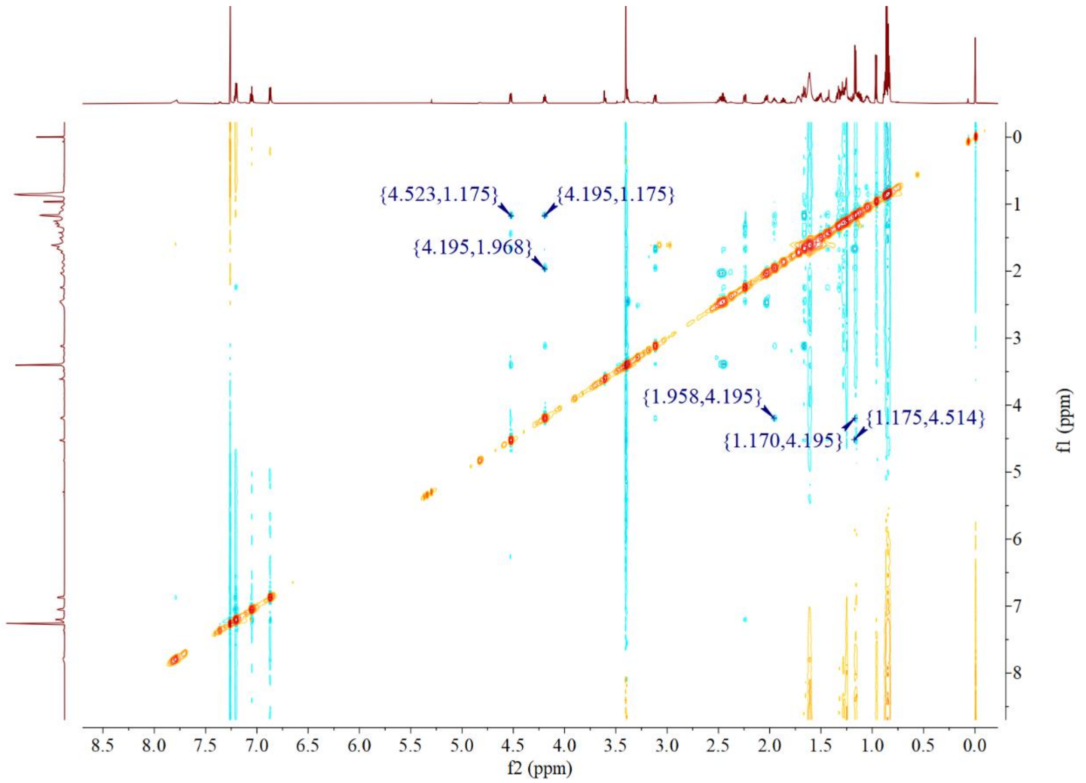

Figure S26. NOESY spectra ($CDCl_3$, 700 MHz) of compound **4**

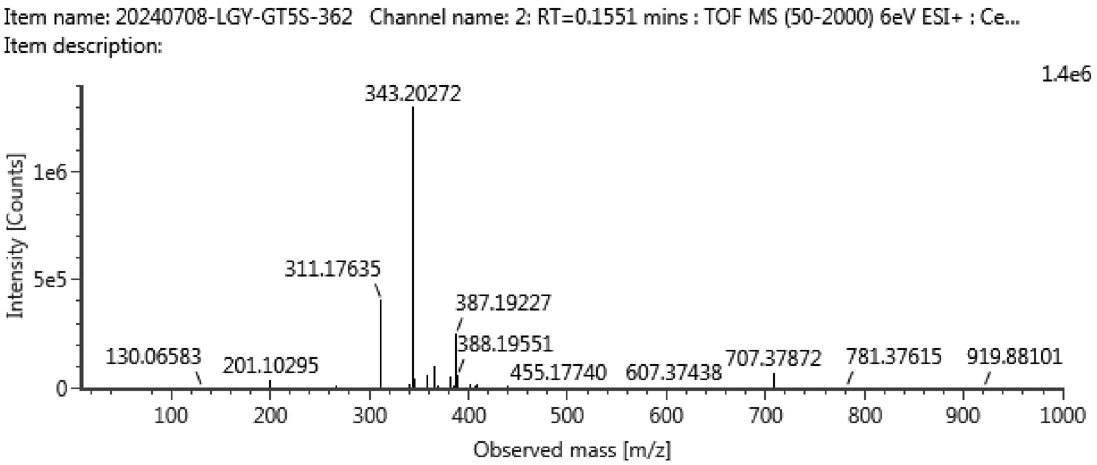

Figure S27. HRESIMS spectra of compound **4**

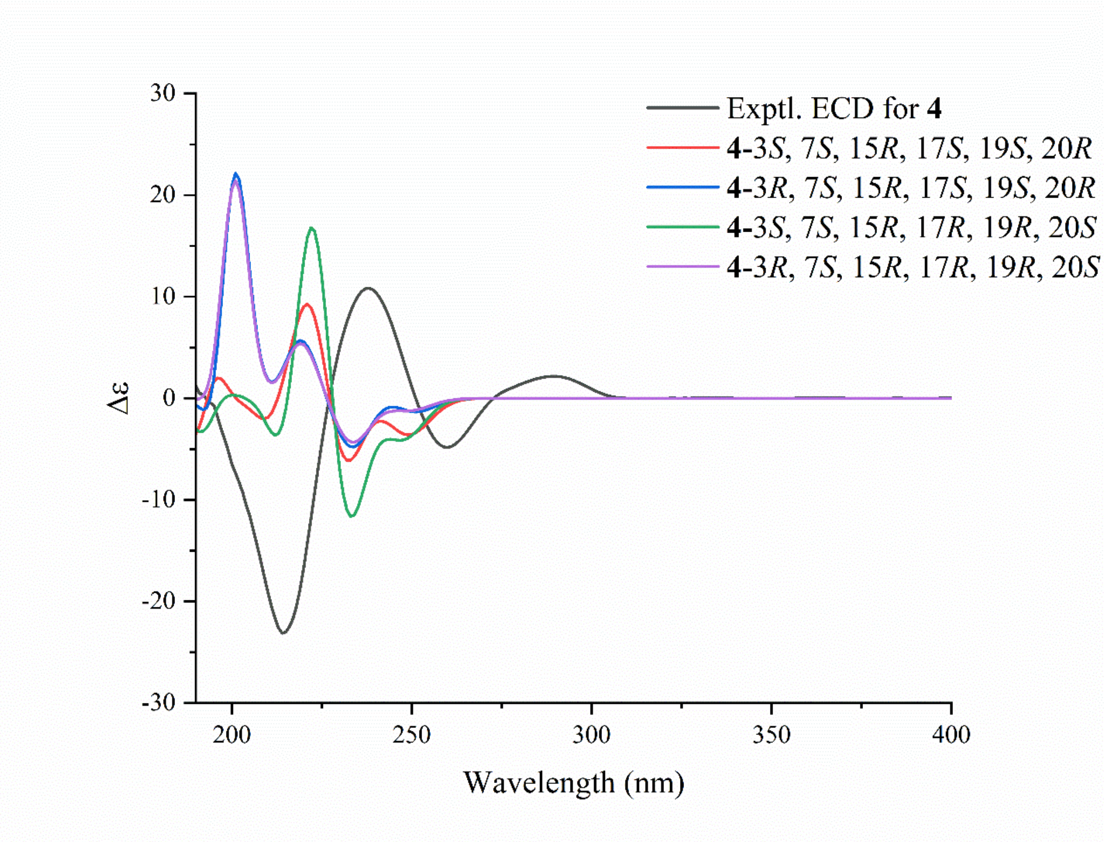

Figure S28. Experimental and calculated ECD spectra of compounds **4**

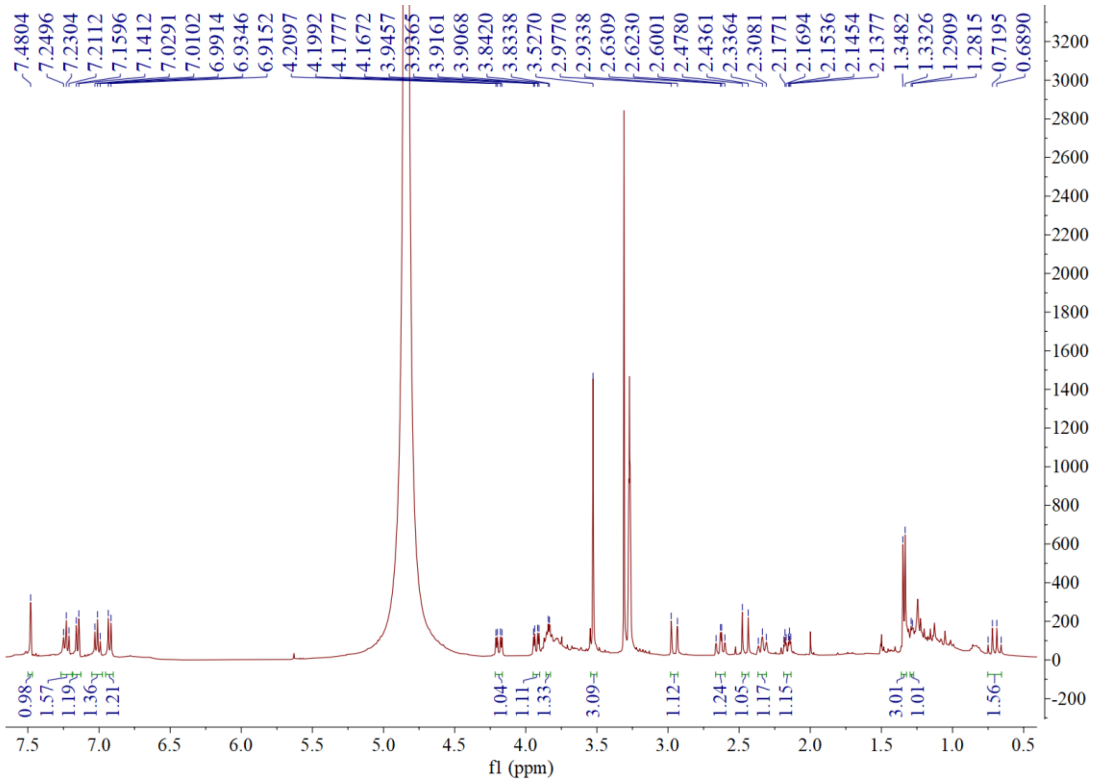

Figure S29. ^{1}H NMR spectra (CD_3OD, 400 MHz) of compound **5**

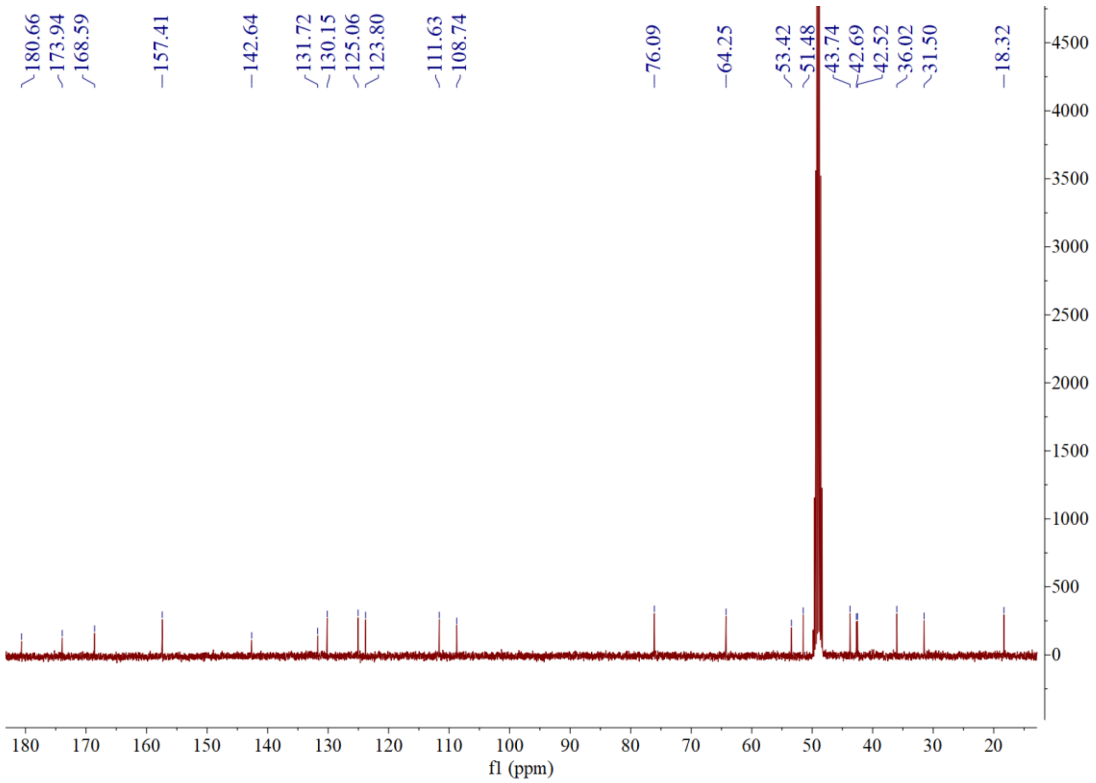

Figure S30. ^{13}C NMR spectra (CD_3OD, 100 MHz) of compound **5**

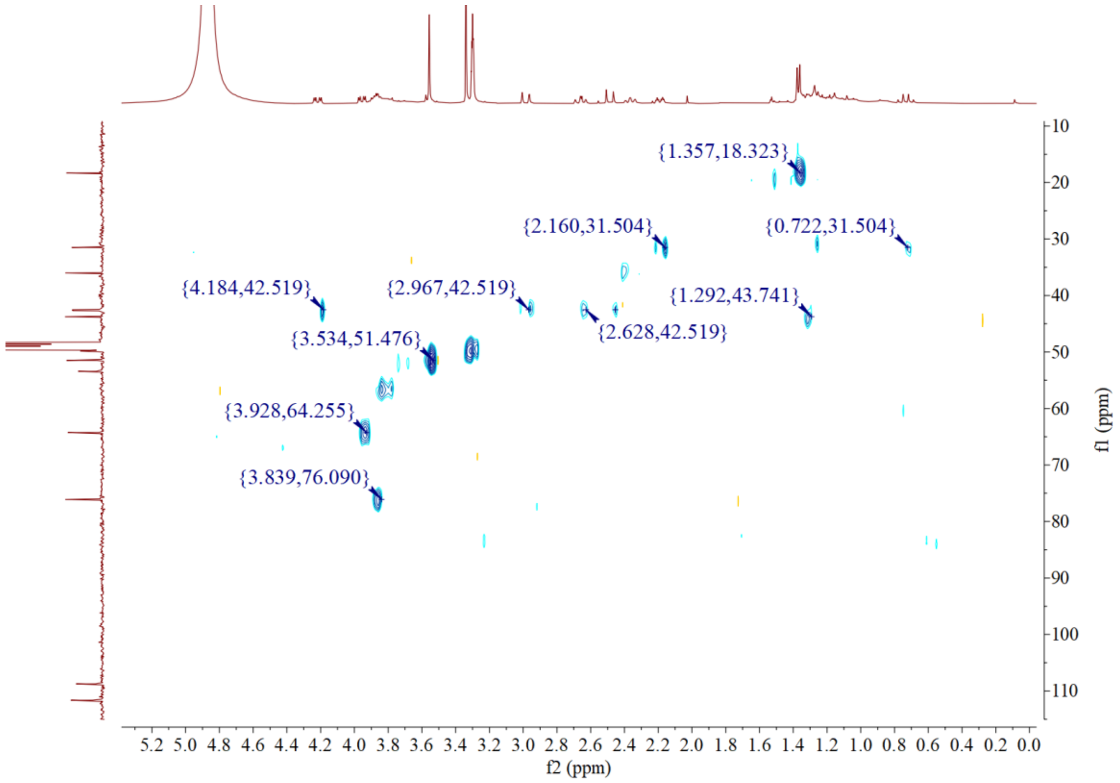

Figure S31. HSQC spectra (CD_3OD, 600 MHz) of compound **5**

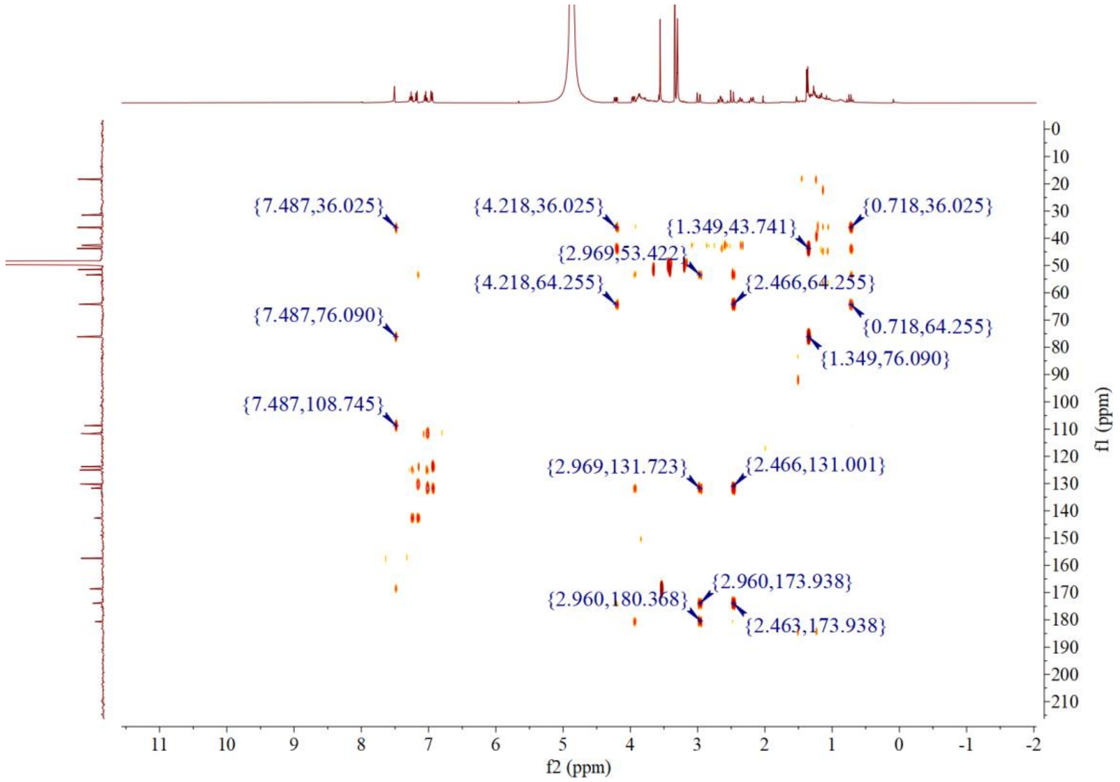

Figure S32. HMBC spectra (CD_3OD, 600 MHz) of compound **5**

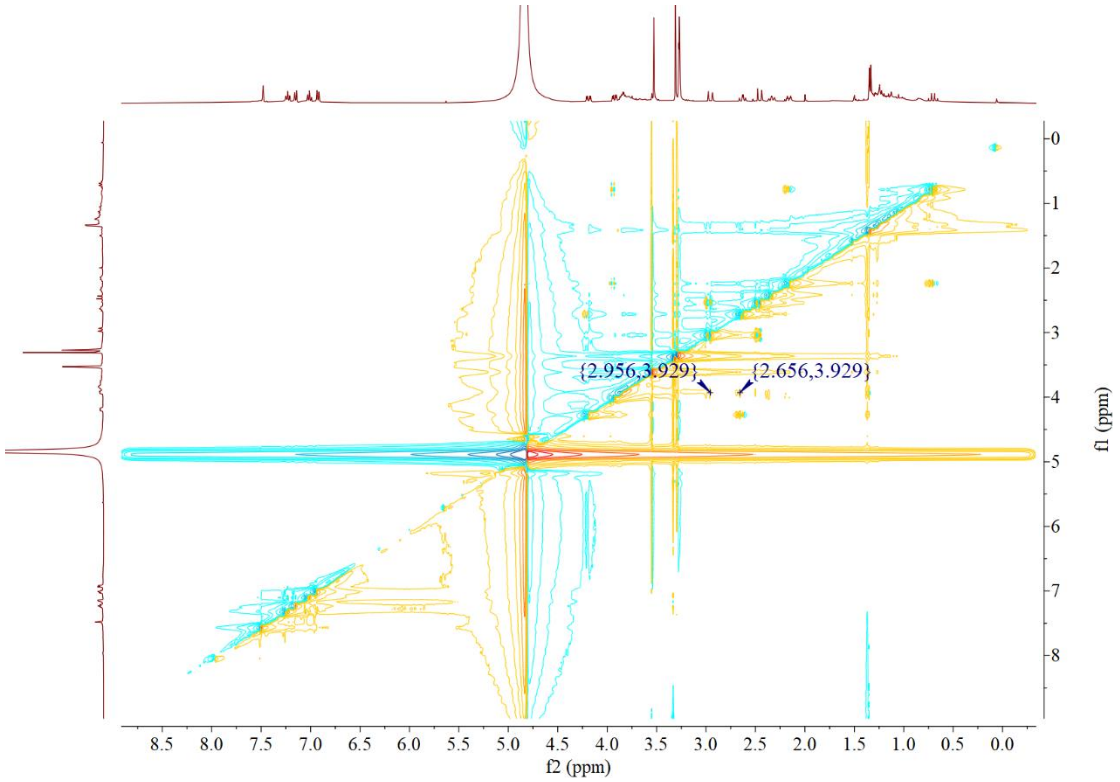

Figure S33. NOESY spectra (CD_3OD, 600 MHz) of compound **5**

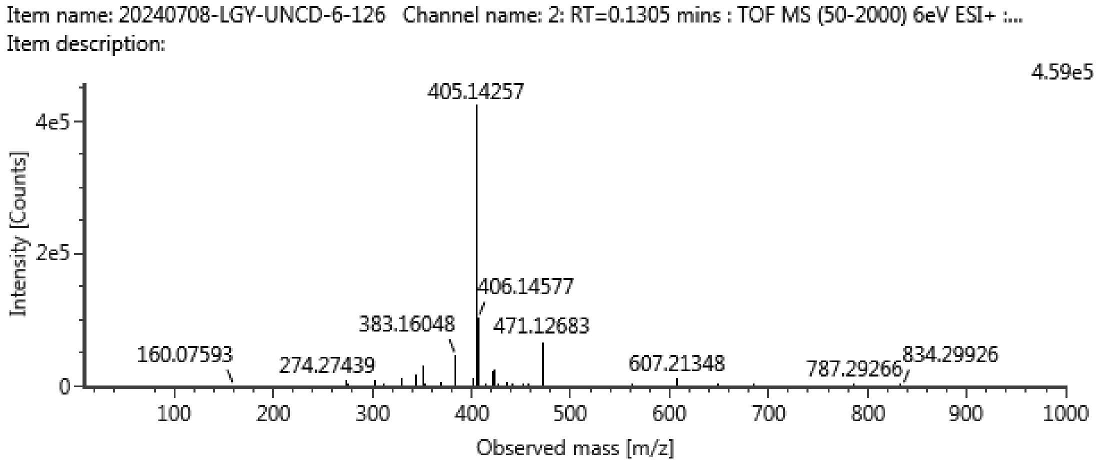

Figure S34. HRESIMS spectra of compound **5**

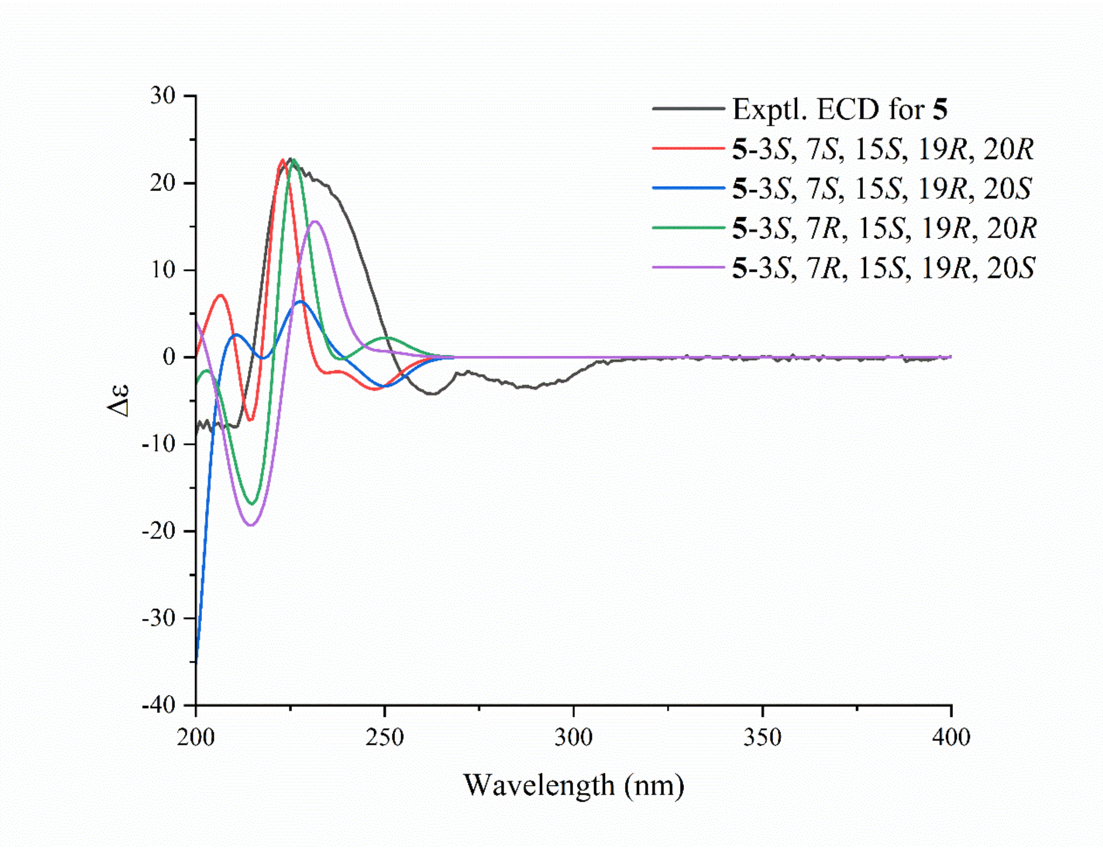

Figure S35. Experimental and calculated ECD spectra of compounds **5**

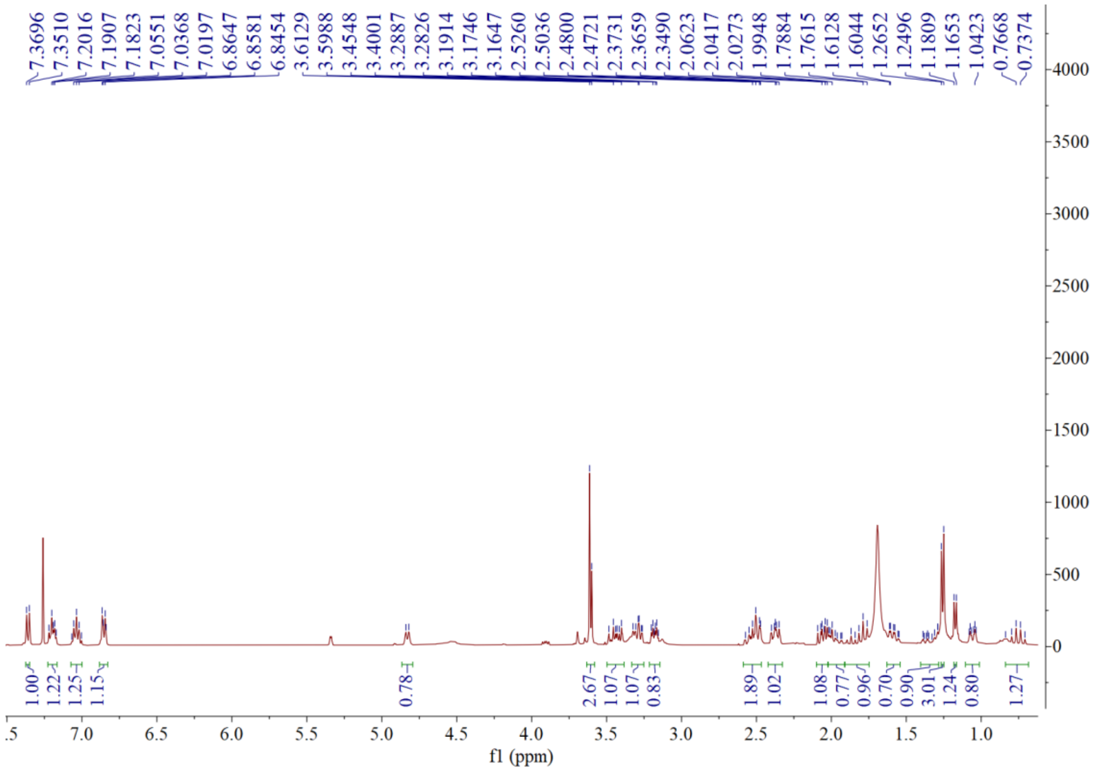

Figure S36. ^{1}H NMR spectra ($CDCl_3$, 400 MHz) of compound **6**

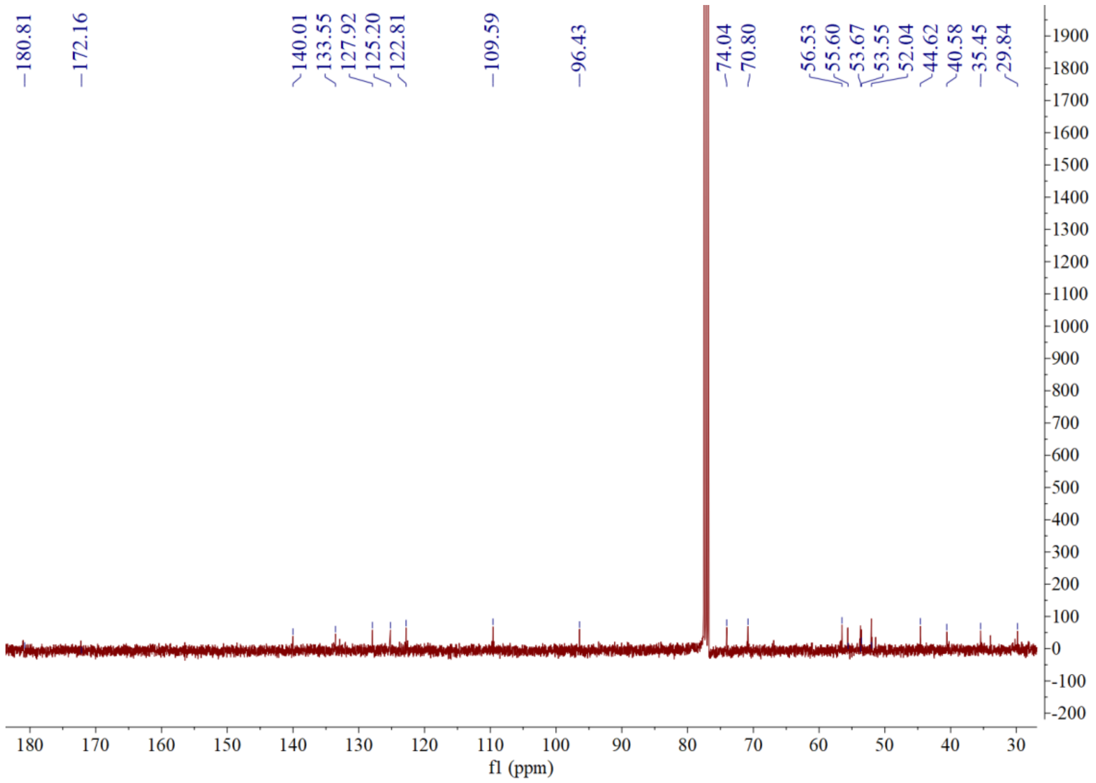

Figure S37. ^{13}C NMR spectra ($CDCl_3$, 100 MHz) of compound **6**

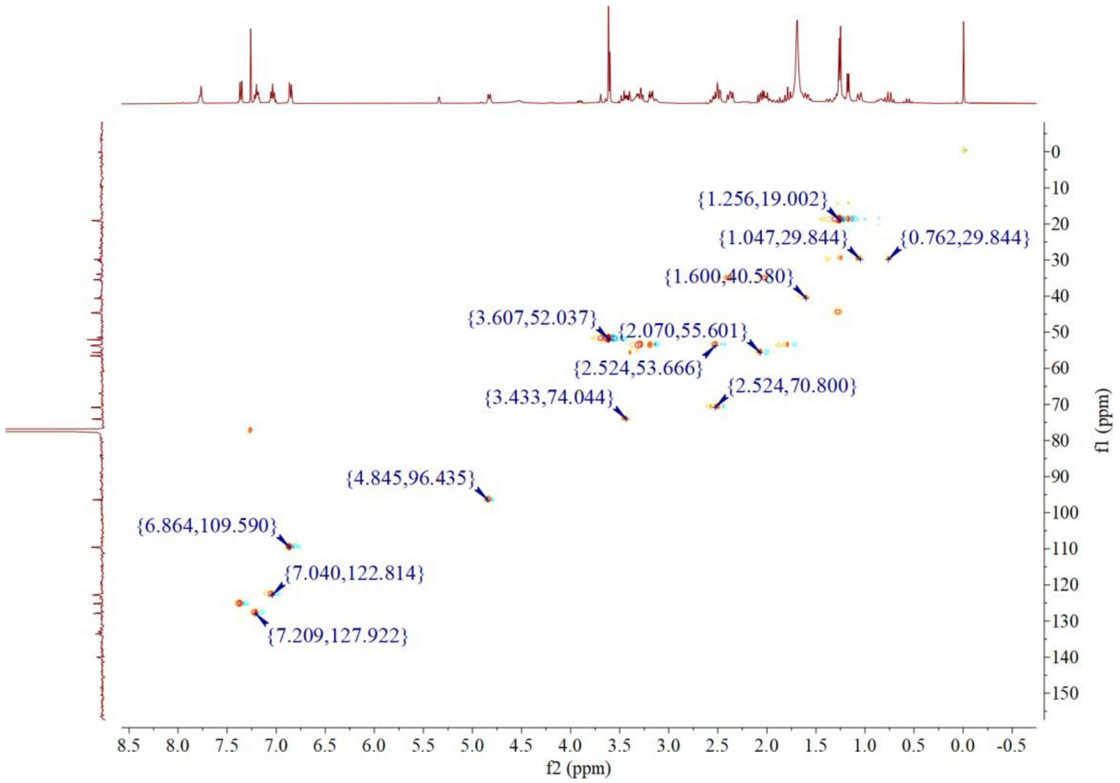

Figure S38. HSQC spectra ($CDCl_3$, 400 MHz) of compound **6**

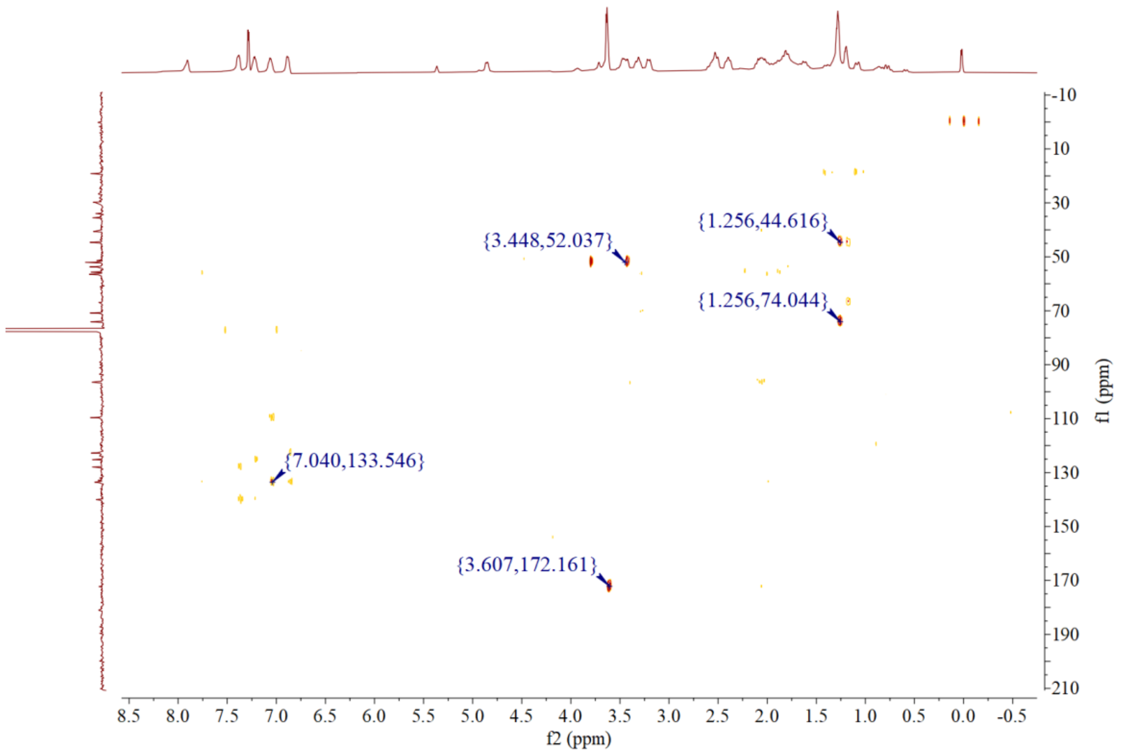

Figure S39. HMBC spectra ($CDCl_3$, 400 MHz) of compound **6**

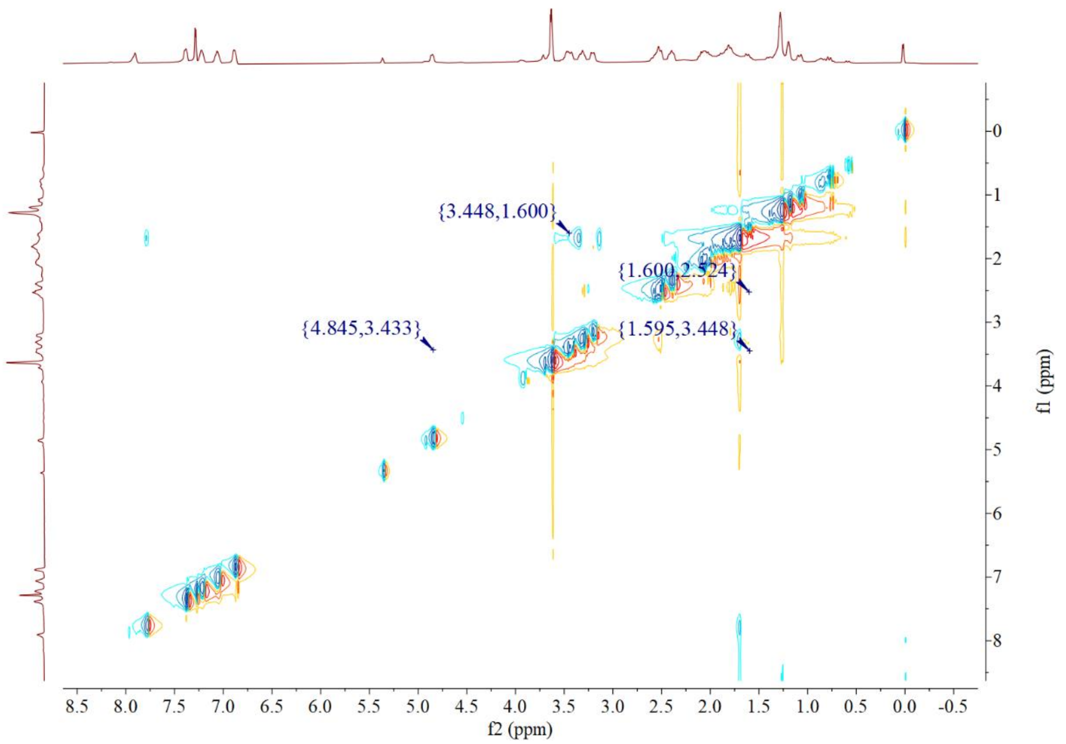

Figure S40. NOESY spectra (CDCl3, 400 MHz) of compound **6**

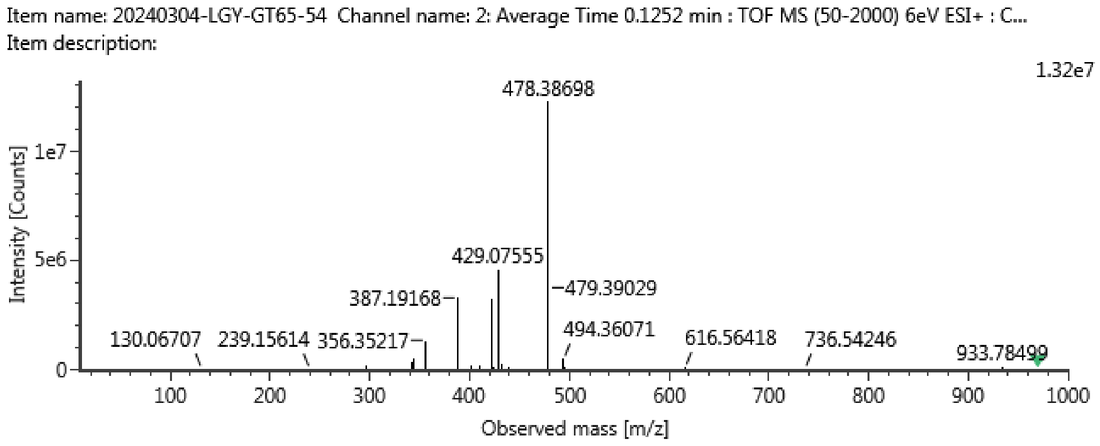

Figure S41. HRESIMS spectra of compound **6**

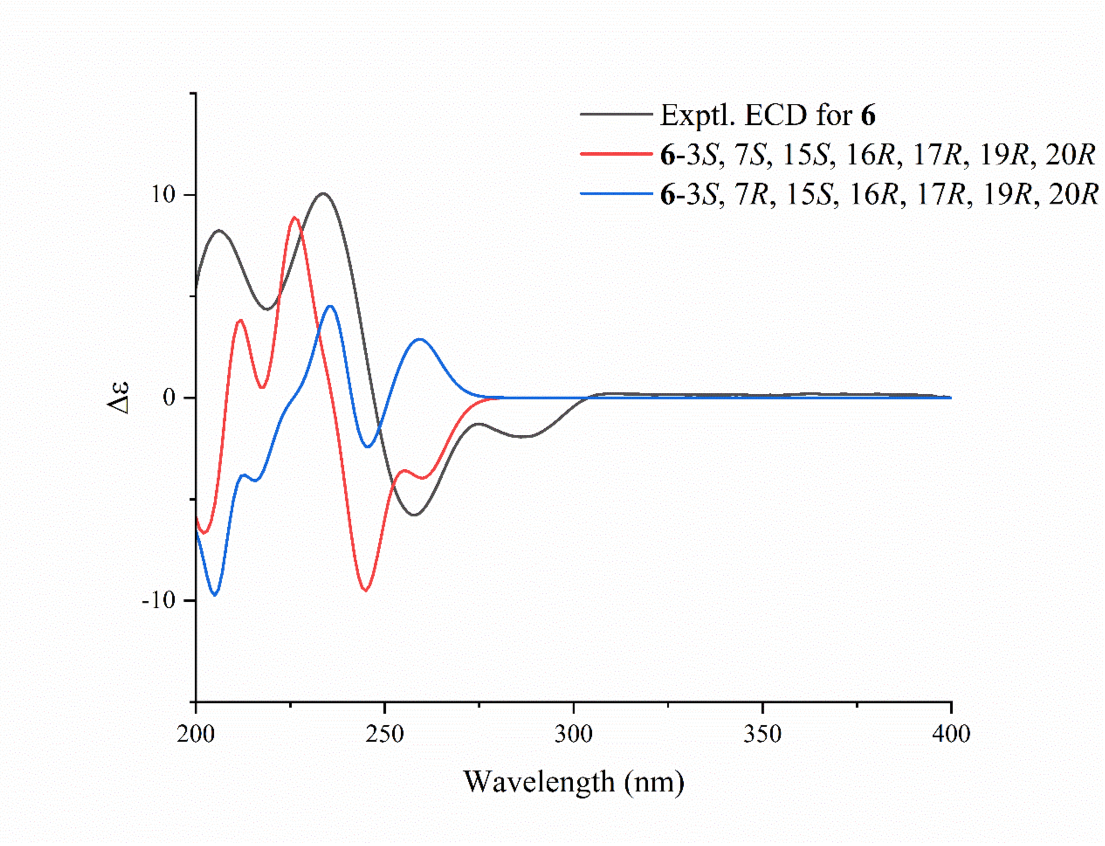

Figure S42. Experimental and calculated ECD spectra of compounds **6**

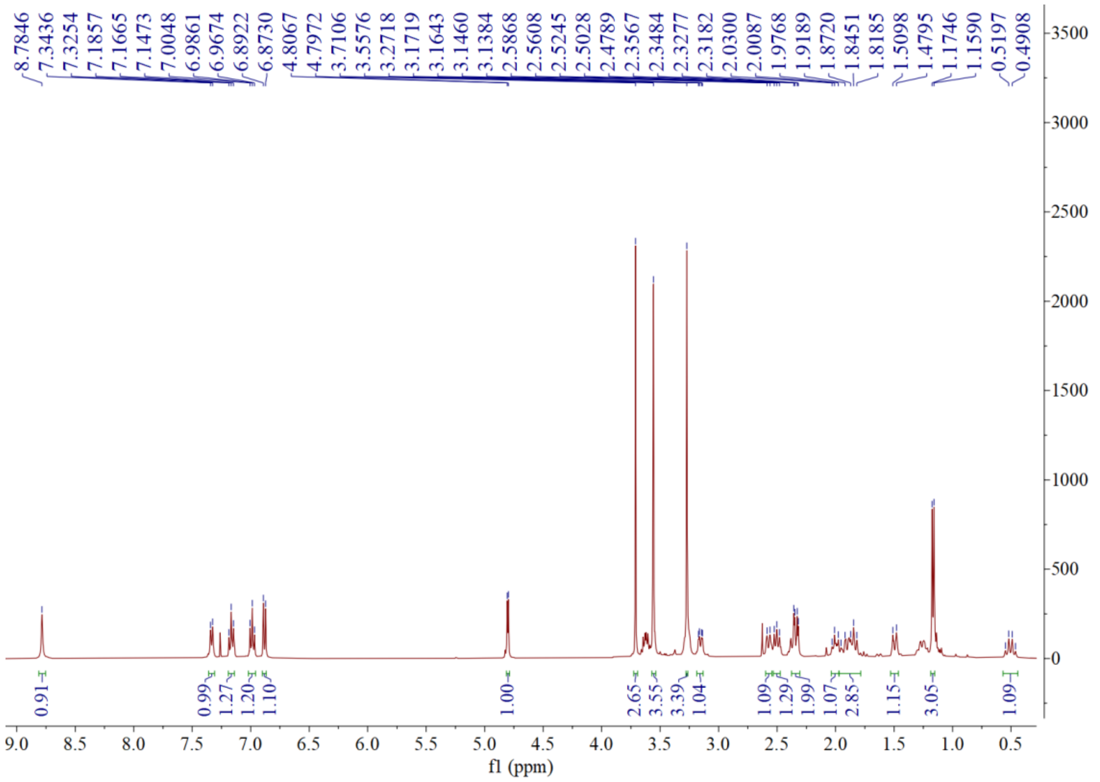

Figure S43. ^{1}H NMR spectra ($CDCl_3$, 400 MHz) of compound **7**

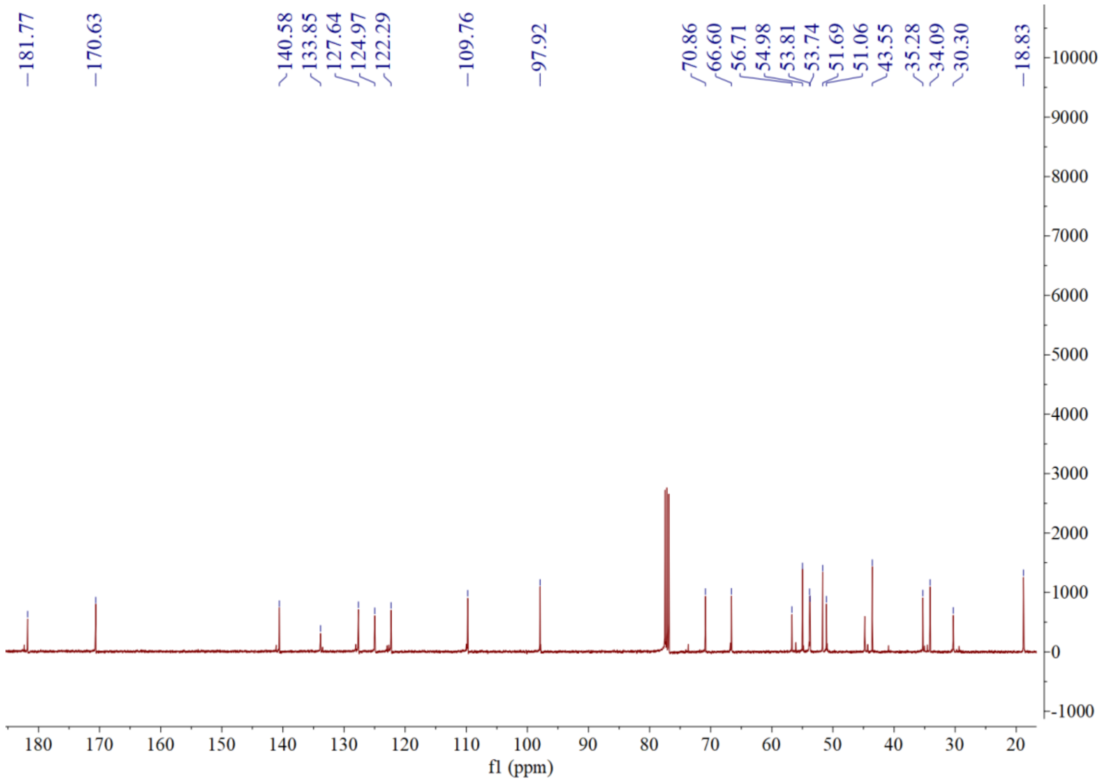

Figure S44. ^{13}C NMR spectra ($CDCl_3$, 100 MHz) of compound **7**

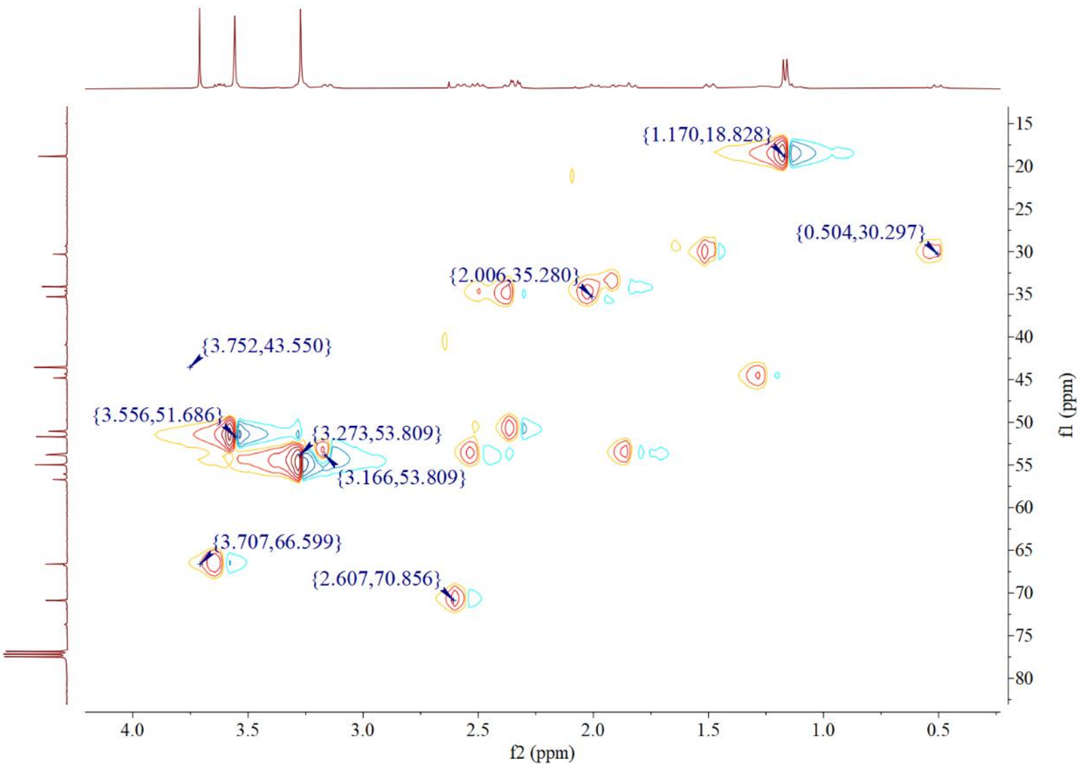

Figure S45. HSQC spectra ($CDCl_3$, 400 MHz) of compound **7**

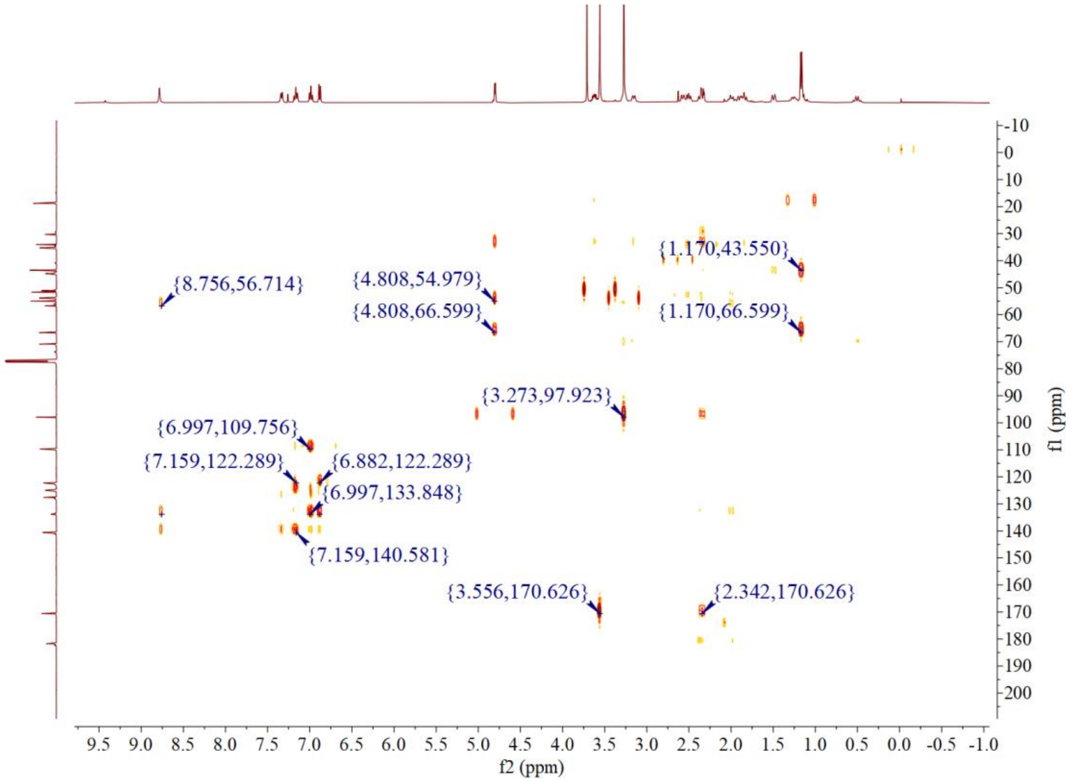

Figure S46. HMBC spectra ($CDCl_3$, 400 MHz) of compound **7**

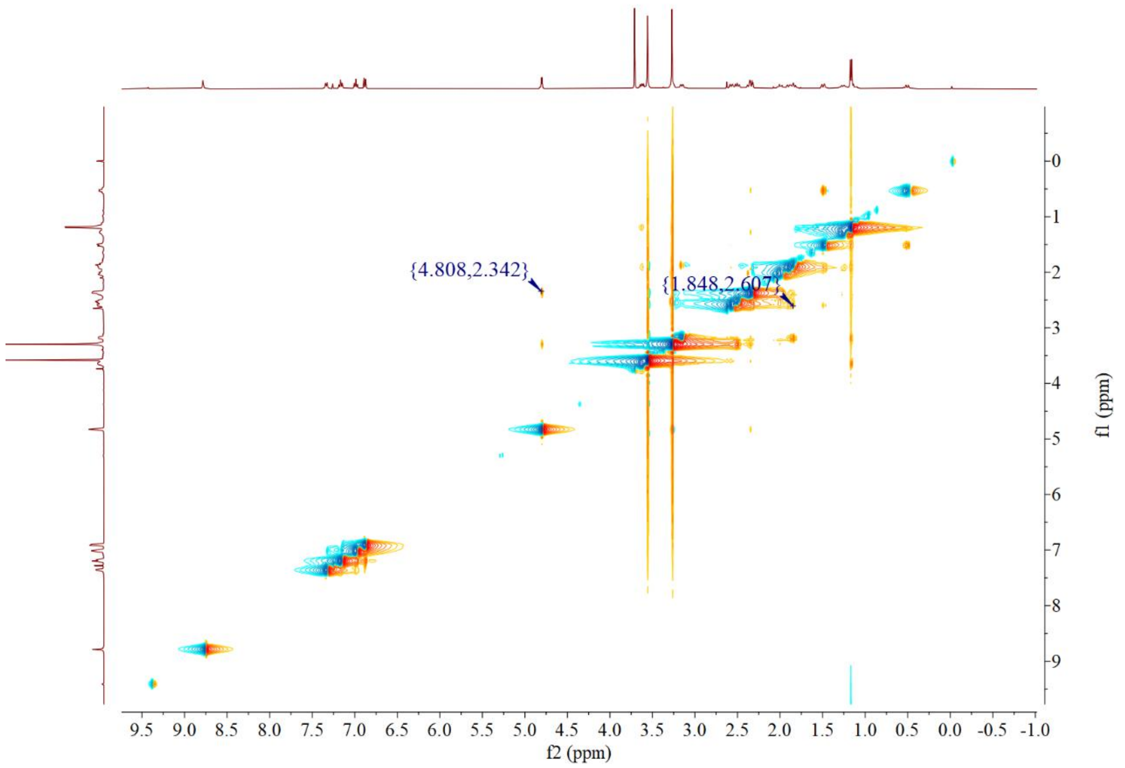

Figure S47. NOESY spectra ($CDCl_3$, 400 MHz) of compound **7**

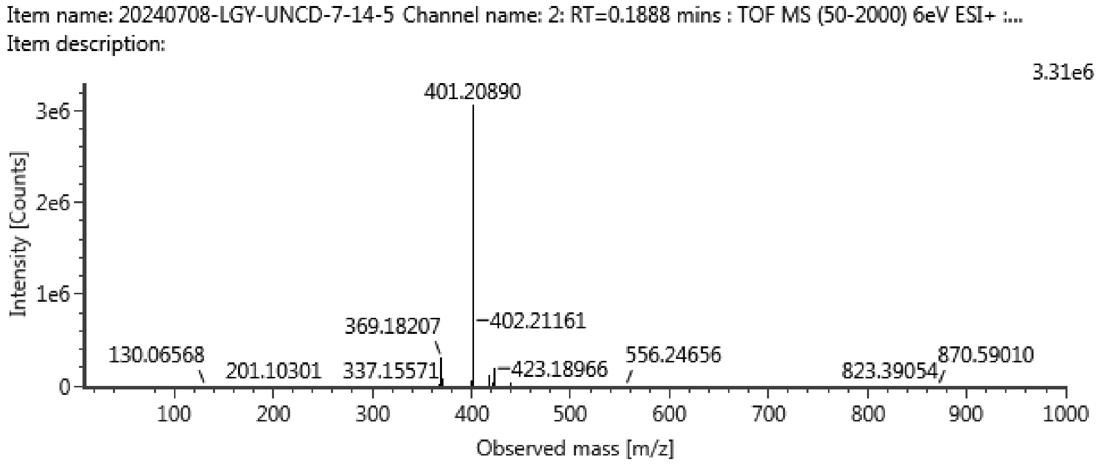

Figure S48. HRESIMS spectra of compound **7**

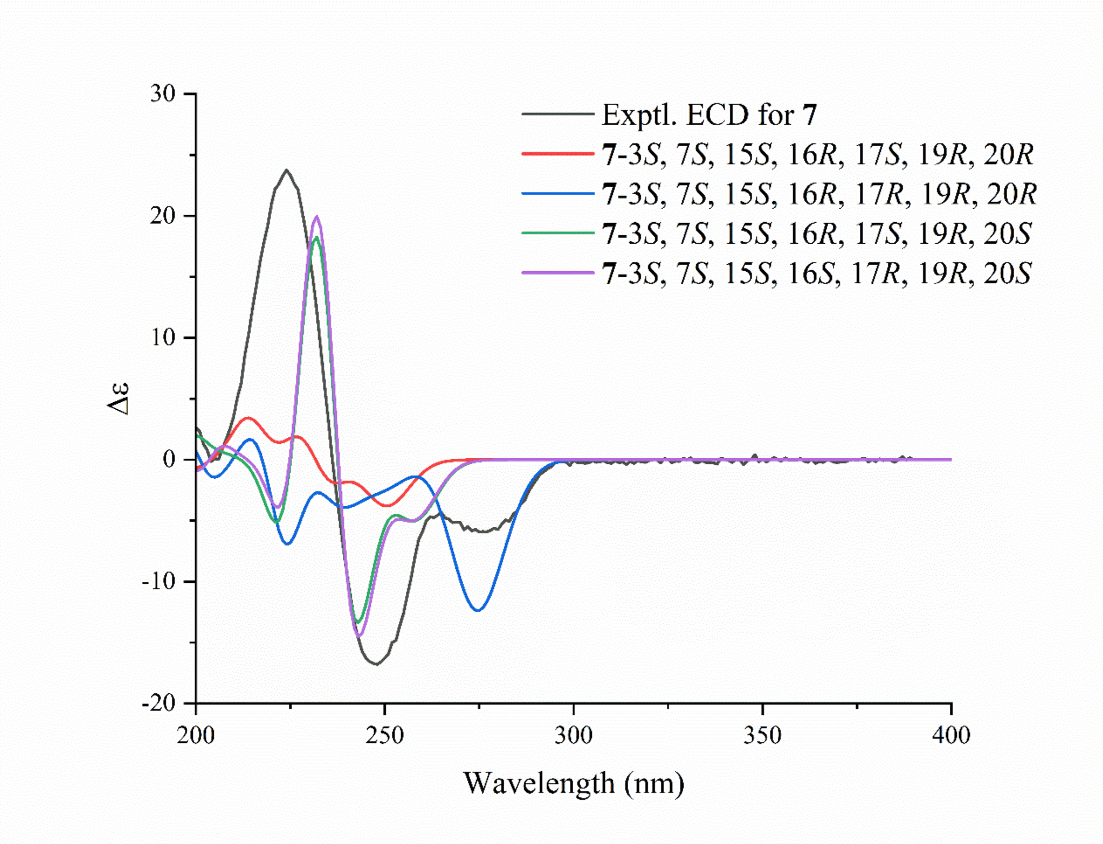

Figure S49. Experimental and calculated ECD spectra of compounds **7**

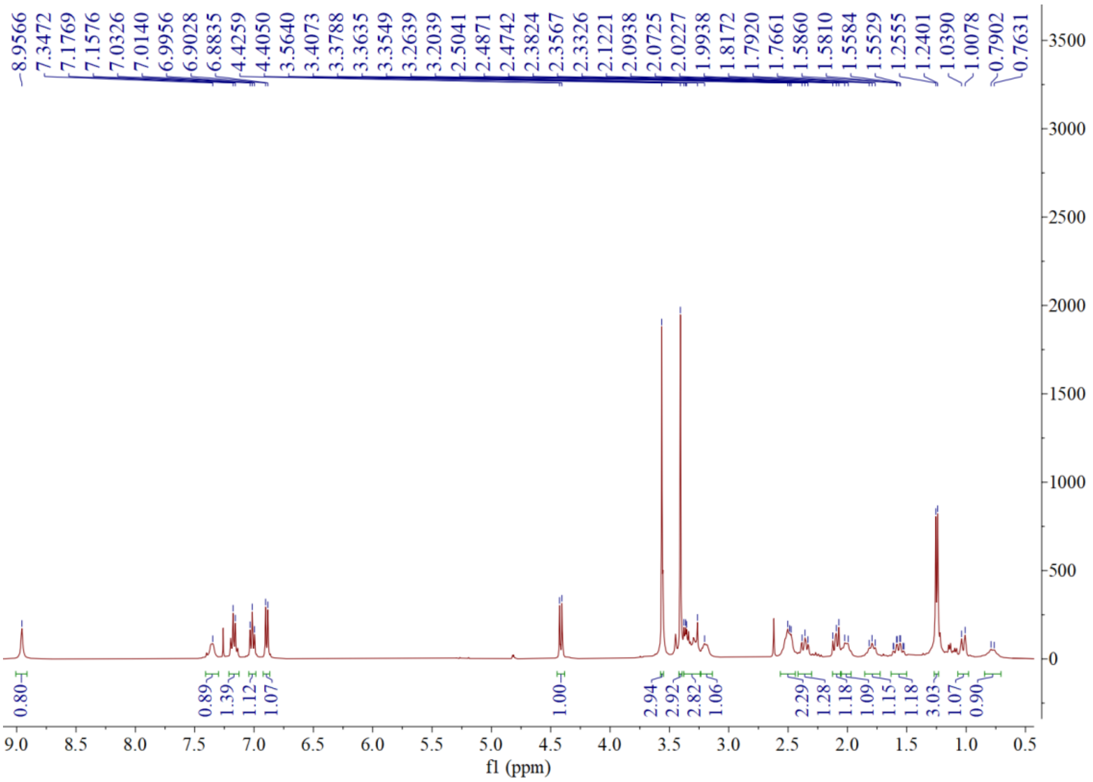

Figure S50. ^{1}H NMR spectra ($CDCl_3$, 400 MHz) of compound **8**

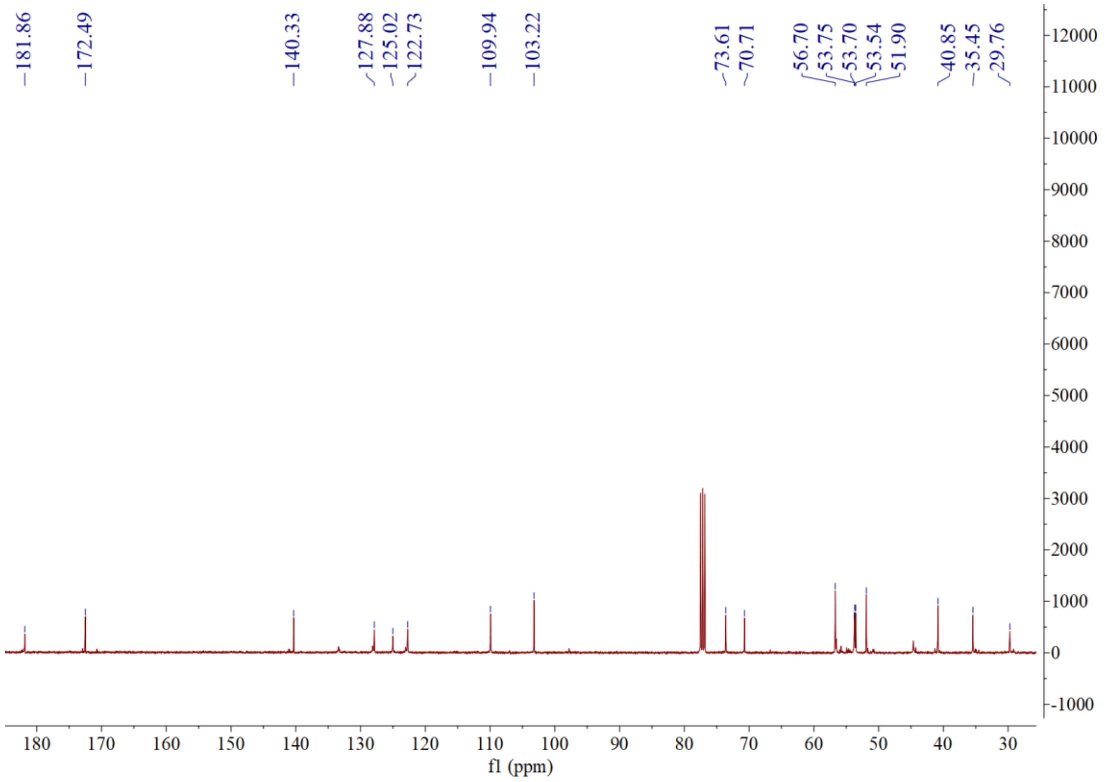

Figure S51. ^{13}C NMR spectra ($CDCl_3$, 100 MHz) of compound **8**

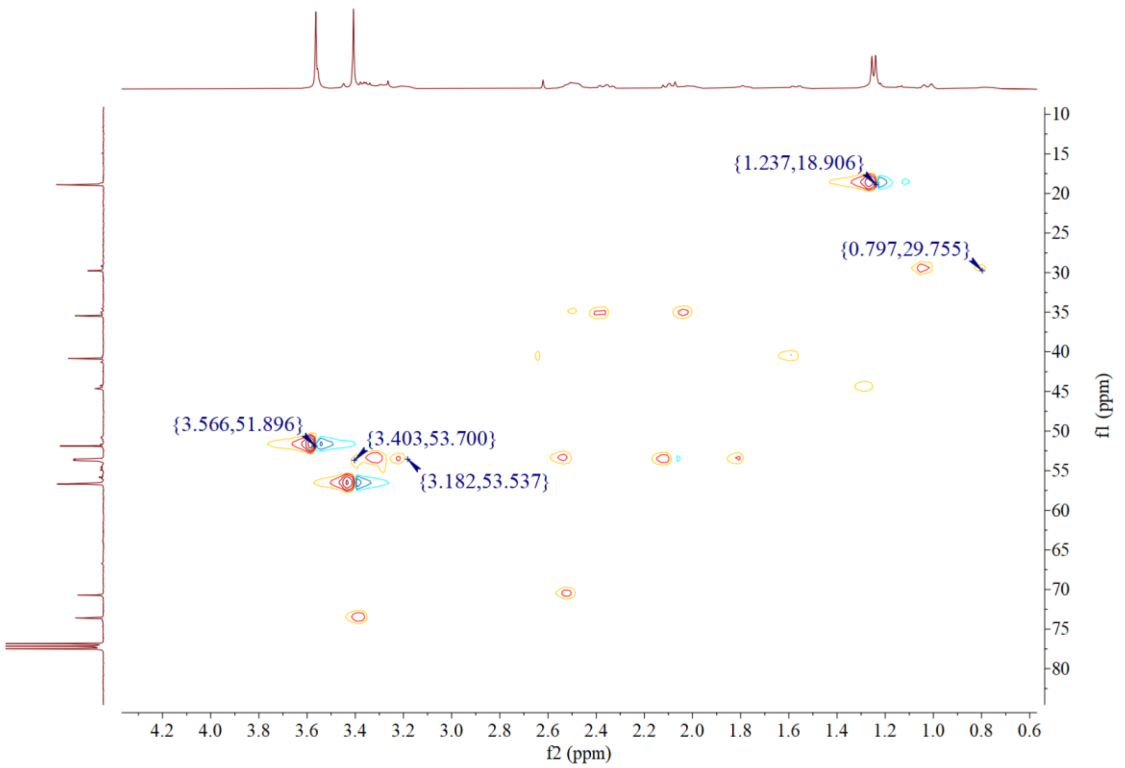

Figure S52. HSQC spectra ($CDCl_3$, 400 MHz) of compound **8**

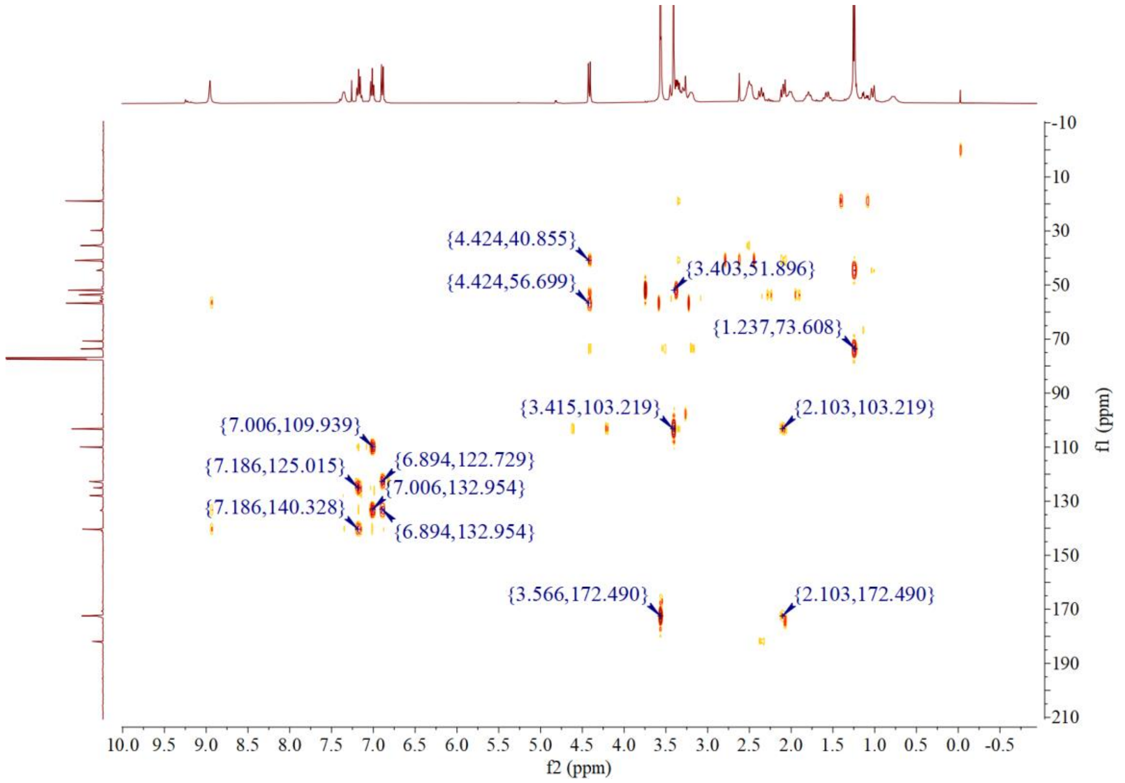

Figure S53. HMBC spectra ($CDCl_3$, 400 MHz) of compound **8**

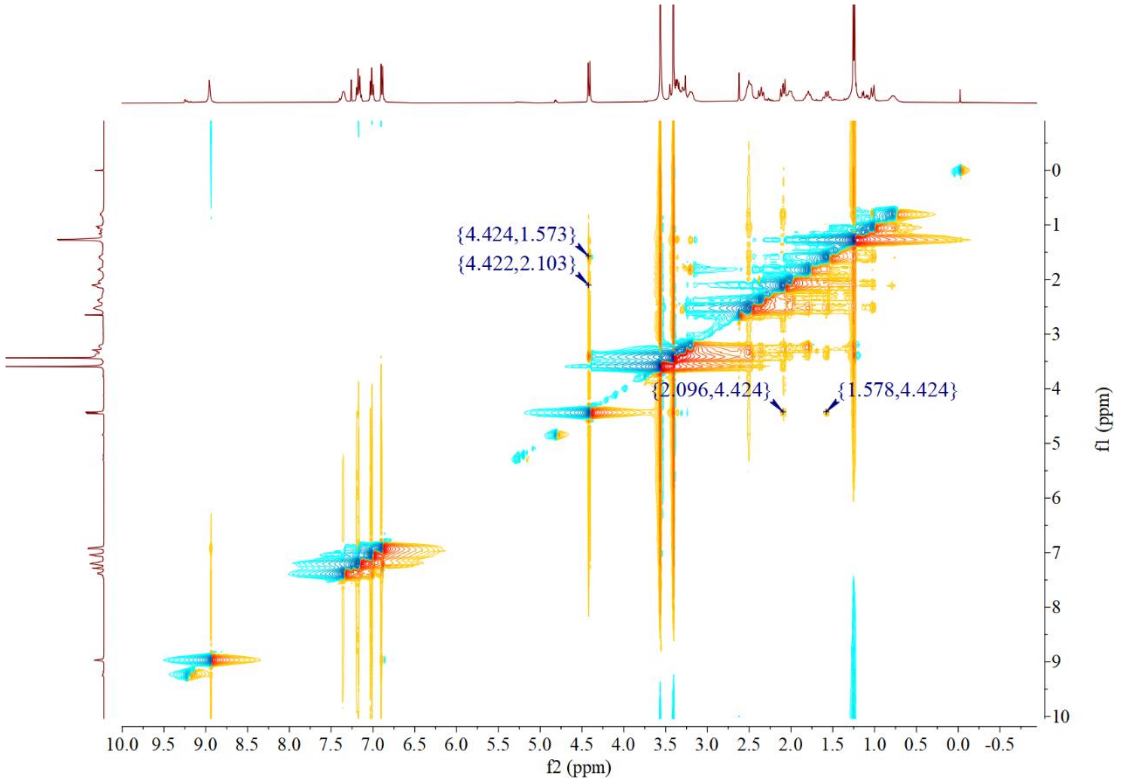

Figure S54. NOESY spectra ($CDCl_3$, 400 MHz) of compound **8**

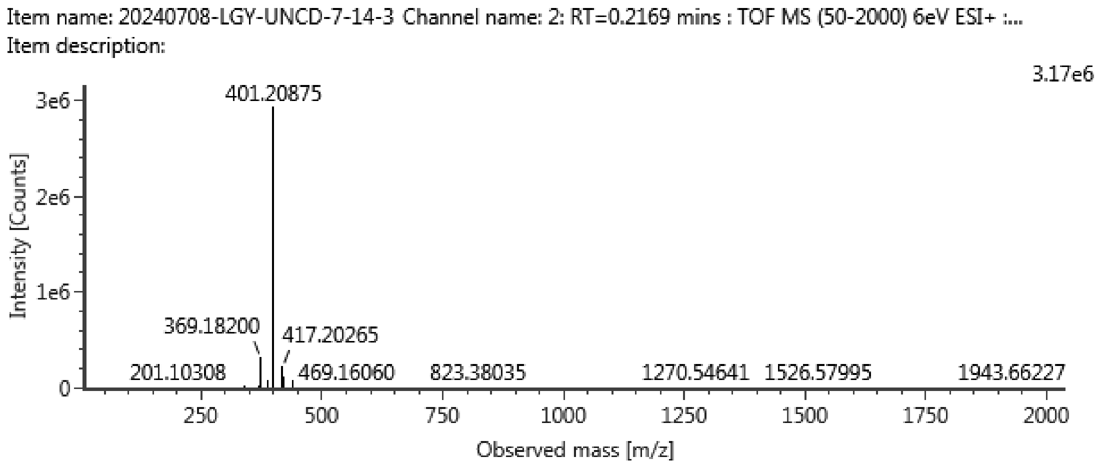

Figure S55. HRESIMS spectra of compound **8**

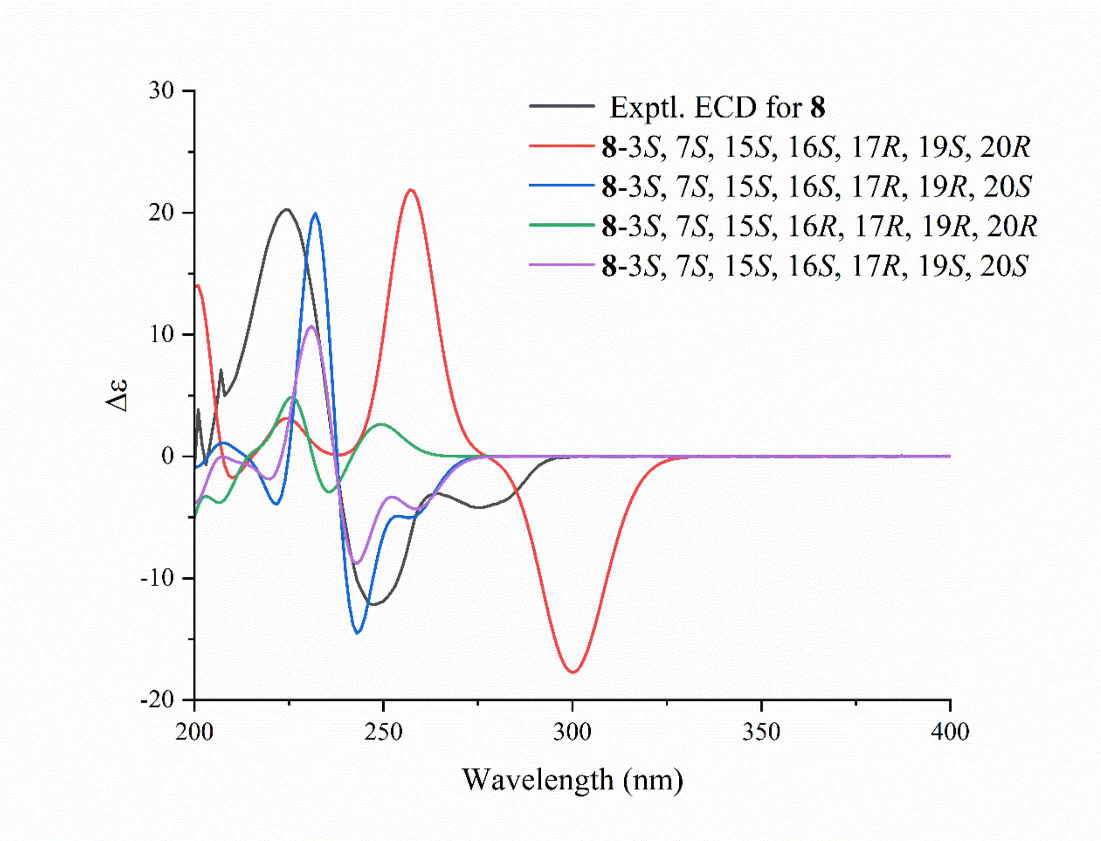

Figure S56. Experimental and calculated ECD spectra of compounds **8**

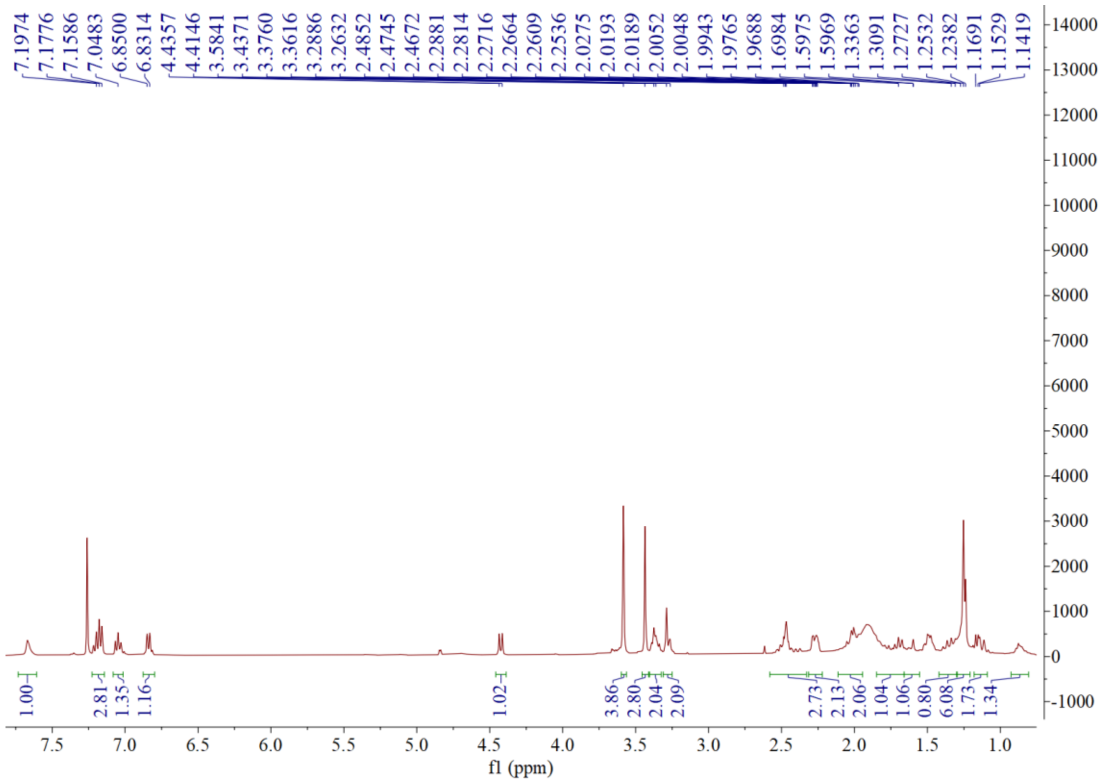

Figure S57. ^{1}H NMR spectra ($CDCl_3$, 400 MHz) of compound **9**

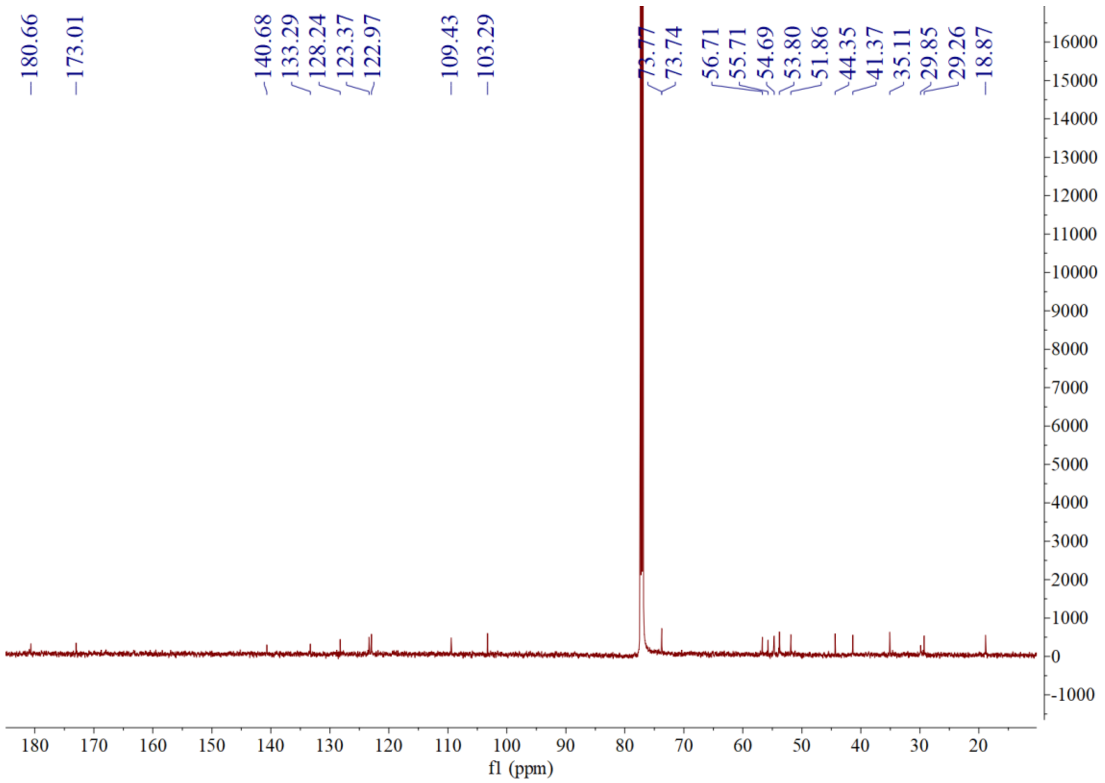

Figure S58. ^{13}C NMR spectra ($CDCl_3$, 150 MHz) of compound **9**

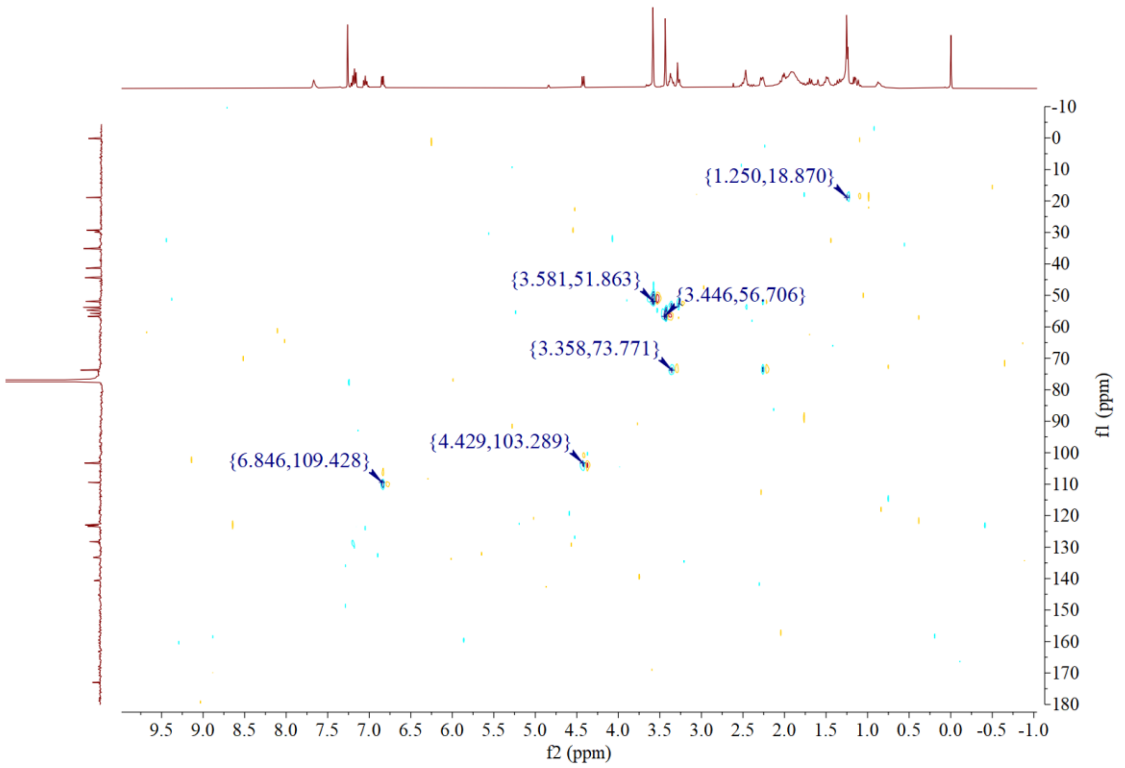

Figure S59. HSQC spectra ($CDCl_3$, 600 MHz) of compound **9**

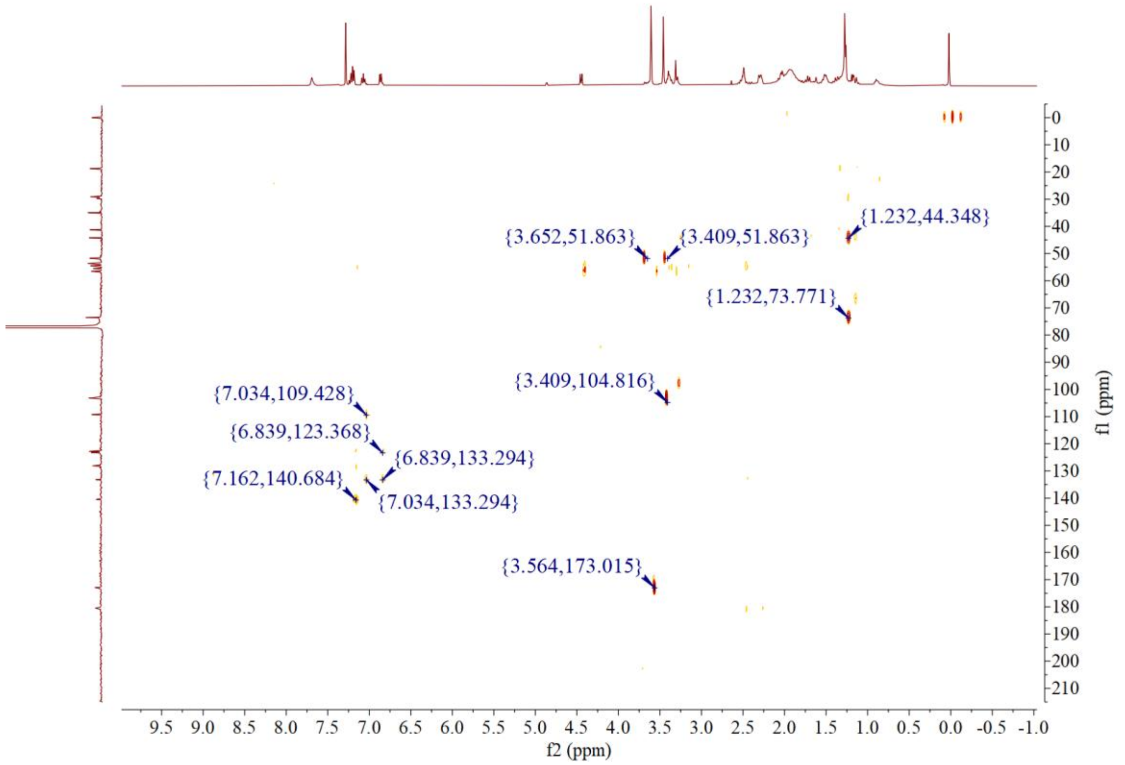

Figure S60. HMBC spectra ($CDCl_3$, 600 MHz) of compound **9**

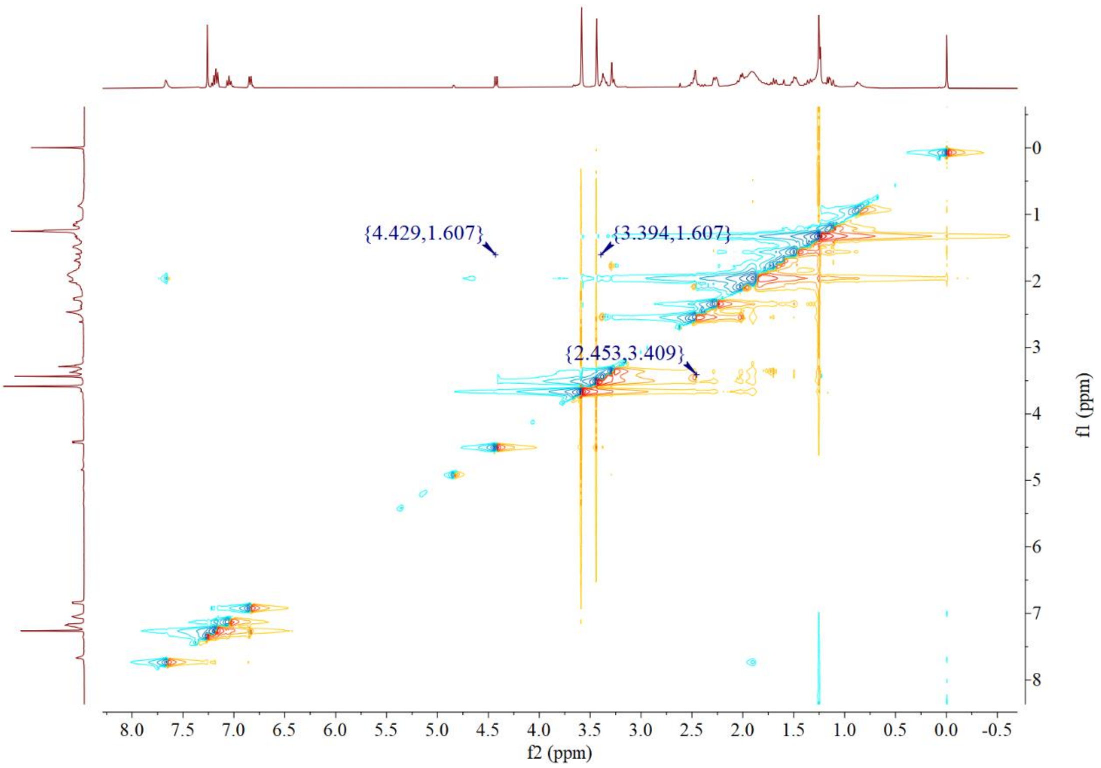

Figure S61. NOESY spectra ($CDCl_3$, 600 MHz) of compound **9**

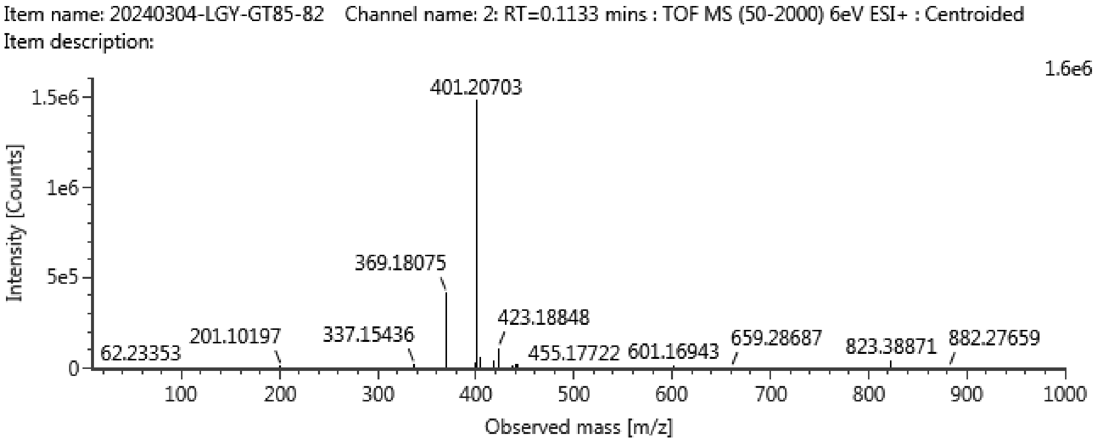

Figure S62. HRESIMS spectra of compound **9**

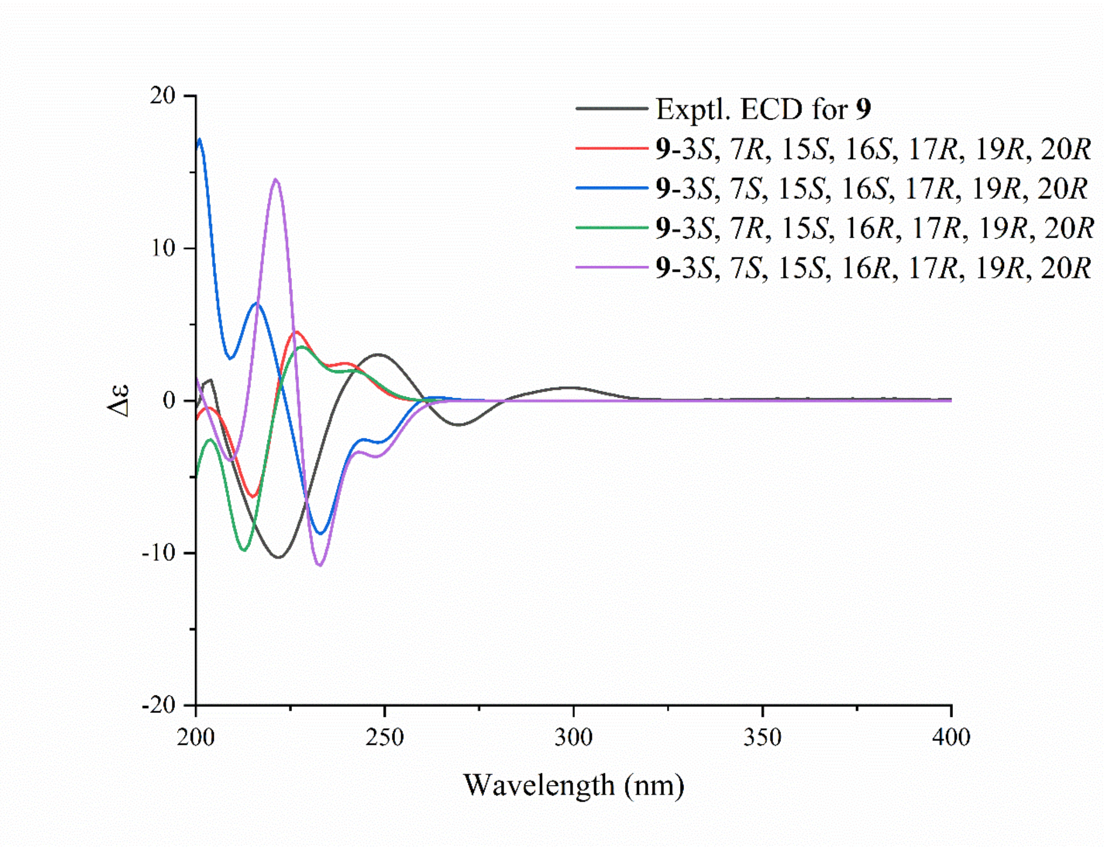

Figure S63. Experimental and calculated ECD spectra of compounds **9**

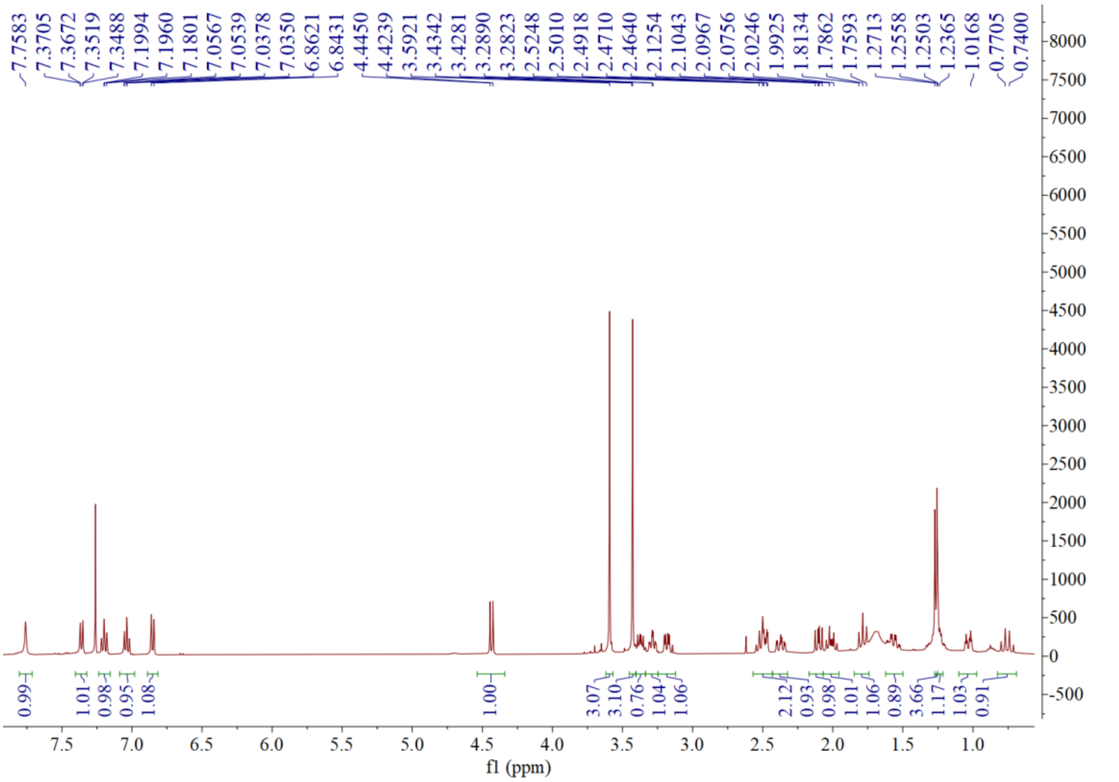

Figure S64. ^{1}H NMR spectra ($CDCl_3$, 400 MHz) of compound **10**

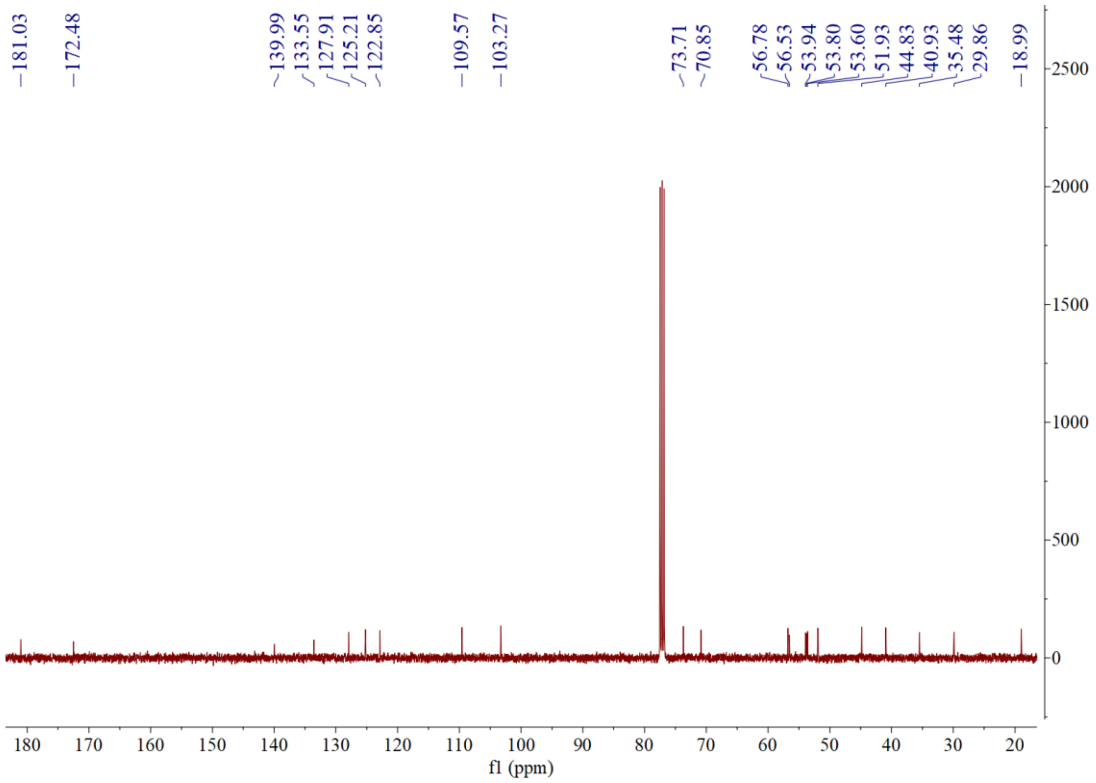

Figure S65. ^{13}C NMR spectra ($CDCl_3$, 100 MHz) of compound **10**

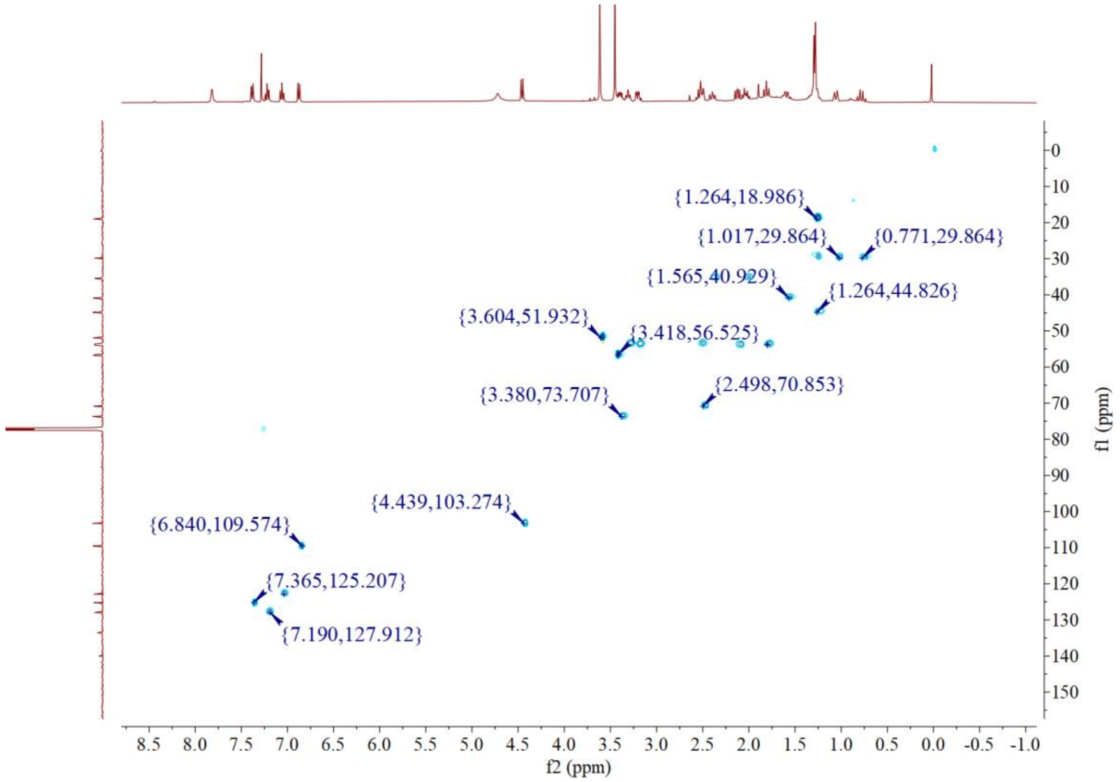

Figure S66. HSQC spectra ($CDCl_3$, 400 MHz) of compound **10**

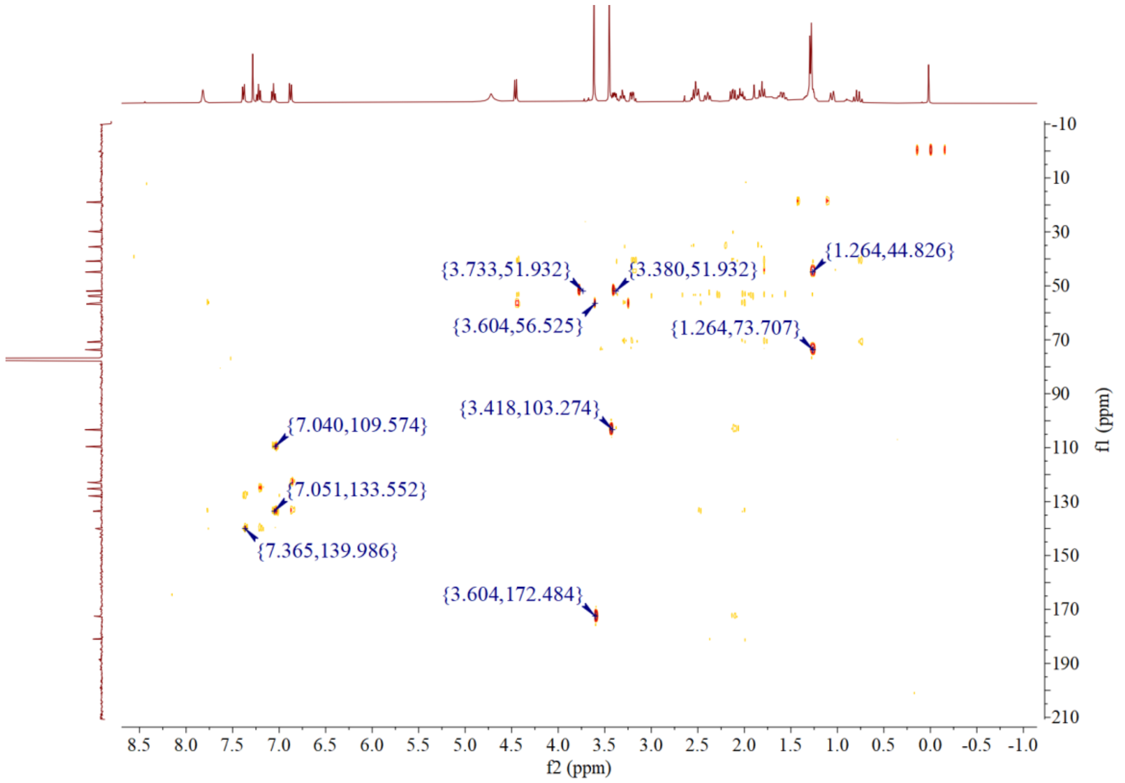

Figure S67. HMBC spectra ($CDCl_3$, 400 MHz) of compound **10**

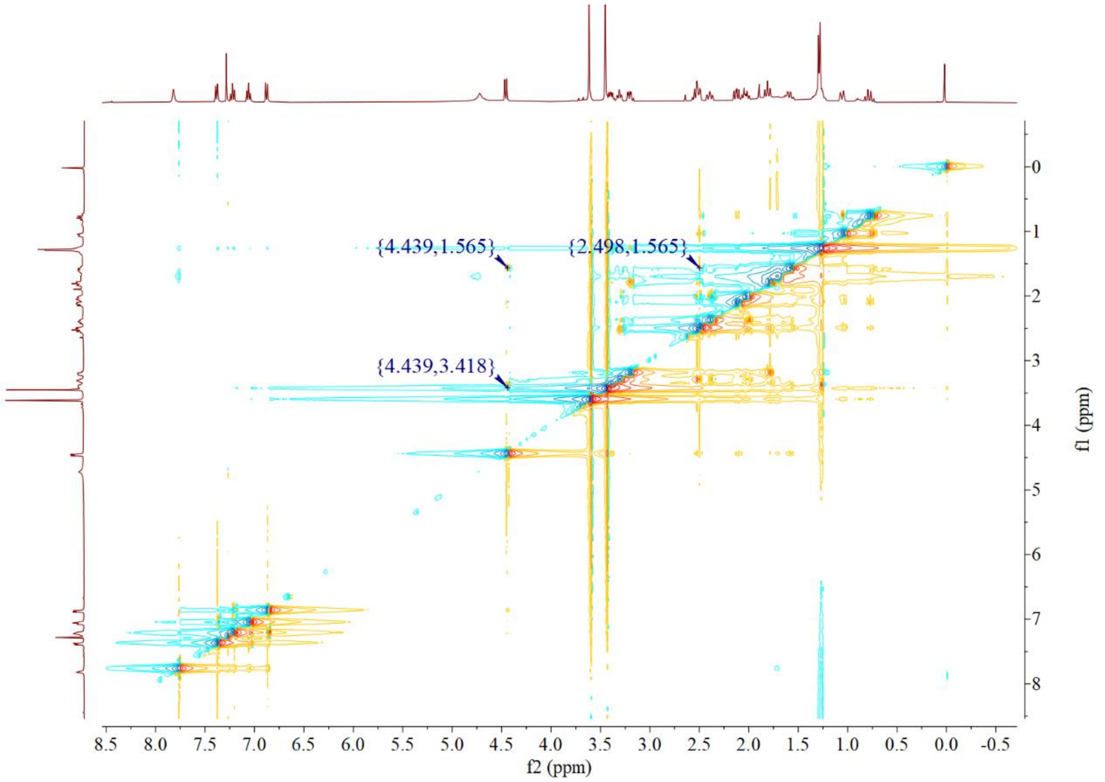

Figure S68. NOESY spectra ($CDCl_3$, 400 MHz) of compound **10**

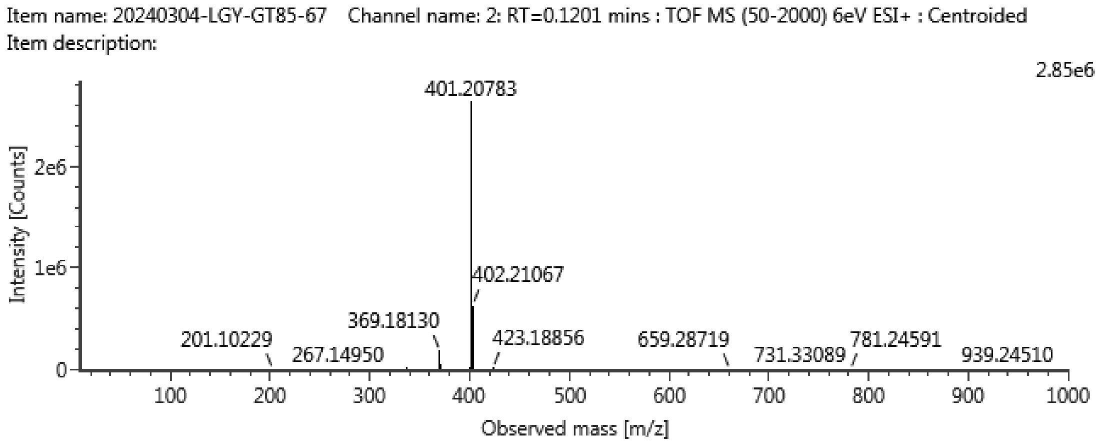

Figure S69. HRESIMS spectra of compound **10**

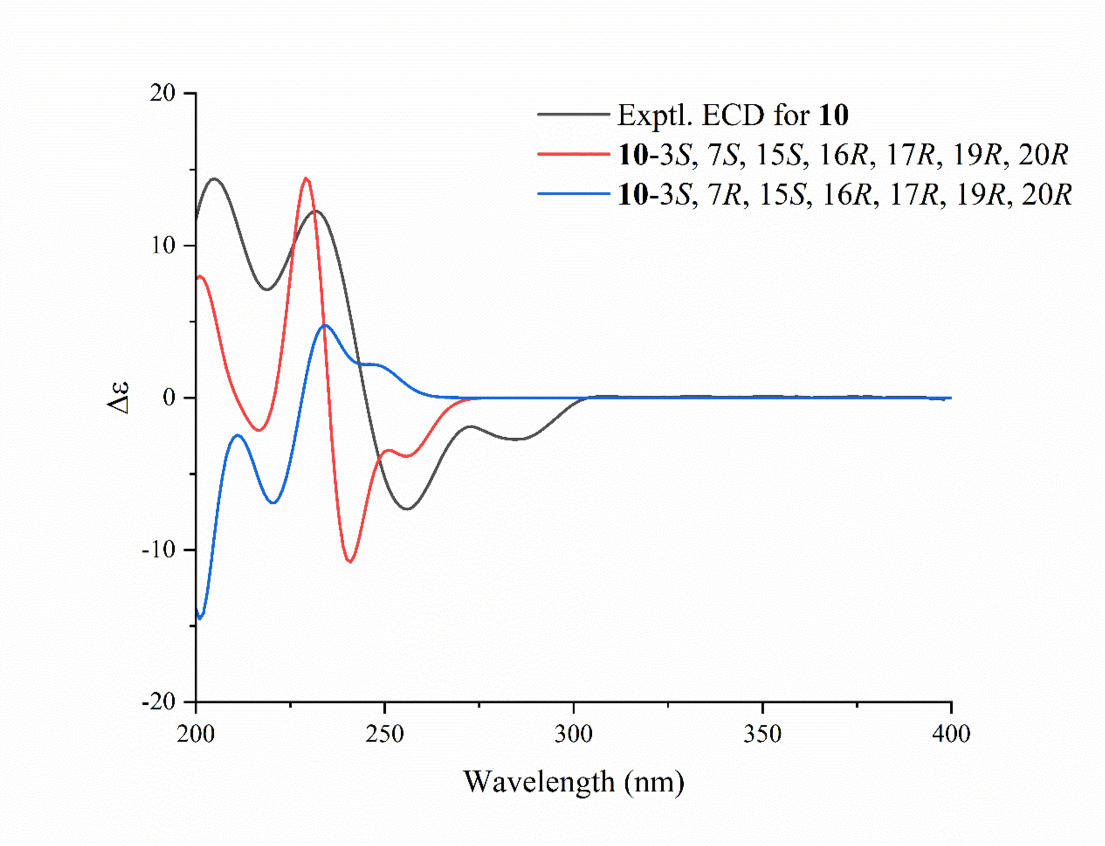

Figure S70. Experimental and calculated ECD spectra of compounds **10**

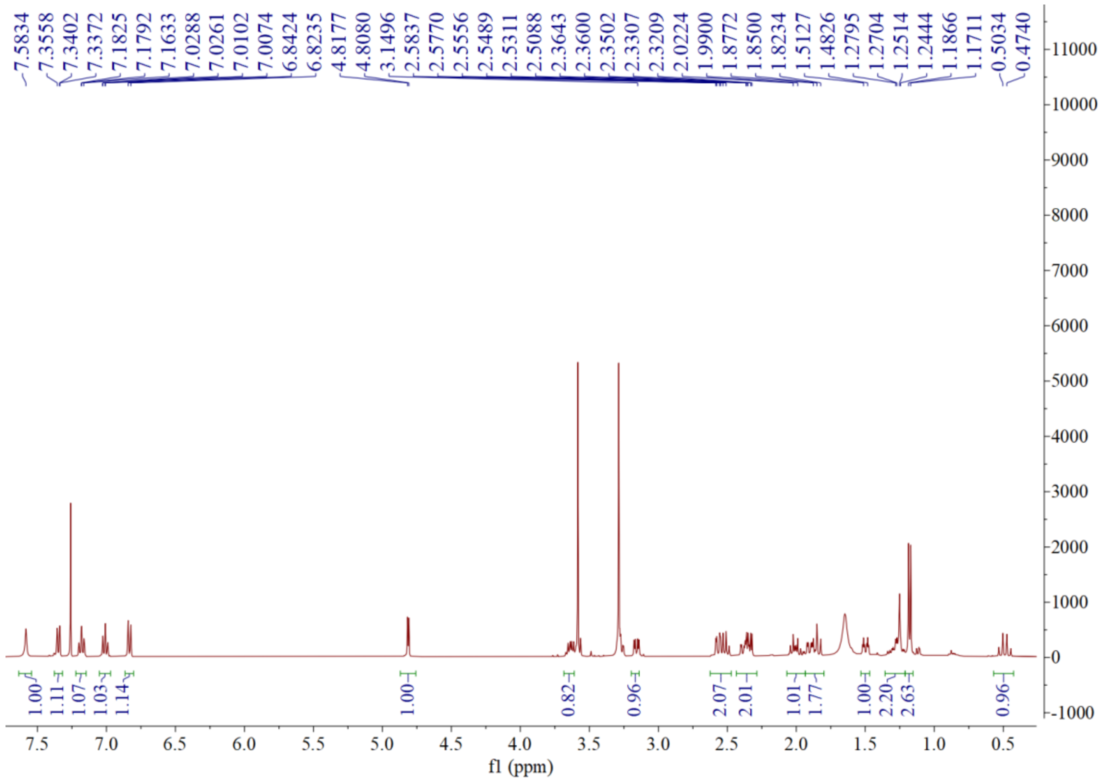

Figure S71. ^{1}H NMR spectra ($CDCl_3$, 400 MHz) of compound **11**

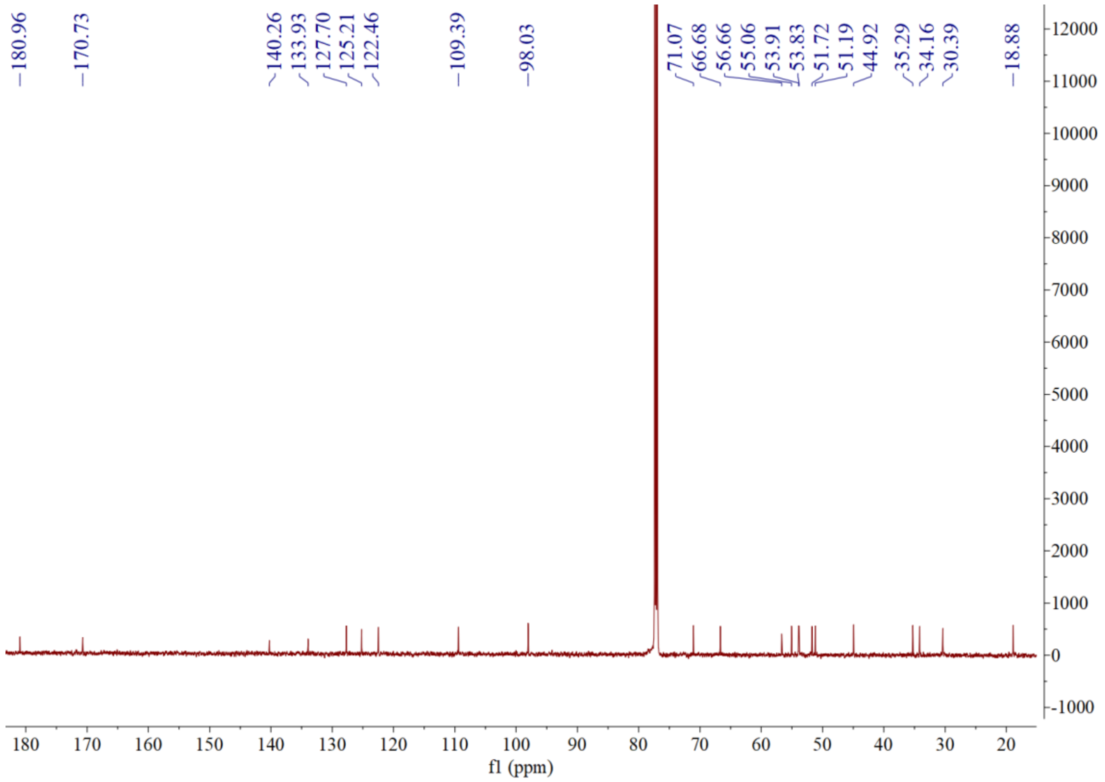

Figure S72. ^{13}C NMR spectra ($CDCl_3$, 150 MHz) of compound **11**

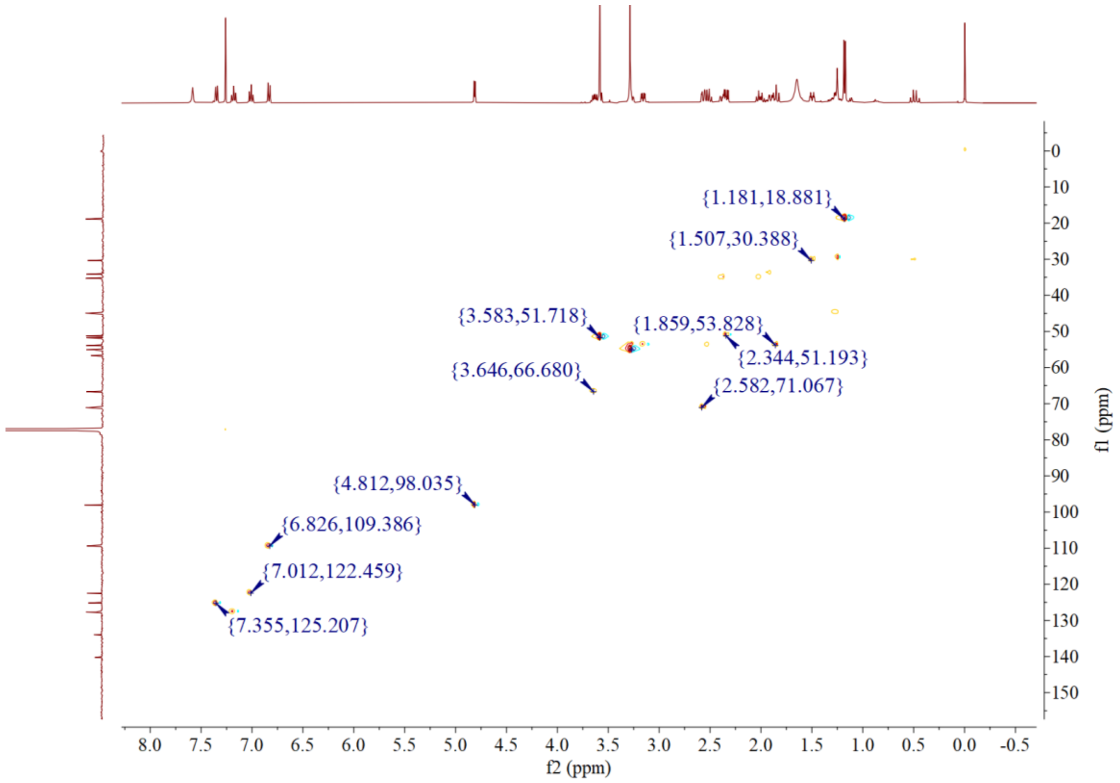

Figure S73. HSQC spectra ($CDCl_3$, 400 MHz) of compound **11**

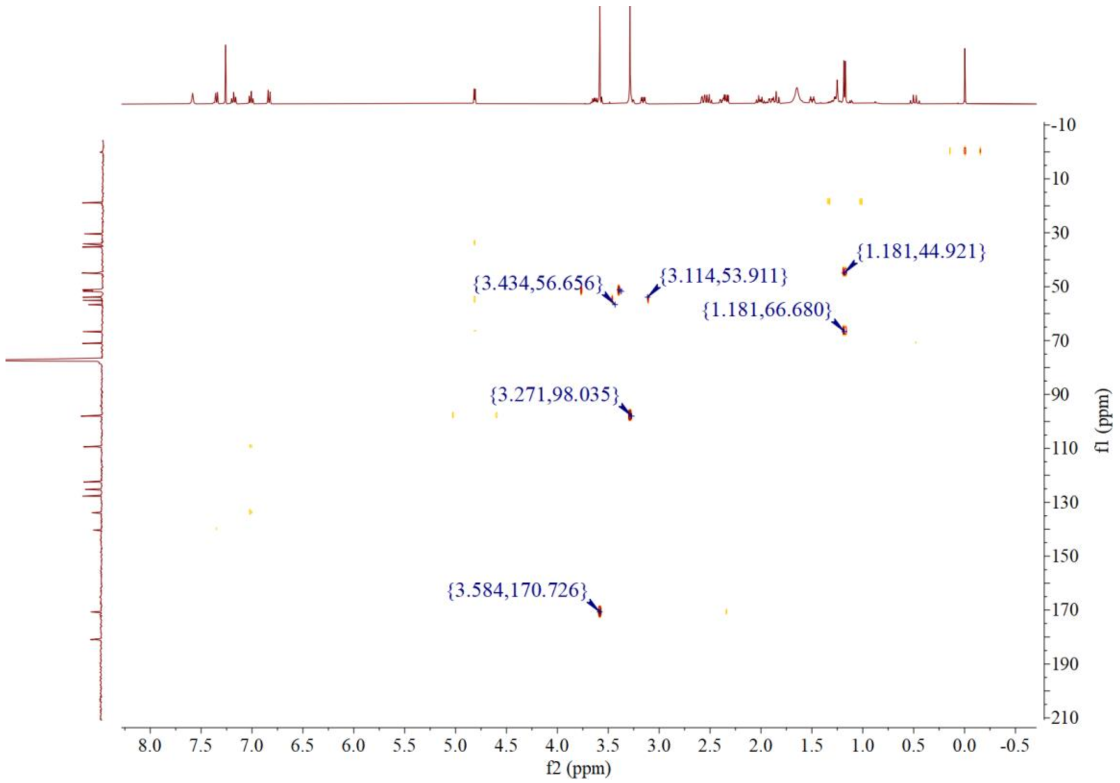

Figure S74. HMBC spectra ($CDCl_3$, 400 MHz) of compound **11**

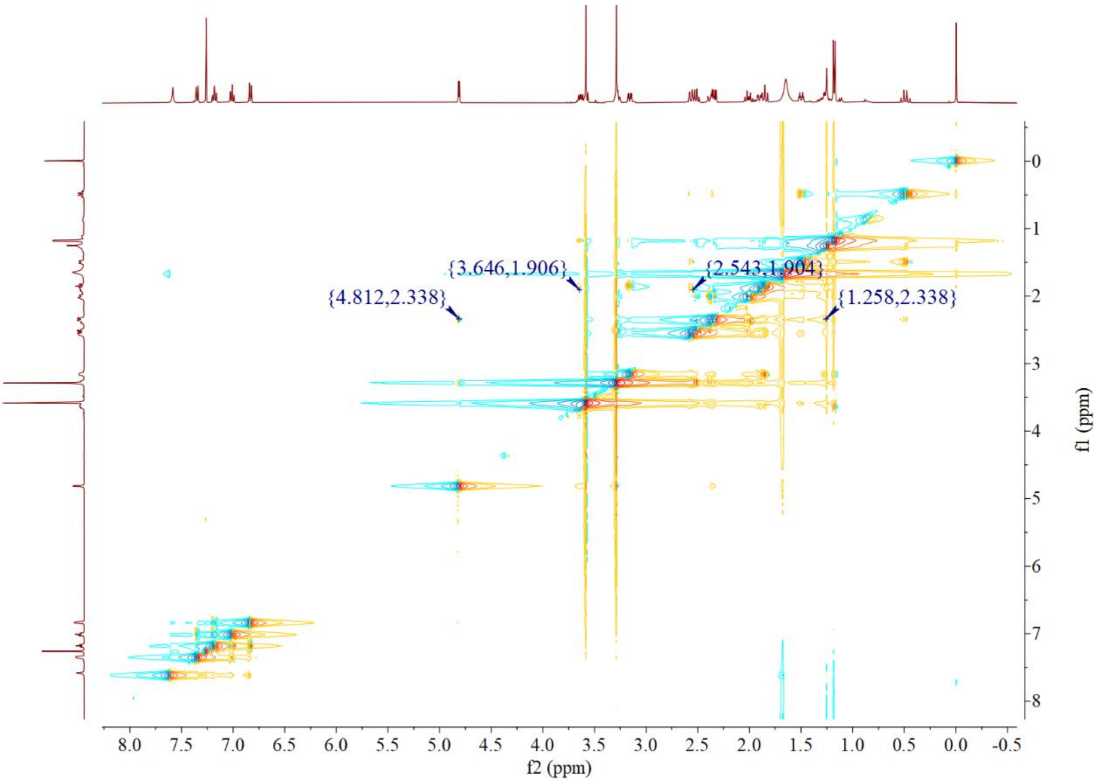

Figure S75. NOESY spectra ($CDCl_3$, 400 MHz) of compound **11**

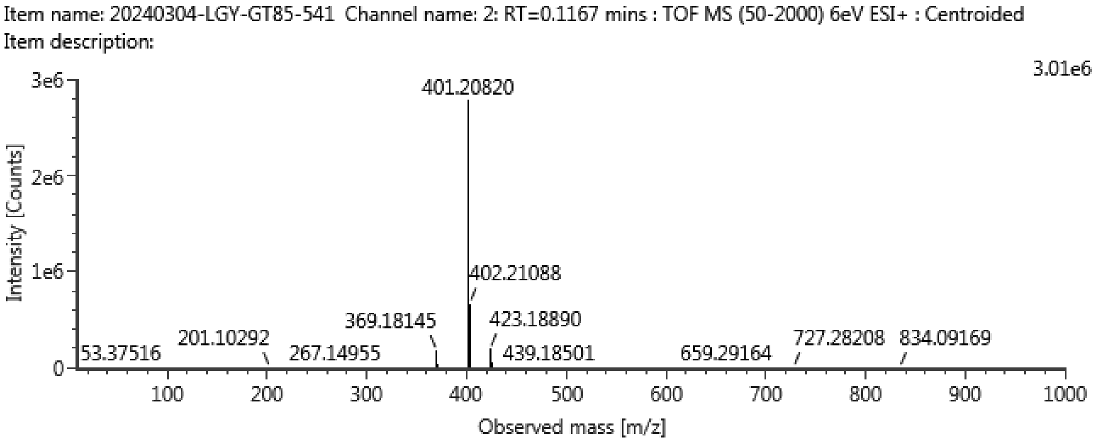

Figure S76. HRESIMS spectra of compound **11**

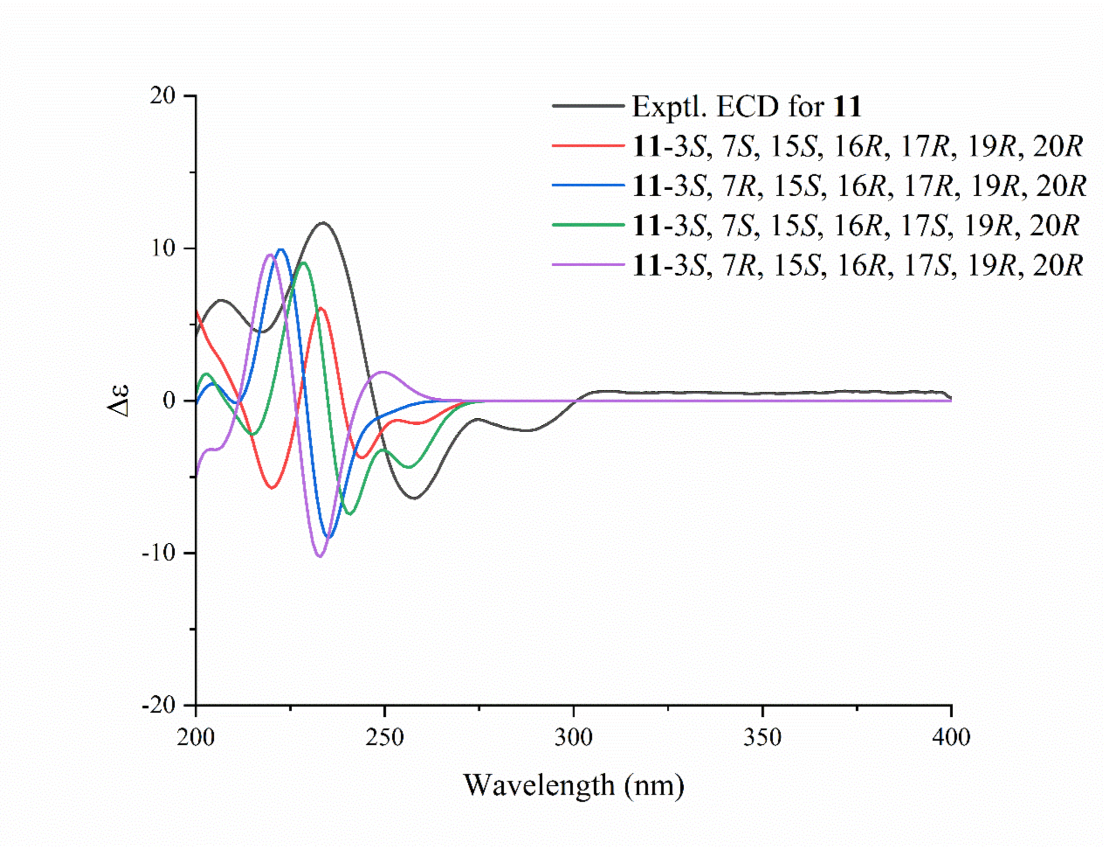

Figure S77. Experimental and calculated ECD spectra of compounds **11**

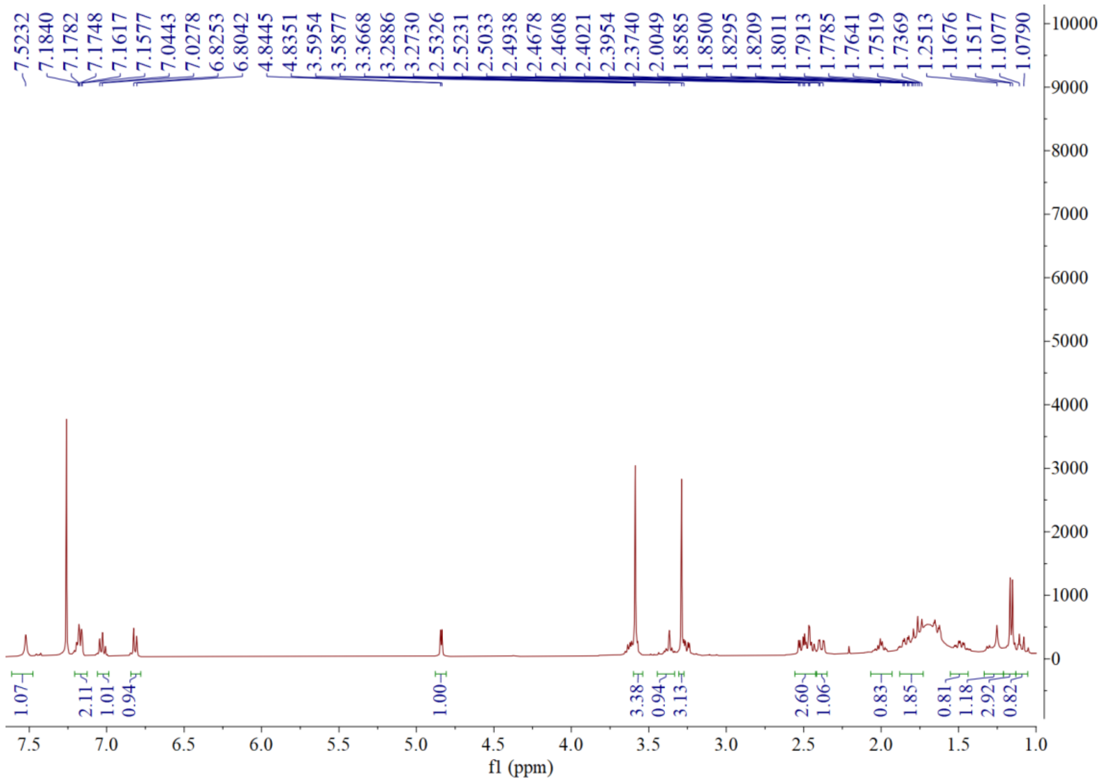

Figure S78. ^{1}H NMR spectra ($CDCl_3$, 400 MHz) of compound **12**

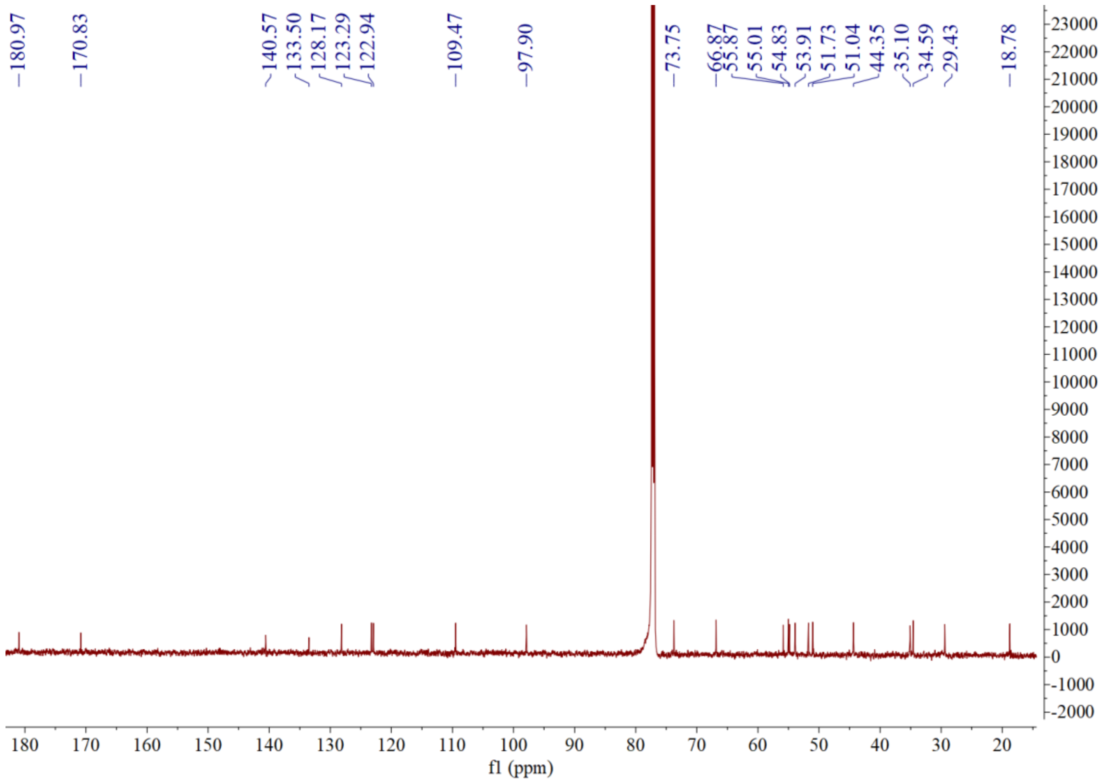

Figure S79. ^{13}C NMR spectra ($CDCl_3$, 150 MHz) of compound **12**

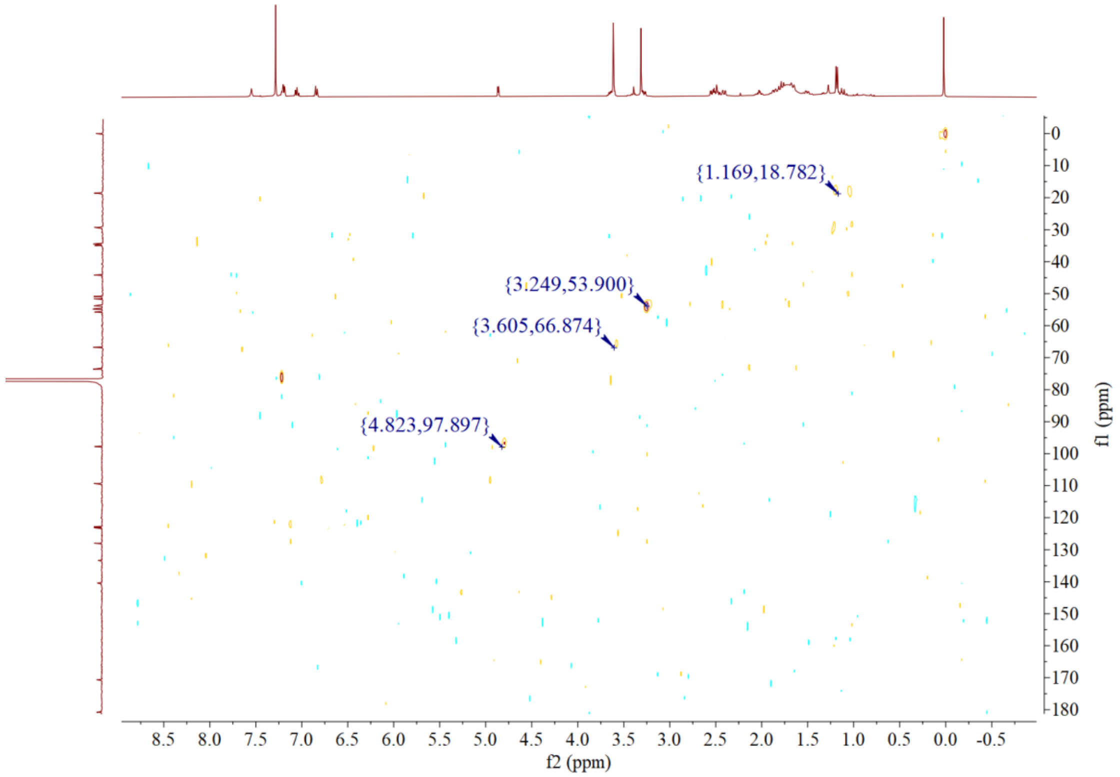

Figure S80. HSQC spectra ($CDCl_3$, 600 MHz) of compound **12**

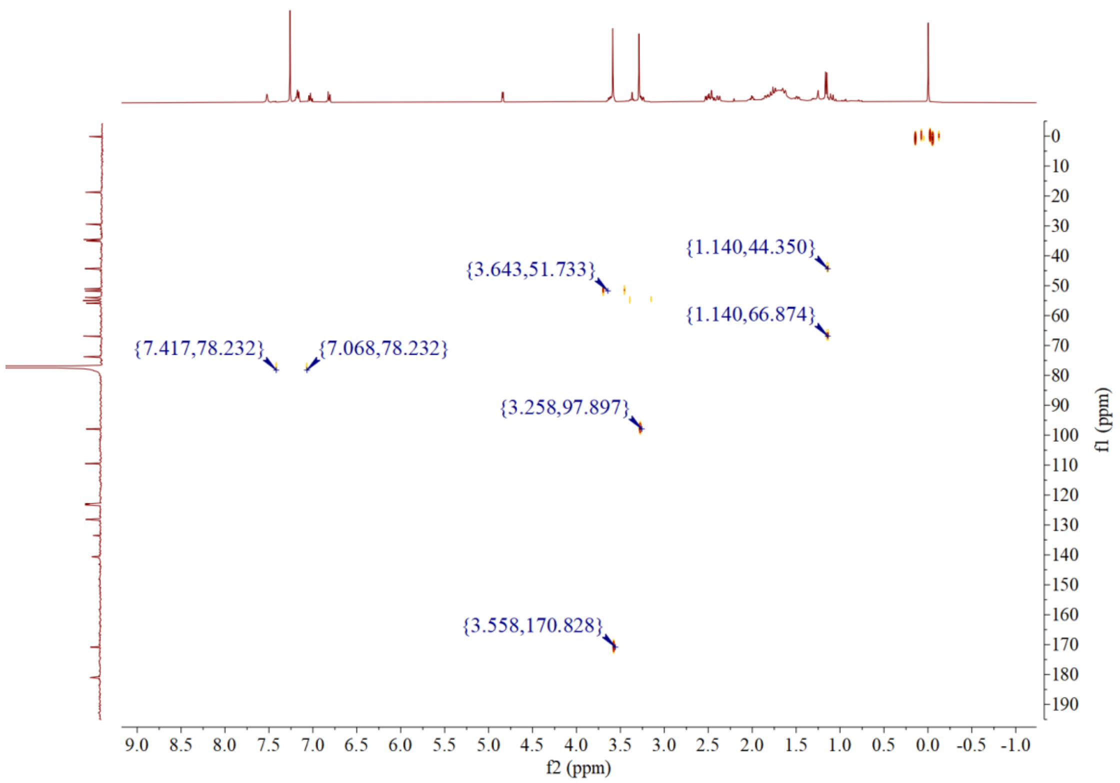

Figure S81. HMBC spectra ($CDCl_3$, 600 MHz) of compound **12**

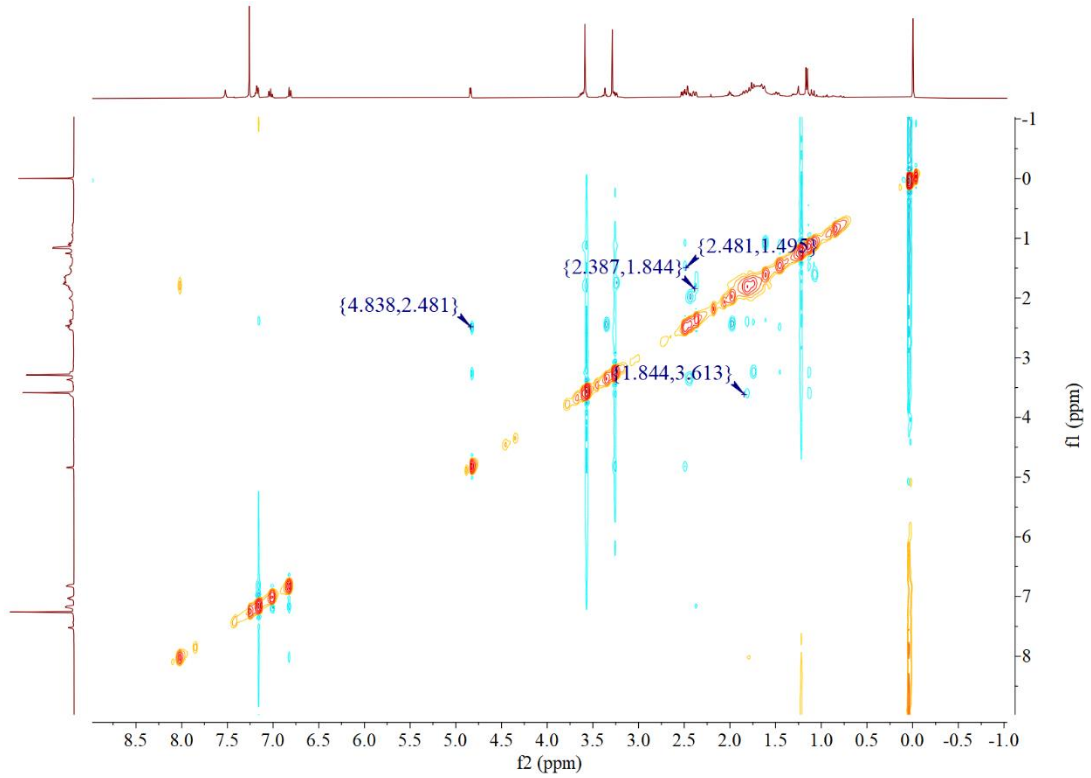

Figure S82. NOESY spectra ($CDCl_3$, 600 MHz) of compound **12**

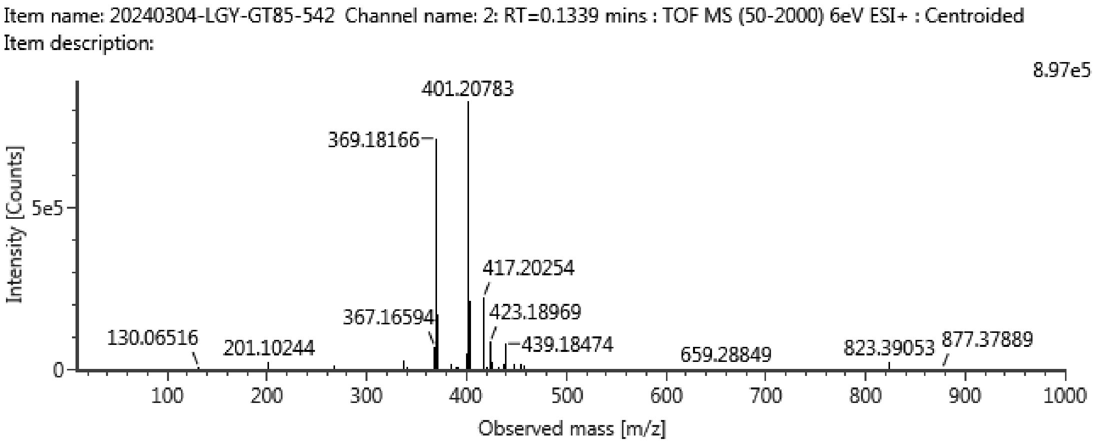

Figure S83. HRESIMS spectra of compound **12**

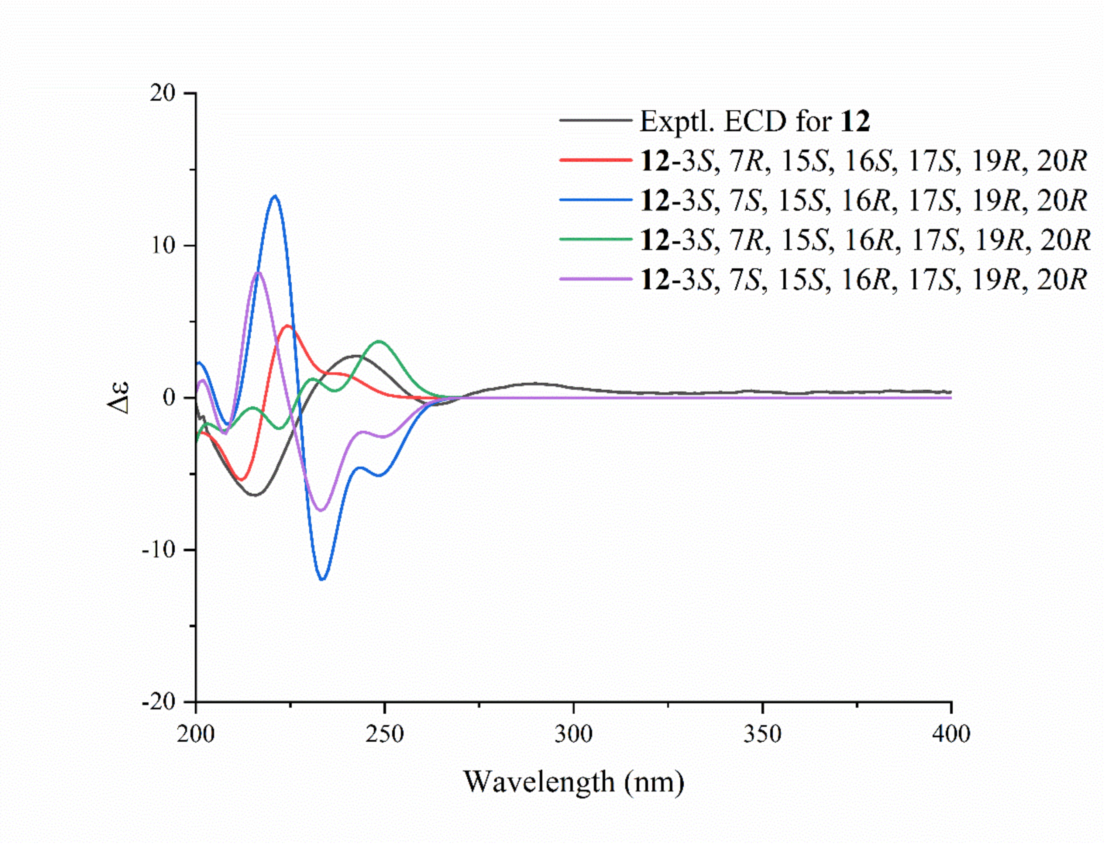

Figure S84. Experimental and calculated ECD spectra of compounds **12**
